# CryoRhodopsins: A comprehensive characterization of a group of microbial rhodopsins from cold environments

**DOI:** 10.1126/sciadv.adv1015

**Published:** 2025-07-04

**Authors:** Gerrit H. U. Lamm, Egor Marin, Alexey Alekseev, Anna V. Schellbach, Artem Stetsenko, Jose Manuel Haro-Moreno, Gleb Bourenkov, Valentin Borshchevskiy, Marvin Asido, Michael Agthe, Sylvain Engilberge, Samuel L. Rose, Nicolas Caramello, Antoine Royant, Thomas R. Schneider, Alex Bateman, Thomas Mager, Tobias Moser, Francisco Rodriguez-Valera, Josef Wachtveitl, Albert Guskov, Kirill Kovalev

**Affiliations:** ^1^ Institute of Physical and Theoretical Chemistry, Goethe University Frankfurt, 60438 Frankfurt am Main, Germany.; ^2^ Groningen Institute for Biomolecular Sciences and Biotechnology, University of Groningen, 9747AG, Groningen, Netherlands.; ^3^ Institute for Auditory Neuroscience and InnerEarLab, University Medical Center Göttingen, 37075 Göttingen, Germany.; ^4^ Advanced Optogenes Group, Institute for Auditory Neuroscience, University Medical Center Göttingen, 37075 Göttingen, Germany.; ^5^ Cluster of Excellence “Multiscale Bioimaging: From Molecular Machines to Networks of Excitable Cells” (MBExC), University of Göttingen, 37075 Göttingen, Germany.; ^6^ Else Kröner Fresenius Center for Optogenetic Therapies, University Medical Center Göttingen, 37075 Göttingen, Germany.; ^7^ Evolutionary Genomics Group, Departamento Producción Vegetal y Microbiología, Universidad Miguel Hernández, Alicante, Spain.; ^8^ European Molecular Biology Laboratory, EMBL Hamburg c/o DESY, 22607, Hamburg, Germany.; ^9^ Forschungszentrum Jülich GmbH, Wilhelm-Johnen-Straße, 52428 Jülich, Germany.; ^10^ Univ. Grenoble Alpes, CNRS, CEA, Institut de Biologie Structurale (IBS), 71 avenue des Martyrs, CS 10090, 38044 Grenoble Cedex 9, France.; ^11^ European Synchrotron Radiation Facility, 71 avenue des Martyrs, CS 40220, 38043 Grenoble Cedex 9, France.; ^12^ Hamburg Centre for Ultrafast Imaging, Universität Hamburg, HARBOR, Luruper Chaussee 149, 22761 Hamburg, Germany.; ^13^ European Molecular Biology Laboratory, European Bioinformatics Institute (EMBL-EBI), Wellcome Genome Campus, Hinxton, UK.; ^14^ Auditory Neuroscience and Synaptic Nanophysiology Group, Max-Planck-Institute for Multidisciplinary Sciences, 37075 Göttingen, Germany.; ^15^ Auditory Neuroscience and Optogenetics Laboratory, German Primate Center, 37075 Göttingen, Germany.

## Abstract

Microbial rhodopsins are omnipresent on Earth; however, the vast majority of them remain uncharacterized. Here, we describe a rhodopsin group found in microorganisms from cold environments, such as glaciers, denoted as CryoRhodopsins (CryoRs). A distinguishing feature of the group is the presence of a buried arginine residue close to the cytoplasmic face. Combining single-particle cryo–electron microscopy and x-ray crystallography with rhodopsin activation by light, we demonstrate that the arginine stabilizes an ultraviolet (UV)–absorbing intermediate of an extremely slow CryoRhodopsin photocycle. Together with extensive spectroscopic characterization, our investigations on CryoR1 and CryoR2 proteins reveal mechanisms of photoswitching in the identified group. Our data suggest that CryoRs are sensors for UV irradiation and are also capable of inward proton translocation modulated by UV light.

## Teaser

Spectroscopy, structural, and functional study of microbial rhodopsins with characteristic long-living near–UV-absorbing state.

## INTRODUCTION

Microbial rhodopsins (MRs) constitute a large family of light-sensitive membrane proteins found all over Earth in various organisms such as Archaea, Bacteria, Eukaryota, and viruses (*1*, *2*). Because of continuous sampling and metagenomic analysis, the phylogenetic tree of MRs is constantly expanding (*1*, *3*). This expansion affects both the known clades when not only more of their members are being identified and assigned to these clades but also the discovery of new groups of MRs, which may form distinct branches in the phylogenetic tree (*1*, *4*). The characterization of such MRs is crucial for fundamental science as it opens the door for revealing unusual molecular mechanisms of ion transport, light sensing, signal transduction, enzymatic activities, etc. Moreover, beyond the ecological, evolutionary, biochemical, and biophysical relevance, it is of great interest and importance also for biotechnological applications of MRs such as optogenetics (*5*). For instance, the recently discovered light-gated anion and cation channelrhodopsins (ChRs) have already entered use as highly effective optogenetic tools (*6*–*8*).

One approach to revealing clades of MRs uses bioinformatic search within the gene databases and sequence analysis to find proteins with unusual functional motifs or domain architecture. Typically, three- or six-letter motifs are analyzed, which include the residues in helices C and G at the positions of D85, T89, D96 or R82, D85, T89, D96, D212, and K216 in the classical representative of MRs, bacteriorhodopsin (BR) from *Halobactrium salinarum* (*9*). However, recent work has shown that there are other positions in MRs that help determine the function of the protein and may differ between the clades of a particular family. An example is the position of T46 in the helix B of BR, which is actively involved in the ion translocation process and can be occupied by glutamic acid in viral rhodopsins of groups 1 and 2 (*10*, *11*) or histidine in bacterial outward proton pumps (*12*, *13*).

In this work, we used bioinformatics and found MRs with unusual seven-letter motifs (corresponding to the residues T46, R82, D85, T89, D96, D212, and K216 of BR), which can be assigned to a separate clade in the phylogenetic tree. This clade, characterized by the presence of an arginine residue at the position of T46 in BR, has never been described before to our knowledge. The members of the clade are restricted to cold environments and are found in cold-living bacteria, such as *Cryobacterium*, *Subtercola* sp., and others. Therefore, we named this clade CryoRhodopsins (CryoRs). We show that CryoRs have extremely slow photocycles dominated by a blue-shifted ultraviolet (UV)–absorbing intermediate state. High-resolution single-particle cryo–electron microscopy (cryo-EM) and x-ray crystallography on two CryoRs demonstrated three key roles of the characteristic arginine in helix B in the stabilization of this intermediate. Moreover, we demonstrate that CryoRs vary in their spectral properties. For instance, some of them demonstrate a strong pH dependence of the absorption spectra and the photocycle kinetics, while for others, it is negligible. Notably, one rhodopsin (CryoR1 from *Cryobacterium levicorallinum*) has several absorption maxima over a wide range of pH values and showed a 80-nm red shift to 620 nm below pH 6. Furthermore, CryoR1 exhibits strong coherent oscillations on the ultrafast timescale in its 620-nm absorbing state at low pH indicating a hindered isomerization of the retinal chromophore. Electrophysiology of CryoR1 revealed light-driven inward proton pumping activity of the protein modulated by UV light. We also demonstrate the use of cryo-EM for the determination of high-resolution structures of membrane proteins such as rhodopsins in the intermediate states following light activation to uncover the molecular mechanisms of the CryoRs work. Genomic context analysis indicates the presence of putative cytoplasmic transducers of CryoRs, suggesting a biological role for these rhodopsins as sensors for harmful UV light to which the source organisms are likely to be exposed.

## RESULTS

### Identification of CryoRs

**Fig. 1.**
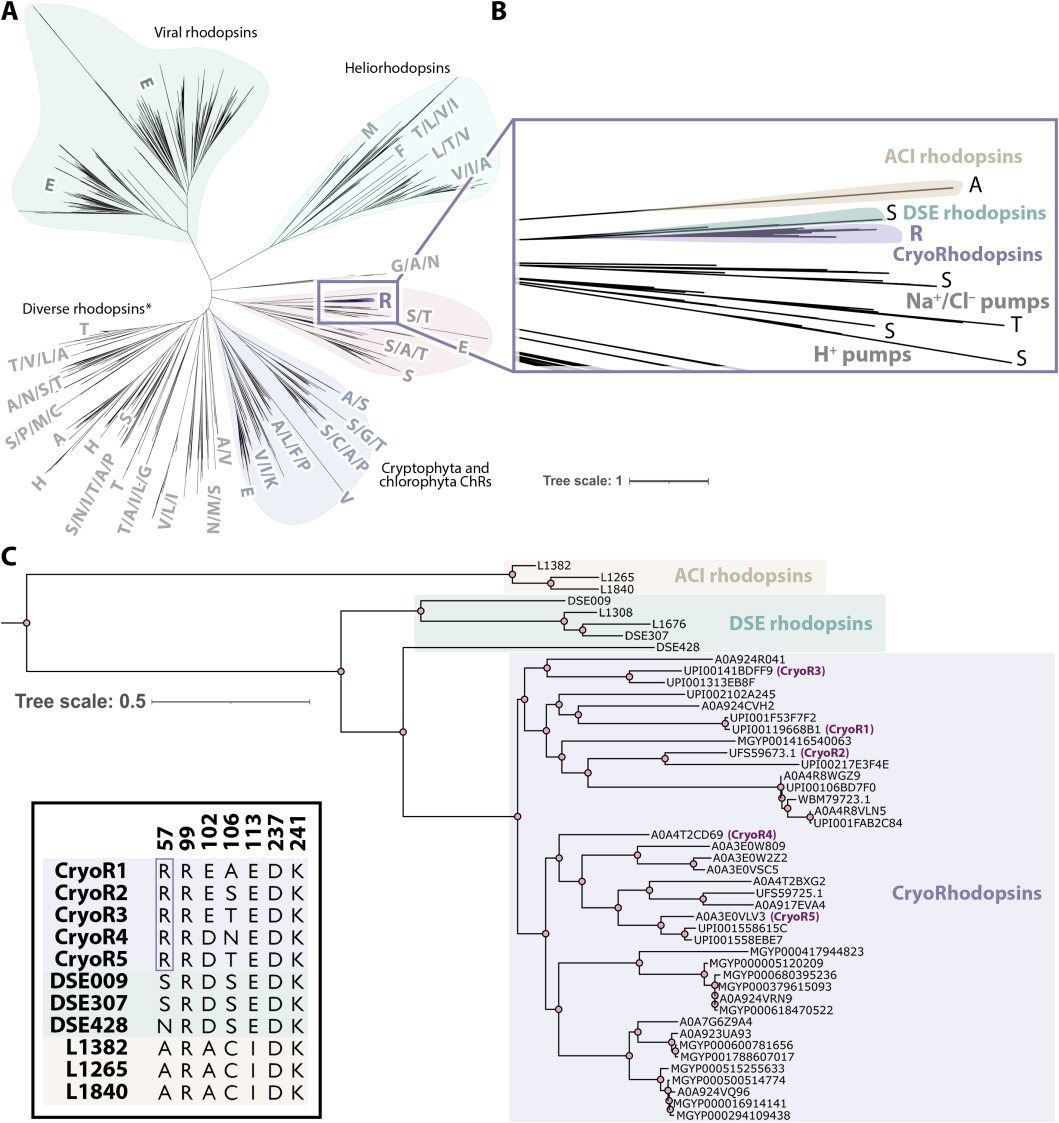
Phylogeny of CryoRs. (**A**) Maximum likelihood phylogenetic tree of MRs. The tree includes 2199 sequences reported in (*1*), 3 sequences of DSE rhodopsins reported in (*17*), and 40 sequences of CryoRs found in the present work. (**B**) Enlarged view of the tree branch containing CryoRs. Amino acid residues in helix B at the position corresponding to that of T46 in BR are shown at the tips. (**C**) Rectangular representation of the phylogenetic tree of the CryoRs and nearby DSE and ACI rhodopsins clades. The inset in the left bottom corner shows amino acids of the seven-letter motifs of CryoR1-5, DSE, and ACI rhodopsins (numbering corresponds to CryoR1). The unique arginine (R57 in CryoR1) is boxed for clarity.

To identify unusual clades of MRs, we performed a bioinformatic search within the UniProtKB, UniParc, GenBank, and MGnify gene databases and analyzed a seven-letter motif of the output protein sequences. As a result, we found MRs with motif pattern RRXXXDK, where XXX varied between EAE, ESE, ETE, DSE, DNE, DTE, GSE, and GTQ. In total, we identified 40 unique complete protein sequences with these functional motifs, which cluster together in the phylogenetic tree (Fig. 1, A and B).

The members of this group of MRs were found only in microbes belonging to the actinobacterial family Microbacteriaceae and described as isolated from cold environments (table S1), specifically ice or snow surfaces or high altitude regions (*14*, *15*). Thus, we named this separate group CryoRs to reflect their link to microbes from low-temperature environments. In addition to their origin, most cultures of both *Cryobacterium* and *Subtercola* show a growth behavior consistent with them being adapted to cold environments, e.g., low optimal or maximum growth temperatures (*14*, *15*). We should also note that the species distribution of CryoRs may be biased by the sequences present in public databases due to the predominance of samples of convenience.

Phylogenetically, CryoRs are located closely to the branch of the bacterial proton, sodium, and chloride pumps (Fig. 1, A and B). It should be noted that rhodopsins with EAE motif appeared in the phylogenetic tree in (*16*) and were assigned to the P1 family of MRs together with the recently reported group of DSE rhodopsins (*17*) and some other previously studied proteins with DTE motif (*18*–*20*). The closest relatives of CryoRs are DSE rhodopsins (Fig. 1, B and C) (*17*). The analysis of the protein sequences showed that both CryoRs and DSE rhodopsins have common features such as elongated N and C termini (fig. S1); however, DSE rhodopsins do not have the arginine found in the helix B of CryoRs (Fig. 1C). Moreover, DSE rhodopsins do not show any associations with cold environments. Thus, we conclude that the arginine residue is a unique characteristic feature of CryoRs. No MRs with an arginine residue at the position of T46 in BR have been reported. The next closest relatives are rhodopsins with the unusual ACI motif (*1*), which differ from both the DSE rhodopsins and CryoRs (Fig. 1, B and C).

Most CryoRs (39 of 40) have a glutamic acid residue at the position corresponding to that of a primary proton donor to the retinal Schiff base (RSB), D96 in BR (*21*). This position is occupied by a glutamic acid acting as a proton donor in the most light-driven bacterial outward proton pumps (*3*). In contrast to the arginine and glutamic acid at the cytoplasmic side, the set of two key residues of the functional motif located closely to the RSB in the central region of the rhodopsin varies largely within the clade. Nevertheless, 38 of 40 CryoRs have a carboxylic residue (15, glutamic acid; 23, aspartic acid) at the position of a primary proton acceptor D85 in BR (*22*). All CryoRs have an aspartic acid in helix G at the position of D212 in BR. Thus, most CryoRs have two RSB counterions, similar to BR and many other MRs. The amino acid residue at the position of T89 in BR is occupied by alanine (*8*), serine (*7*), threonine (*23*), or asparagine (*1*).

For an in-depth investigation, we selected five representatives of the CryoR clade with different seven-letter motifs: RREAEDK (CryoR1, *C. levicorallinum*, GenBank ID: WP_166787544), RRESEDK (CryoR2, *Subtercola* sp. AK-R2A1-2, GenBank ID: UFS59673.1), RRETEDK (CryoR3, *Cryobacterium frigoriphilum*, GenBank ID: WP_166791776), RRDNEDK (CryoR4, *Subtercola vilae*, UniProt ID: A0A4T2CD69), and RRDTEDK (CryoR5, *Subtercola boreus*, UniProt ID: A0A3E0VLV3) (Fig. 1C). Below, we describe the common spectral and structural features of CryoRs originating from the presence of the arginine residue at their cytoplasmic side as well as the differences between the CryoRs and their unique properties.

### Slow photocycle kinetics of CryoRs dominated by blue-shifted intermediate

Since CryoRs mainly originate from cold environments, a strong temperature dependence going beyond the usually expected de- or acceleration of the kinetics could have been expected. This was not confirmed experimentally: For example, comparison of dark-state spectra of CryoR1 at 20° and 5°C, respectively, showed no indication for such effects (fig. S2). Thus, if not explicitly stated, experimental data were acquired at room temperature.

**Fig. 2.**
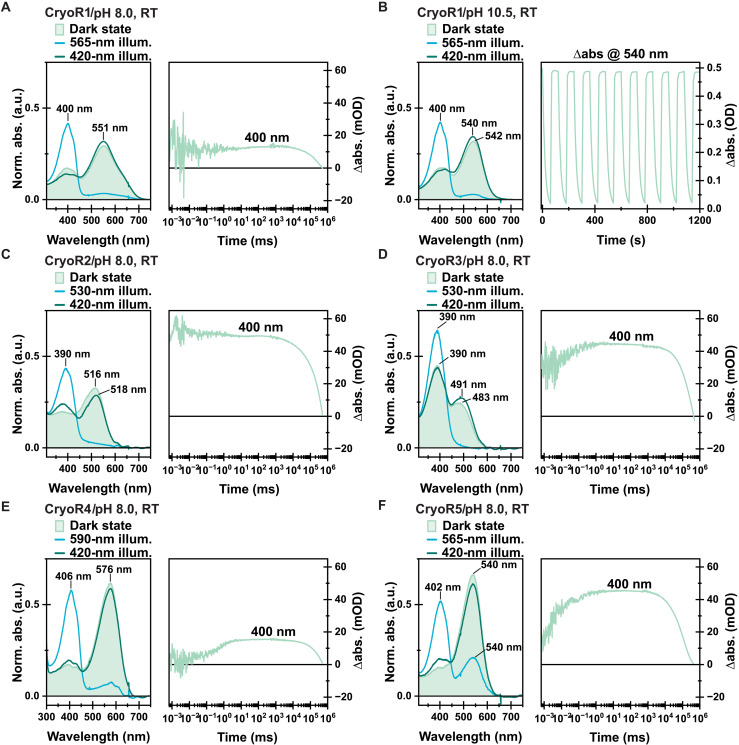
Illumination experiments and M state kinetics of CryoRs. (**A** and **C** to **F**) Dark-state spectra (light green), as well as the PSS spectra after illumination of the main absorption band (blue), the spectra after illumination of the obtained PSS with blue light (dark green) to recover the dark state of each investigated CryoR at pH 8.0. Each LED was turned on for 100 s. The respective output powers are provided in Materials and Methods. In addition, the flash photolysis transient at 400 nm is shown for each protein to elucidate the unusually slow kinetics of the M intermediate and the whole photocycle. (**B**) Illumination experiments of CryoR1 at pH 10.5 including an alternating illumination scheme of green and blue-light to test for potential photobleaching and photofatigue. a.u., arbitrary units; RT, room temperature; mOD, milli optical density; abs., absorbance.

CryoR1-5 differ in their UV–visible (Vis) absorption spectra with the main band position varying in the range of 390 to 580 nm (Fig. 2 and fig. S3A). However, the studied proteins have a common feature in their spectral behavior, which is the presence of a long-living blue-shifted intermediate state in the photocycle (Fig. 2). The intermediate lasts for minutes and accumulates in the samples upon illumination as indicated by a color change of all studied CryoRs when exposed to the overhead light in the laboratory. To investigate the photoconversion of CryoRs in more detail, we performed illumination experiments of CryoR1-5 using light-emitting diodes (LEDs) with central wavelengths corresponding to the main absorption band in the green-to-red spectral area. Before conducting spectroscopic experiments, samples were kept in the dark for days to prevent accumulation of a long-living blue-shifted intermediate caused by the exposure to light and a slow decay rate.

For neutral to alkaline conditions, illumination of all studied CryoRs with LEDs in the range of 530 to 590 nm, tailored to the absorption maximum of the respective protein, resulted in a photostationary state (PSS) with an almost completely depopulated main absorption band and a newly formed UV signal centered at ~400 nm (Fig. 2 and fig. S3B). At pH 8.0, for all CryoRs except CryoR3, the PSS peak shows a clearly resolved fine structure with three distinguishable maxima. As shown for some other MRs, such a retro-retinyl–like absorption spectrum most likely originates from an enforced planarity of the retinal chromophore (*23*–*25*). Illumination of the PSS with a 420-nm LED resulted in the recovery of the dark-state spectrum, which is well known for MRs as the blue-light quenching (BLQ) effect (Fig. 2) (*26*–*29*). Alternating illumination of the main absorption band and the populated PSS resulted in no photofatigue or photobleaching, allowing the usage of BLQ to repopulate the parent state during experiments and potential applications in the future (Fig. 2B).

Without BLQ, CryoRs show unusually slow photocycle kinetics dominated by a blue-shifted intermediate and need several minutes to recover their parent state (Fig. 2). Thermal relaxation of the upon illumination-formed PSS was monitored via the intensity of the initial absorption maximum at 554-nm resolving recovery lifetimes of 942 s (20°C) and 3318 s (5°C), respectively (fig. S2). This is a characteristic feature of all studied CryoRs with various motifs. Thus, we propose it to be a common feature of the entire group. We note that decelerated photocycle kinetics of proteorhodopsins (PRs) found in cold-living bacteria compared to those of classical marine PRs has been observed in the past (*30*). However, in that case, the photocycle was within a 7.5-s time range, which is notably faster than those of CryoRs (minutes). In addition, the photocycle of the rhodopsins described in (*30*) is dominated by a late red-shifted O intermediate, not found in CryoRs. Among all studied MRs that are not considered as bistable, CryoRs exhibited by far the longest lifetime of the blue-shifted UV-absorbing intermediate, corresponding to the deprotonated RSB (fig. S4). The decay of the M state in CryoRs is more than an order of magnitude longer than what was shown for any other MR to date (fig. S4). Noteworthy, the UV-absorbing state is accumulated in CryoR1 by ambient light in the laboratory or by sunlight (fig. S2, D and E). This indicates that under physiological conditions in the native source organisms, CryoRs might also be accumulated in the blue-shifted state and thus be sensitive to UV light.

**Fig. 3.**
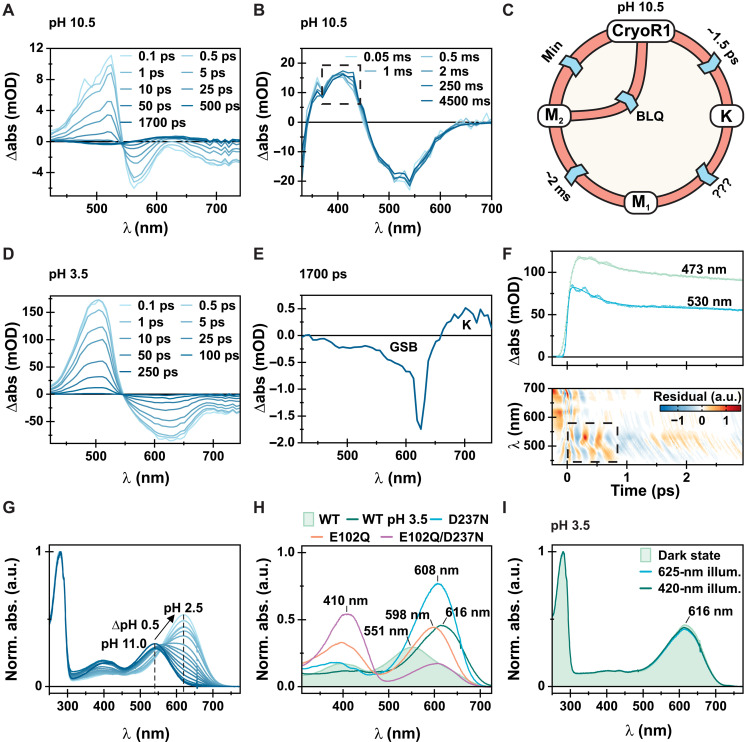
Spectroscopic characterization of CryoR1. (**A**) Femtosecond TA measurement of CryoR1 at pH 10.5, shown as difference absorption spectra at specific time points after excitation. (**B**) Flash photolysis measurement of CryoR1 at pH 10.5 shown as difference absorption spectra at specific time points after excitation. Dashed boxes illustrate differences between M_1_ and M_2_. The photocycle was quenched after 5 s via illumination with a 405-nm LED (8.71 mW) before each acquisition. (**C**) Photocycle model for CryoR1 at pH 10.5. (**D**) Femtosecond transient absorption (TA) measurement of CryoR1 at pH 3.5 presented similarly as in (A). (**E**) Transient spectrum taken from the femtosecond TA measurement shown in (D), showing the small amplitude for K and GSB once the photocycle is entered. Amplitudes at ~620 nm are reasoned by scattering of the excitation beam. (**F**) Transients at two wavelengths (473 and 530 nm) taken from an femtosecond TA measurement at pH 3.5 performed with a linear timescale ending at 3 ps and a decreased linear step size compared to the measurement shown in (D). The raw data are displayed as dots and the obtained fit as lines to illustrate the observed coherent oscillations. Furthermore, the residuals of the raw data and the fit are shown. The region of interest is highlighted with the dashed box. (**G**) pH titration of the CryoR1 dark-state absorption spectrum from pH 2.5 to 11.0. Spectra are normalized with respect to the absorption band at 280 nm. (**H**) Dark-state absorption spectra of CryoR1 WT, D237N, E102Q, and E102Q/D237N mutants. (**I**) Illumination experiments of CryoR1 performed at pH 3.5. The dark state was first illuminated with a 625-nm LED to check for a potential M state formation and with a 420-nm LED afterward to check for a potential BLQ effect.

In CryoR1, there are two such blue-shifted intermediates, M_1_ and M_2_, with lifetimes of milliseconds and minutes, respectively (Fig. 3B and fig. S5). The M_2_ state is slightly red-shifted compared to the M_1_ intermediate (Fig. 3B and fig. S5). The M_1_ state is already populated at the beginning of the experimentally accessible flash photolysis timescale (Fig. 3B). Thus, the formation of the M_1_ state is accelerated compared to most MRs, where this transition happens on the microsecond to millisecond timescale (*23*, *31*, *32*). Ultrafast spectroscopy data showed the formation of a red-shifted K intermediate preceding the M_1_ state (Fig. 3A and fig. S5). Because of the experimental time gap on the nanosecond timescale, it cannot be determined whether the M_1_ intermediate is directly populated after the K state, or if there is a multistep K-to-M_1_ transition, as it was observed for various MRs previously, e.g., *Ns*XeR (Fig. 3, A and B) (*24*). The resulting CryoR photocycle scheme is depicted in Fig. 3C and appears similar to the reported photocycle of DSE rhodopsins (*17*).

At acidic pH, the photocycle kinetics is accelerated for all studied CryoRs except CryoR3 (fig. S6). Moreover, the spectral changes observed upon illumination are less pronounced compared to neutral and alkaline conditions (fig. S6). Specifically, CryoR1, CryoR2, CryoR4, and CryoR5 show only minor changes in the absorption spectrum upon illumination of the main absorption band. The exception is CryoR3, still showing a formation of the blue-shifted PSS but less extensive compared to neutral and alkaline conditions (fig. S6).

### Unique spectral behavior of CryoR1

Among the studied CryoRs, CryoR1 shows several unique spectroscopic features, both in steady-state and time-resolved experiments. Therefore, we investigated the spectroscopic properties of CryoR1 in more detail.

The first feature is the ultrafast dynamics of CryoR1, being unusual at all studied pH values. At high pH, the ultrafast dynamics are characterized by strong excited-state–related signals, a prolonged excited-state lifetime accompanied by a biexponential decay (~0.4 and ~10 ps), and weak photoproduct formation. All of these features resemble the trends observed for various known MRs at low pH conditions or for their counterion mutants (Fig. 3A) (*33*–*38*). Directly after excitation of the retinal chromophore, the excited-state absorption (ESA) (420 to 540 nm), ground-state bleach (GSB) (540 to 625 nm), and stimulated emission (SE) (625 to 740 nm) are observed. The ESA decays biexponentially (~0.4 and ~10 ps), with the faster lifetime showing the K intermediate formation in the range of ~600 to 675 nm. In contrast to many known MRs, the typical J intermediate is missing. Explanations for the observations have been provided in the literature (*36*), although their validity in the context of CryoR1 is dubious due to its unique spectral properties, illustrated in this section.

At low pH, the ultrafast dynamics again show ESA, GSB, and SE signals but are dominated by a long-living ESA also decaying biexponentially (~0.5 and ~25 ps) as described by two-lifetime distributions in the corresponding lifetime distribution map (LDM) (Fig. 3D and fig. S5A). However, the formation of the K intermediate is negligible (Fig. 3, D and E). The wings of the ESA and GSB signals show strong coherent oscillations with two major frequency contributions, 32 and 128 cm^−1^ (Fig. 3F and fig. S7). The 32-cm^−1^ frequency corresponds to an oscillation period longer than the duration of the observed coherent oscillations. Therefore, this frequency is assigned to be an artifact of the Fourier analysis. The 128-cm^−1^ mode additionally shows contributions from slightly lower or higher frequencies (Fig. 3F). Frequencies in this range have been linked to low-energy torsional modes of the C═C double bonds (*39*, *40*). The strength of the coherent oscillations suggests a hindered retinal isomerization after excitation, in agreement with the extremely small signal amplitudes of the K state (Fig. 3, D and E).

The second feature of CryoR1 is an unusually large spectral red shift of ~80 nm upon pH titration in the range of pH 11.0 to pH 2.5 (Fig. 3G and fig. S7). Specifically, at pH < 6, the spectrum is dominated by the red-shifted band at 620 nm (Fig. 3G). In addition, under alkaline conditions, a second absorption band centered at 400 nm arises in the CryoR1 spectrum, which likely corresponds to the ground-state species with a deprotonated RSB, due to its rise at low proton concentrations (*41*–*43*). In contrast to many known MRs, the pH-dependent spectral shift of CryoR1 is not observed in a continuous movement across the spectral range (*11*, *38*, *44*) but rather in a gradual decrease of the red-shifted absorption maximum at 620 nm associated with a rise of the blue-shifted absorption maximum at 540 to 550 nm and the deprotonated RSB band (400 nm). This behavior resembles the pattern observed for Tara-RRB, a member of bestrhodopsin clade of MRs (*45*).

Usually, pH-dependent shifts of the absorption maximum are associated with the change in protonation of the RSB and/or charged residues in the retinal binding pocket. Neutralization of such charged residues results in a red shift of the absorption maximum, as it was observed for various MRs in the past (*23*, *34*, *36*). Since in CryoR1 there are two RSB counterions, E102 and D237, we investigated the spectral properties of its E102Q, D237N, and E102Q/D237N variants (Fig. 3H and fig. S8). None of the substitutions was sufficient to mimic the red shift of the absorption maximum observed in the wild-type (WT) protein upon pH decrease (Fig. 3I). The absorption maximum of the mutants was blue-shifted (10 to 15 nm) compared to that of WT at low pH. Therefore, the unique large spectral shift shown for CryoR1 cannot be simply described by the protonation of the RSB counterions, and the molecular basis of this shift is further described in the “Protonation states of the counterions in CryoRs” section of the Supplementary Text.

### Structural organization of CryoRs

**Fig. 4.**
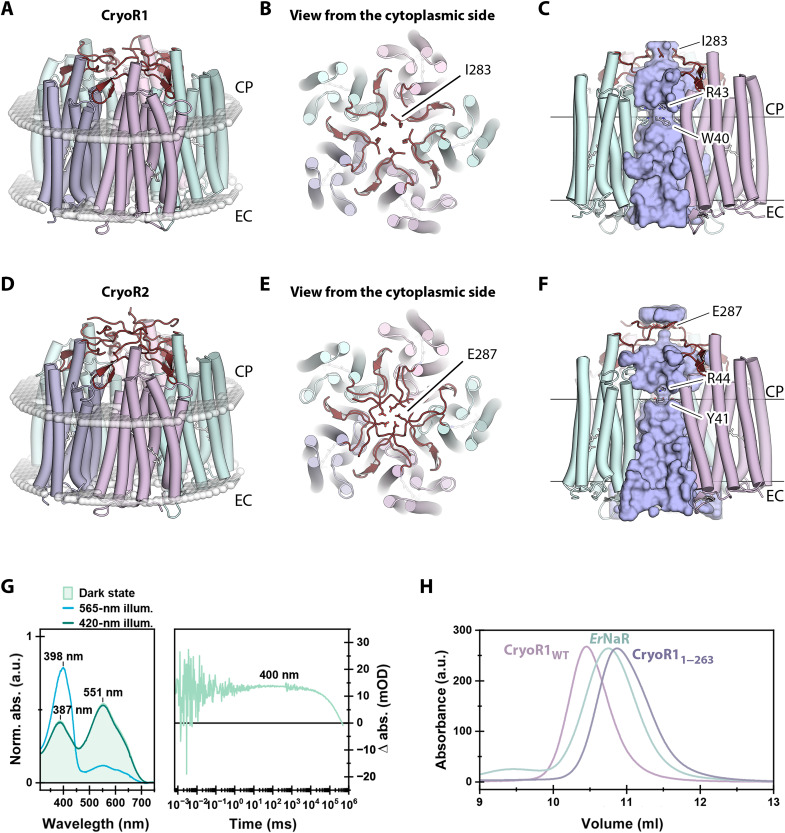
Pentameric architecture of CryoRs and unusual central channel. (**A**) Overall view of the CryoR1 pentamer in detergent micelles and nanodiscs. (**B**) View at the CryoR1 pentamer from the cytoplasmic side. (**C**) Side view of the central channel in CryoR1. (**D**) Overall view of the CryoR2 pentamer in crystals. (**E**) View at the CryoR1 pentamer from the cytoplasmic side. (**F**) Side view of the central channel in CryoR1. C terminus is colored dark red. (**G**) Spectroscopy of CryoR1_1–263_. Left: The dark-state spectra (light green), as well as the PSS spectra after illumination of the main absorption band (blue), the spectra after illumination of the obtained PSS with blue light (dark green) to recover the dark state of CryoR1_1–263_ at pH 8.0. The 565-nm LED was turned on for 100 s (1.05 mW), while the 420-nm LED turned on for 100 s (0.19 mW). Right: The flash photolysis transient at 400 nm. (**H**) Size exclusion chromatography (SEC) profiles of pentameric CryoR1_WT_ [molecular weight (MW): 180.6 kDa] and CryoR1_1–263_ (MW: 147.2 kDa). SEC profile of the pentameric light-driven sodium pump *Er*NaR (MW: 164.0 kDa) measured under the same conditions using the same column is shown as a reference. CP, cytoplasmic side; EC, extracellular side.

To gain insights into the architecture of CryoRs and their unique spectral properties, we obtained high-resolution structures of CryoR1 and CryoR2 using single-particle cryo-EM and x-ray crystallography (Fig. 4, figs. S9 and S10, table S2, and Supplementary Text). Both proteins form pentamers in detergent micelles, lipid nanodiscs, and crystals grown in lipidic cubic phase (LCP) (Fig. 4A and Supplementary Text). An elongated C terminus is capping the central part of the oligomer at the intracellular side (Fig. 4, B and E). The C-terminal elongation is conserved across CryoRs (fig. S1). With this cap, the profile of the central pore of the CryoR pentamer is unusual compared to those of other known MRs (Fig. 4, C and F). The transmembrane part is mostly hydrophobic and occupied by lipid molecules as evidenced in the (electron) density maps but has a narrow polar region near the W40 and R43 residues in CryoR1 (Y41 and R44 in CryoR2) (Fig. 4, C and F), which, to some extent, resembles that of the pentameric viral rhodopsin OLPVRII (*10*). Next, the cytoplasmic part of the pore in CryoRs has an additional second narrowing formed by the C-terminal residues I238 and E287 in CryoR1 and CryoR2, respectively (Fig. 4, B, C, E, and F). The pore is completely blocked in this area in CryoR2 at acidic pH by five glutamic acid residues (E287) (Fig. 4F). The region between the two narrowings represents an aqueous basin with numerous water molecules interacting with the surface of this part of the central channel. Despite the fact a weak proton transport activity has been observed for CryoR1, as described further in the manuscript, the contribution of such an unusual central channel and the unique cap formed by the C terminus for this function remain elusive. Moreover, as also discussed further, the C terminus of CryoRs might interact with putative transducers thus being involved in the signaling mechanism of the rhodopsins.

Last, although the C terminus interacts with the nearby protomer within the oligomeric assembly and thus likely stabilizes the pentamer (fig. S11), both the spectral properties of the truncated variant of CryoR1 (CryoR1_1–263_, 1 to 263 amino acid residues) including the long-living blue-shifted state as well as the oligomeric state are similar to those of the WT protein (Fig. 4, G and H). Therefore, the presence of the elongated C terminus is likely not connected to the unique photocycle kinetics of CryoRs.

**Fig. 5.**
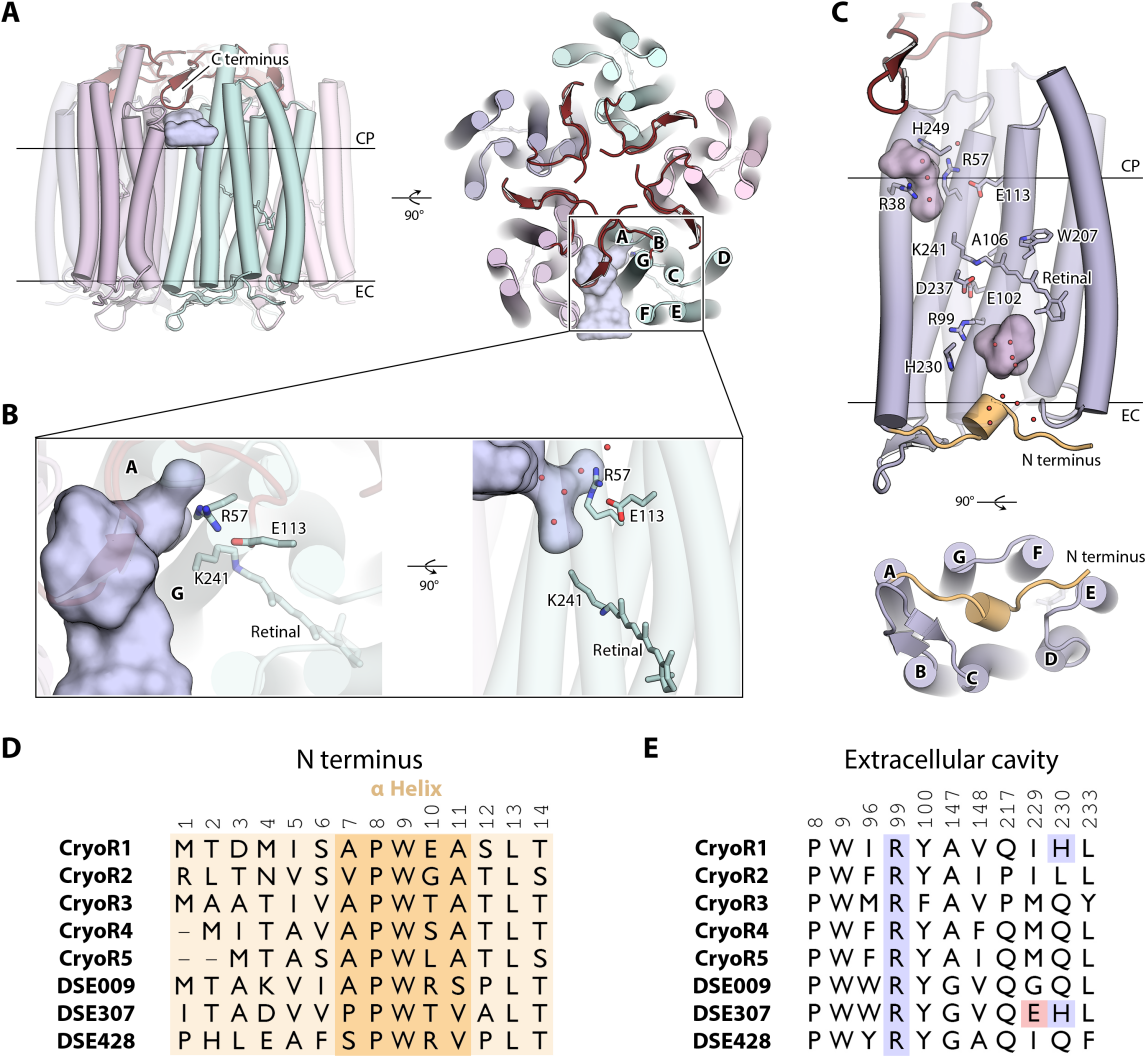
Structural features of the CryoR protomer. (**A**) Side view (left) and view from the cytoplasmic side (right) of the CryoR1 pentamer and the interprotomeric cavity (shown with light blue surface). (**B**) Detailed view of the interprotomeric cavity and the internal cytoplasmic cavity in CryoR1. (**C**) Side view (top) and view from the extracellular side (bottom) of the protomer of CryoR1. N terminus is colored wheat. The small α helix in the N terminus is shown as a cylinder. Internal cavities are colored light pink. For the representation, the cryo-EM structure of CryoR1 at pH 8.0 was used. The structural date has been obtained at 80 K. (**D**) Sequence alignment of the N-terminal region of representative CryoRs and DSE rhodopsins. (**E**) Alignment of the amino acids surrounding the extracellular cavity of representative CryoRs and DSE rhodopsins.

Another interesting feature of CryoRs is a cavity at the cytoplasmic side found in each interprotomeric cleft and also restricted by the β sheet of the C terminus and the hydrophobic/hydrophilic boundary of the intracellular membrane leaflet (Fig. 5, A and B). The cavity goes through a pore between helices A and G and leads to another cavity inside the protomer (Fig. 5B). The latter cytoplasmic cavity is filled with several water molecules and is surrounded by positively charged conserved amino acid residues, R38, H249, and R57 (CryoR1 numbering), which is the characteristic arginine of CryoRs (Fig. 5, B and C). In the ground state of CryoR1, R57 (T46 in BR) is shielding the E113 (D96 in BR) residue of the functional motif from the cytoplasmic cavity (Fig. 5, B and C).

In contrast to all known MRs, there are no internal water molecules in the central region of CryoR1 and CryoR2 (Fig. 5C and Supplementary Text). No densities in the maps and no space/internal cavities for waters were observed even at the cytoplasmic side of the retinal binding pocket, where normally at least one is found in other MRs near the highly conserved tryptophan residue of the helix F [W207/W208 in CryoR1/CryoR2 and W182 in BR (*46*, *47*)]. We suggest that this is due to the absence of π-bulge in the central part of helix G of CryoRs, which was found in BR and many other MRs and was shown to create additional space for water molecules near the retinal binding pocket. In the RSB region of both CryoR1 and CryoR2 up to the arginine residue at the extracellular side (R99/R100 in CryoR1/CryoR2; analog of R82 in BR), there are also no internal water molecules. One of the reasons for that is a direct interaction of the RSB counterions, leaving no space for water molecules in close proximity to the RSB. In total, no water molecules were found in the radius of ~11 Å from the RSB, which is a unique feature of the studied CryoRs. The absence of the water molecules in the RSB region was also shown for rhodopsin phosphodiesterase (Rh-PDE) from *Salpingoeca rosetta* (*48*). The set of counterions of Rh-PDE is identical to CryoR1 and CryoR2 (E164 and D292 in Rh-PDE). However, in contrast to CryoRs, in Rh-PDE, there is a water molecule at the cytoplasmic side of the retinal binding pocket similar to other MRs. Thus, we suggest that the absence of internal water molecules in the central part of the protein molecule might be a unique feature of CryoRs.

At the extracellular side of CryoRs, we found a large water-filled cavity, between R99/R100 (in CryoR1/CryoR2; analog of R82 in BR) and the small (single turn) N-terminal α helix, capping the internal space of the CryoR protomer from the extracellular bulk (Fig. 5C and Supplementary Text). This helix is similar to that of the light-driven sodium pumps KR2 (*49*, *50*) and *Er*NaR (*51*) and likely other NDQ rhodopsins, but in CryoRs, it is shorter. Both the extracellular cavity and the N-terminal α helix are likely conserved within CryoRs and are found as well in DSE rhodopsins (Fig. 5, D and E). Aside from R99/R100, there are no internal rechargeable residues at the extracellular side of CryoRs. The exception is H230 in CryoR1 (Fig. 5C), which is only found in 2 of 40 members of the CryoRs clade.

### Characteristic arginine flips in the blue-shifted state

**Fig. 6.**
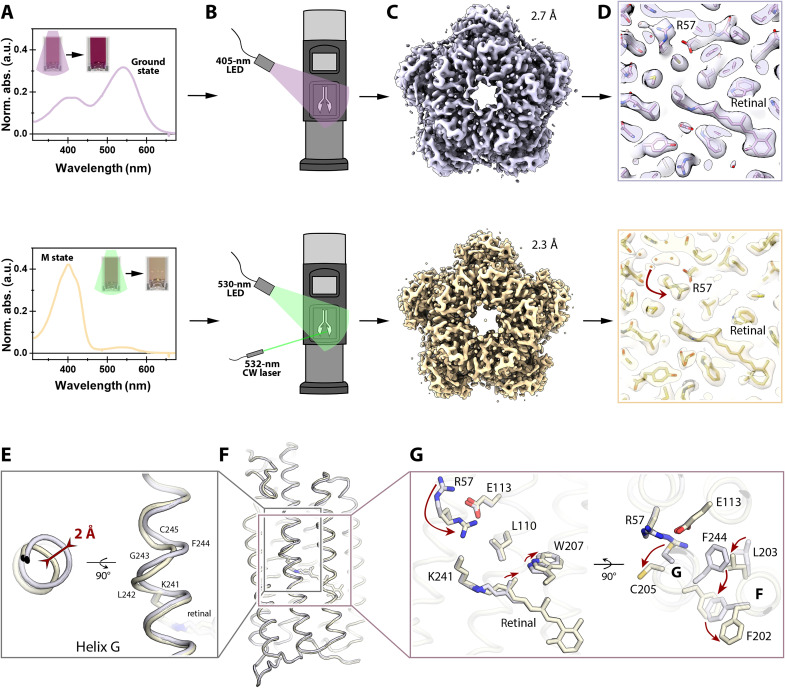
Cryo-EM structures of the ground and M_2_ states of CryoR1. (**A**) Preillumination of the sample solution with 405- and 530-nm LEDs for the ground (top) and M_2_ (bottom) states, respectively. The insets show the sample before and after illumination. Spectra correspond to the illuminated samples. (**B**) Scheme of the cryo-EM grid preparation. For the ground state, the grid was prepared under the 405-nm LED light (top). For the M_2_ state, the grid was prepared under the 530-nm LED and 532-nm continuous wave (cw) laser light (bottom). (**C**) Cryo-EM maps of the ground (top) and M_2_ (bottom) states of CryoR1. (**D**) Cryo-EM maps near the retinal and R57 in the ground (top) and M_2_ (bottom) states. The R57 rearrangement is indicated with a red arrow. (**E**) Distortion of helix G in the M_2_ state (light yellow) compared to the ground state (light purple). Maximum displacement is indicated with a red arrow. (**F**) Overall alignment of the CryoR1 protomer in the ground (light purple) and M_2_ (light yellow) states. (**G**) Detailed view of the rearrangements of R57 and residues in helices F and G between the ground (light purple) and M_2_ (light yellow) states. The major changes are indicated with red arrows.

The spectroscopic analysis shows that CryoR1 can be photoconverted at high pH between the ground and the M_2_ states (Fig. 6A). Accordingly, we prepared a CryoR1 sample at pH 10.5 in both the ground and the M_2_ states using preillumination with 405- and 530-nm LED lamps at room temperature, respectively, for the cryo-EM studies (Fig. 6A). The preilluminated samples were applied to cryo-EM grids and vitrified in liquid ethane. In the case of the ground state of CryoR1, to preserve the purity of the state, the grids were prepared under 405-nm LED lamp illumination (Fig. 6B). The resulting cryo-EM maps at 2.7-Å resolution show the presence of a single state, which we assigned to the ground state (Fig. 6, C and D).

In the case of the accumulated M_2_ state, to maximize the occupancy of the intermediate, the grids were prepared under green light illumination using both a 530-nm LED and a 532-nm laser beam centered onto the grid through the sample application hole on the side of the vitrification robot (Fig. 6B). The resulting cryo-EM maps at 2.3-Å resolution revealed the presence of at least two states of the protein (Fig. 6, C and D). The first is minor and similar to the ground state, while the second is more dominant and clearly different; we assigned it to the M_2_ state.

The cryo-EM map near the retinal and the RSB strongly suggests the 13-cis conformation of the cofactor in the M_2_ state (Fig. 6, D to G). The side chain of W207 is rotated compared to the ground state to allow space for a shifted (0.6 Å) C20 methyl group of the retinal, also indicating the isomerization of the cofactor (Fig. 6G). Thus, since the UV-absorbing M_2_ state is accumulated in CryoR1 under physiological conditions, the mechanism of UV light sensing should be based on isomerization of the retinal from 13-cis to all-trans configuration, which would be unique among MRs.

Following retinal isomerization, the central part of helix G is distorted in the M_2_ state in the region between K241, covalently attached to the retinal cofactor, up to C245 (Fig. 6, E and F). Such disturbance of the central part of helix G is commonly known for other MRs and has been shown structurally for the M states of BR (*47*) and the inward proton pump *Bc*XeR (*52*). In CryoR1, this is accompanied with pronounced flips of the side chains of F244 and C245 (Fig. 6G). The large-scale movement of F244 is accompanied with reorientations of F202 and L203 in the helix F (Fig. 6G). Aside from that, no major conformational changes were found in the helix F, similar to what was shown for the M state of *Bc*XeR (*52*) and in contrast to the ~9-Å shift of the helices E and F occurring in the case of BR (*53*). As there are no constrictions for large-scale rearrangements in the cryo-EM experiments on CryoR1 compared to those known for x-ray crystallography due to crystal contacts (*54*, *55*), we suggest that there is no opening of the cytoplasmic side in CryoRs. The absence of the opening might be rationalized as the E113 residue (analog of proton donor D96 in BR) is accessible from the cytoplasm already in the ground state of CryoRs through the large cavity (Fig. 5B). Moreover, as described further in the manuscript, CryoR1 demonstrated a weak inward proton pumping activity; therefore, the same principle of the mitigation of retinal isomerization by the flipping motion of residues in helix F rather than by major rearrangements of the rhodopsin helices might be similar in CryoRs and xenorhodopsins (*52*).

The above-mentioned structural rearrangements at the cytoplasmic side of CryoR1 are associated with the flip of the unique characteristic R57 residue toward the RSB (Fig. 6G). While in the ground-state R57 points to the cytoplasmic side, in the M_2_ state, it is oriented toward the center of the protomer and is located within ~6.5 Å of the RSB. Notably, in both the ground and M_2_ states, R57 forms a salt bridge with E113 of the functional motif and is additionally stabilized by the main chain of helix G. E113 remains at the same position in the ground and M_2_ states (Fig. 6G).

The x-ray crystallography and cryo-EM structures on CryoR2 also show the flip of the characteristic arginine accompanied by similar rearrangements of the residues in helices F and G (Supplementary Text and figs. S9 and S10). In that case, R58 (analog of R57 in CryoR1) forms a salt bridge with E114 (analog of E113 in CryoR1) in the ground and M_2_ states of CryoR2 in almost identical manner to those shown for CryoR1. Thus, structural data on CryoR1 and CryoR2 allow us to conclude that the reorientation of the characteristic unique arginine at the cytoplasmic side of CryoRs in the long-living blue-shifted state is likely a feature of the entire clade. The reason of the arginine flip remains unclear; however, one can speculate that it is a result of structural rearrangements within the rhodopsin molecule in response to retinal isomerization, propagating via W207, L203, F244, and C245 residues in the case of CryoR1. Another force driving the arginine side chain to move toward the RSB might be in the necessity to compensate for the positive charge in the central part of CryoRs in the blue-shifted state, after the RSB is deprotonated.

### Wavelength- and voltage-dependent proton translocation in CryoR1

Ion-transporting activity tests of the selected proteins by measuring pH changes in the *Escherichia coli* cell suspension upon illumination did not show ion transport at both low and room temperatures (fig. S12). The results remained the same in the presence of protonophore carbonyl cyanide m-chlorophenyl hydrazone (CCCP), indicating the absence of detectable transport activity at least for protons, chloride, and sodium ions (fig. S12). Similar experiments with CryoR1 reconstituted in liposomes demonstrated no light-induced effects on pH at low temperature but showed a possible weak proton translocation at room temperature (fig. S12). It should be noted that the orientation of the CryoR1 when reconstituted into liposomes is stochastic; thus, the deciphering of the direction of proton pumping will require further studies.

**Fig. 7.**
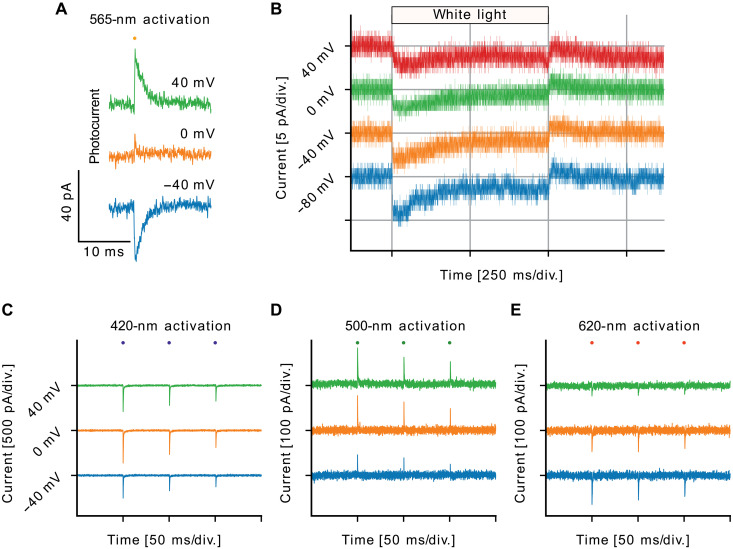
Electrophysiological characterization of CryoR1 in NG108-15 cells. (**A**) Voltage dependence of CryoR1 photocurrents elicited by nanosecond laser pulses with a wavelength of 565 nm. (**B**) Stationary CryoR1 photocurrents upon illumination with white light. (**C** to **E**) CryoR1 photocurrents at the shown voltages elicited by nanosecond laser pulses with wavelengths of (C) 500 nm for the activation of the ground-state subpopulation with the deprotonated counterion complex, (D) 620 nm for the activation of the ground-state subpopulation with the protonated counterion complex, and (E) 420 nm for M_2_ state activation. div., division

Therefore, to get more insights into the proton transport mechanism of CryoRs, we next measured photocurrents in NG108-15 expressing CryoR1 in whole-cell patch-clamp experiments. Since CryoR1 has a broad absorption band at pH 7.4, we first used 565-nm nanosecond light pulses to activate the coexisting subpopulations of the protein. We obtained transient photocurrents, whose vectoriality changed depending on the applied voltage (Fig. 7A).

However, we were unable to elicit stationary photocurrents without additional activation of the long-living M_2_ state, due to the very slow RSB reprotonation kinetics (minutes). The activation of the M_2_ state accelerates the following transition to the ground state (Fig. 2). Hence, we used a white light source, which also included a blue light component, allowing for simultaneous activation of the ground and the M_2_ states (*26*–*29*). As a result, we obtained negative stationary currents during white light illumination at voltages ranging from −80 to 40 mV (Fig. 7B), thereby demonstrating that CryoR1 exhibits an inward proton pumping activity. Although acidic pH accelerates CryoR1’s photocycle, the M_2_ state decay remains slow, taking several seconds even at pH 5.0 and 6.0 (fig. S13). Automated planar patch-clamp experiments with pH exchange confirmed that CryoR1 does not generate stationary photocurrents under 590-nm LED illumination without a blue-light component at any tested pH (fig. S14).

The spectroscopy of CryoR1 has revealed two ground-state subpopulations at pH 7.4 with distinct absorption maxima, one at 550 (likely for the nonprotonated RSB counterion complex) and 620 nm (protonated RSB counterion complex) (Supplementary Text). Thus, to further investigate the mechanism of inward proton pumping and vectoriality change in CryoR1, we separately activated these two ground-state subpopulations and the M_2_ state by using nanosecond pulses of 500, 620, or 420 nm, respectively. The M_2_ state was always activated only after 500- and 620-nm pulses no longer generated measurable photocurrents, so that maximum M_2_ occupancy was ensured before 420-nm activation.

The activation of the ground-state subpopulation with the deprotonated counterion complex using 500-nm pulses led to positive transients (Fig. 7C). The positive transient currents reflect proton transfer toward the extracellular side, likely from the RSB to the counterion complex (fig. S15A).

By contrast, the activation of the ground-state subpopulation with the protonated counterion complex using 620-nm pulses resulted in negative transients at all voltages (Fig. 7D). Such negative transient currents indicate proton transfer to the cytoplasmic side. Thus, when the counterion complex of CryoR1 is protonated, the RSB proton is likely transferred to either E113 or directly the cytoplasmic bulk through the internal cavity (fig. S15B).

The activation of the M_2_ state with 420-nm light pulses resulted in strong negative transient photocurrents at voltages ranging from −40 to +40 mV (Fig. 7E). We integrated the transient photocurrents to quantify the absolute total charge transfer in the transition from M_2_ to the ground state and in the transitions from the two ground-state subpopulations to the M states normalized by their respective population fractions. This analysis revealed that the M_2_–to–ground state transition involves the largest amount of charge transfer (fig. S16), likely through the reprotonation of the RSB, the subsequent partial reprotonation of the counterion complex, as well as the flip of R57 toward the cytoplasmic side observed in the structural data. Notably, as the voltage was changed from −40 to +40 mV, the absolute values of the transient amplitudes increased for 500-nm activation and decreased for 620-nm activation. This, together with the photocurrent reversal at nearly 0 mV upon 565-nm activation, suggests that the distribution between the two ground-state subpopulations is voltage dependent. This observation is also in line with a strong pH dependence of the distribution of the subpopulations. A similar proton transport mechanism was previously described for a PR, which showed photocurrent reversal at acidic conditions (pH 5.5) at a negative membrane potential of −80 mV (*56*).

### Determinants of the dominant blue-shifted state of CryoRs

Our data suggest that the dominant blue-shifted state is a key element in CryoRs, and maximizing its population could be vital for the proper functioning of CryoRs under harsh conditions. The strong blue shift of the spectra in the M_1_ and M_2_ states reflects the deprotonated form of the RSB. Below, we describe the determinants of fast RSB deprotonation and its slow reprotonation throughout the CryoR photocycle.

**Fig. 8.**
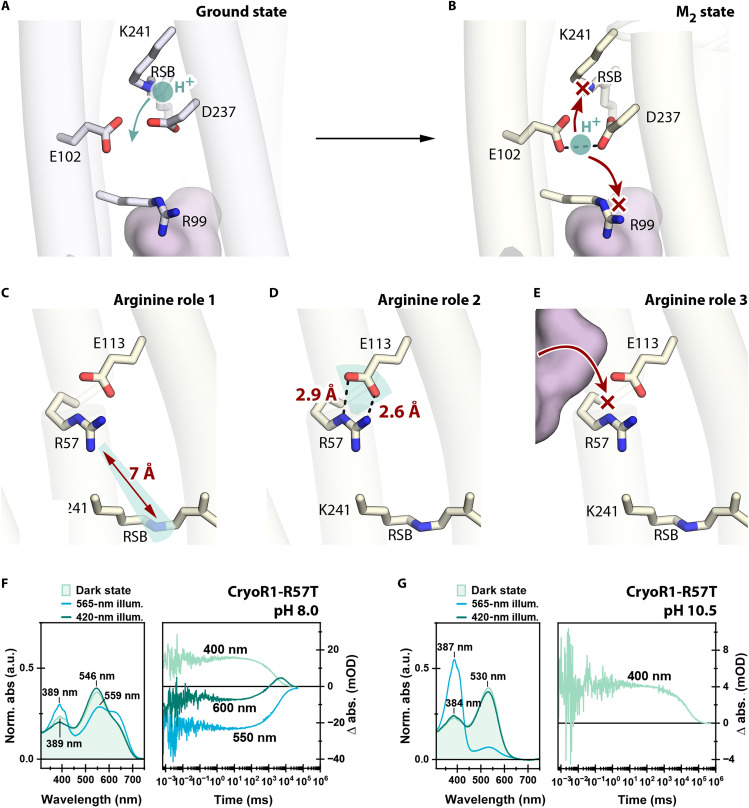
Determinants of the dominant M state in the CryoR photocycle. (**A**) The RSB region in the ground state of CryoR1 at pH 10.5. The proton at the RSB is indicated with a cyan circle. The tentative proton relocation with the rise of the M state is indicated with a cyan arrow. (**B**) The RSB region in the M state of CryoR1 at pH 10.5. The proton relocated to the E102-D237 complex is shown with a cyan circle. The blocked transfer pathways of the proton to the RSB and toward the extracellular side are indicated with red arrows and crosses. (**C**) R57 effect on the p*K*_a_ of the RSB in the M state of CryoR1 (region of the effect highlighted cyan). (**D**) R57 effect on the p*K*_a_ of E113 in the M state of CryoR1 (region of the effect highlighted cyan). (**E**) R57 blocks the pathway for protons from the cytoplasm to the RSB. The cytoplasmic cavity is shown with a pink surface. (**F**) Spectroscopy of CryoR1_R57T_ at pH 8.0. Left: The dark-state spectrum (light green), as well as the PSS spectrum after illumination of the main absorption band (blue) and the spectrum after illumination of the obtained PSS with blue light (dark green) to recover the dark state. Right: The flash photolysis transients at 400, 550, and 600 nm. (**G**) Spectroscopy of CryoR1_R57T_ at pH 10.5. Left: The dark-state spectrum (light green), as well as the PSS spectrum after illumination of the main absorption band (blue) and the spectrum after illumination of the obtained PSS with blue light (dark green) to recover the dark state. Right: The flash photolysis transient at 400 nm. The 565-nm LED was turned on for 100 s (1.05 mW), while the 420-nm LED was turned on for 100 s (0.19 mW).

The deprotonation of the RSB proceeds quickly in all studied CryoRs (Fig. 2). Our structural data strongly suggest that the proton from the RSB is transferred to the complex of two counterions (E102-D237 in CryoR1), which directly interact in the M_2_ state at pH 10.5 (Fig. 8, A and B; fig. S17; and Supplementary Text). It is well known that such pairs of carboxylic residues may have a very high proton affinity, allowing them to effectively store the proton released from the RSB and to hamper its backflow.

At the same moment, the RSB reprotonation is greatly slowed in CryoRs. On the basis of our experimental data, it can be explained as follows. One reason originates from the above-mentioned proton acceptor complex (E102-D237 in CryoR1) and the architecture of the extracellular side of the clade members (Supplementary Text). The proton released from the RSB to its counterions can barely be transferred further as there is an arginine (R99 in CryoR1) barrier and no rechargeable internal groups at the extracellular side of CryoRs (Figs. 5, C and E, and 8B). The region is also isolated from the bulk by the cap formed by the N-terminal α helix (Fig. 5C). However, the presence of the water-filled cavity may provide a weak transport of ions through the region in accordance with the slow photocycle kinetics in a similar way to that recently proposed for an inward proton pump *Bc*XeR (*52*).

Nevertheless, the proton is likely trapped at the counterion complex in the M_2_ state (Fig. 8B). At the same time, our spectroscopy studies of the E102Q, D237N, and E102Q/D237N mutants of CryoR1 as well as the E102Q mutant of CryoR2, imitating various protonation states of the counterion complex, show that once the complex gets protonated, the RSB deprotonates as evidenced by the strong blue shift of the absorption spectra (fig. S8 and Supplementary Text). Therefore, we suggest that the proton, released from the RSB and trapped at the counterion complex in the M_2_ state, stabilizes this long-living intermediate and can only be returned to the Schiff base after slow retinal reisomerization to the original conformation (Fig. 8B). We should note that similar glutamate-aspartate pairs comprising the counterion complex have already been identified in MRs, such as ChRs *Cr*ChR2 (*57*), C1C2 (*58*), and Chrimson (*59*). However, in ChRs, the carboxylic residues do not interact directly but rather via water molecule (fig. S18). Moreover, the extracellular internal region of ChRs is typically very polar and hollow. We suggest that these notable differences between the counterion complexes result in effective proton storage only in the case of CryoRs but not ChRs.

Another reason for the unusually slow RSB reprotonation is associated with the unique arginine residue at the cytoplasmic side (Fig. 8, C to E). Our structural data show that in both CryoR1 and CryoR2, the arginine likely plays a triple role. First, by relocating toward the RSB in the M_2_ state, it lowers the p*K*_a_ (where *K*_a_ is the acid dissociation constant) of the Schiff base, hampering its reprotonation even at neutral and mildly acidic pH values (Fig. 8C). Second, the arginine decreases the p*K*_a_ of a conserved glutamic acid at the cytoplasmic side (E113/E114 in CryoR1/CryoR2) (Fig. 8D). For instance, in PRs, the reprotonation of the RSB proceeds from the internal proton donor (*60*–*62*), which is typically a glutamic acid residue at the cytoplasmic side (E108 in green-light-absorbing PR). This allows an effective RSB reprotonation at neutral and mild-alkaline pH values resulting in outward proton pumping activity of PRs. In CryoRs, the analogous glutamic acid remains deprotonated at pH as low as 4.6 as indicated by the salt bridge formed between R58 and E114 in the crystal structure of CryoR2 (fig. S19 and Supplementary Text). Only at pH 4.3 in CryoR1, the bridge is broken, tentatively indicating the protonation of the glutamic acid (fig. S19). The spectroscopy analysis of the E113Q variant of CryoR1 also showed no effect of the mutation on the photocycle kinetics (fig. S20A), strongly suggesting that the glutamic acid does not serve as a proton donor in CryoRs. Last, the arginine side chain in the M_2_ state blocks the pathway for a proton to the RSB from the cytoplasmic side serving as a positively charged barrier similar to that at the extracellular side (Fig. 8, B and E). We suggest the latter role be the key for the observed inward proton pumping modality of CryoR1, as R57 might prevent the backflow of the proton after its transfer from the RSB toward the cytoplasmic side.

To probe the roles of this arginine in the stabilization of the M_2_ state in CryoRs, we studied the spectroscopic properties of the R57T mutant of CryoR1. In agreement with the proposed role 1, the maximum absorption wavelength of CryoR-R57T differed from that of CryoR1-WT both at pH 8.0 (545 nm versus 550 nm) and 10.5 (534 nm versus 541 nm) already in the ground state (Fig. 8, F and G, and fig. S20B). Furthermore, the difference in the UV peak position between the mutant and WT is more pronounced in the PSS state (389 nm versus 400 nm at pH 8.0 and 388 nm versus 400 nm at pH 10.5), in line with a shorter distance (6.5 Å) between R57 and the RSB in the intermediate (Fig. 8C and fig. S20B). Next, at pH 8.0, the decay of the blue-shifted state in the mutant is ~100 times faster than in the CryoR1-WT (Fig. 8F and fig. S20B). This coincides with the pronounced formation of the red-shifted late intermediate (Fig. 8F). At pH 10.5, the behavior is similar to that of WT (Fig. 8G). Thus, the data strongly suggest that the unique arginine of CryoRs stabilizes the blue-shifted state providing for its unusually long lifetime at neutral and mild-alkaline pH. At alkaline pH, the photocycle kinetics is also extremely slow but seems independent of the arginine. Such behavior is expected since the concentration of protons is much lower, which, typically for MRs, results in a notably decelerated photocycle.

### Genomic context supports a sensory role of CryoRs

**Fig. 9.**
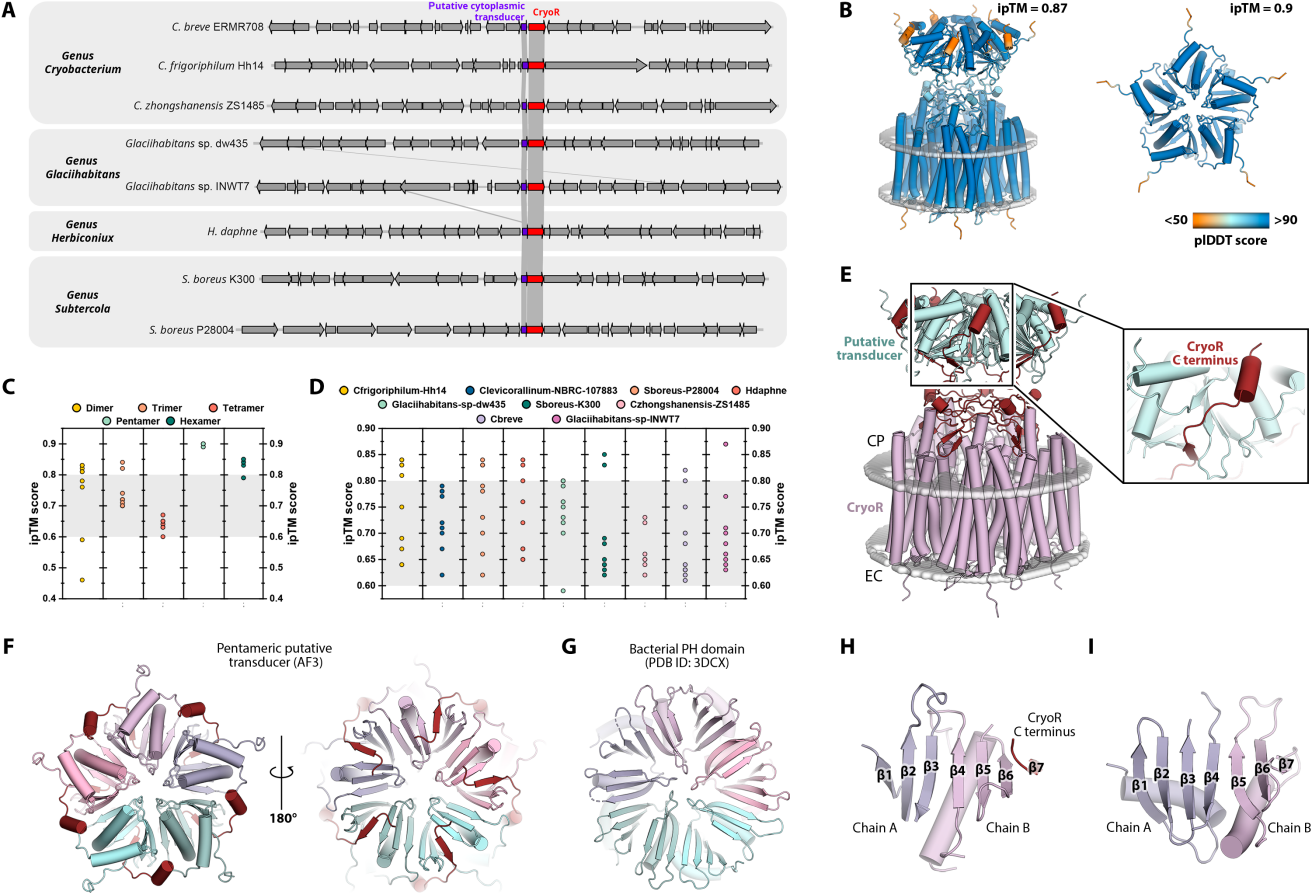
Putative cytoplasmic transducers of CryoRs. (**A**) Alignment of contigs containing the CryoR gene in bacteria available in GenBank. The accompanying putatively cotranscribed CDS is colored in blue. For *Herbiconiux daphne *(*H. daphne*) strain information was not provided. (**B**) AF3 prediction of the CryoR1/putative transducer complex (left) and pentamer of putative transducer alone (right) from *Glacihabitans* sp. INWT7. Models are colored according to predicted local distance difference test (plDDT) score. (**C**) ipTM scores for predictions of different oligomeric states of putative transducer from *Glacihabitans* sp. INWT7. Prediction of pentameric assembly has the highest ipTM score of 0.9. (**D**) ipTM scores of the structures of CryoR/putative transducer complexes from (A). Gray area indicates the ipTM region of 0.6 to 0.8, where the solutions could be correct or wrong. For each complex, 10 predictions with different seeds have been calculated. (**E**) Detailed view of the C terminus of CryoR (dark red) interaction with the putative transducer (cyan). (**F**) View on the predicted putative transducer from the cytoplasmic side (left) and rotated by 180° (right). (**G**) Similar β strands arrangements in the pentamer of bacterial pleckstrin homology (PH) domain (*64*) [Protein Data Bank (PDB) ID: 3DCX]. (**H**) β Sheet (β blade) of the putative transducer formed by two adjacent protomers (purple and pink) and C terminus of CryoR (dark red). (**I**) β Sheet (β blade) of the bacterial PH domain (PDB ID: 3DCX) formed by two adjacent protomers (purple and pink).

We analyzed all the genomes containing CryoR genes available (table S1) to determine whether any specific coding DNA sequences (CDSs) were frequently associated with the CryoR gene since genomic context is often helpful in determining the biological role. In all genomes, a CDS appeared next, or even overlapping, with the CryoR (Fig. 9). This strongly indicates their cotranscription and related activity. We have named these CDSs as putative CryoRs cytoplasmic transducers. The transducers are relatively small water-soluble proteins, which are hard to annotate. To gain more insights in possible interactions, we used AlphaFold3 (AF3) (*63*) to predict the structure of the putative transducer and CryoR-transducer complex (Fig. 9B). First, we probed predictions of different homooligomers (2 to 7) of the putative transducers (Fig. 9C). For each complex, we ran 10 predictions with different seeds to improve statistics. They showed that putative transducers most likely form pentamers, according to the maximal predicted template modeling and the interface predicted template modeling (ipTM) scores for their pentameric complexes (Fig. 9C). The predicted dimers, trimers, and tetramers represented parts of a suggested pentamer. Next, we used AF3 to predict the CryoR-transducer complexes of eight pairs of genes available from GenBank shown in Fig. 9A. For each complex, we again ran 10 predictions with different seeds to improve statistics. The resulting complexes with ipTM > 0.8, indicating a reliable solution, demonstrate similar arrangement with the transducer interacting with the C terminus of CryoR at the cytoplasmic side (Fig. 9, B and E). According to the scores, the most accurate prediction was made for the CryoR/putative transducer complex from *Glacihabitans* sp. INWT7, depicted in Fig. 9. The tentative interaction of putative transducers and CryoRs might explain the conservation of the unusual C terminal elongation within CryoRs. Noteworthy, the structure prediction of the transducers also showed that their C termini are likely disordered, which might be interacting with other protein(s) in a signal cascade (Fig. 9).

The organization of putative transducers in pentamers results in the arrangements of six β strands of the two adjacent protomers together with a short β strand in the C terminus of CryoR into a seven-stranded β sheet. These β sheets are organized similar to β blades found in pentameric bacterial pleckstrin homology (PH) domains (Fig. 9, G to I) (*64*). Thus, the architecture of the putative transducer is reminiscent of the bacterial PH domains forming ring complexes using the same kind of β sheet augmentation mechanism. PH domains were shown to interact with cell membranes and are involved in various processes, such as intracellular signaling and cytoskeletal organization, and, in some cases, trafficking other proteins to certain cell compartments. The latter might mean that putative transducers of CryoRs also function in close proximity to membranes being anchored to the rhodopsins and might work as adaptors for further signal propagation. To enable further research, we deposited a new family that includes these proteins in Pfam. It has been called DUF7882 and assigned accession number PF25355.

## DISCUSSION

Here, we characterized a group of MRs, CryoRs, members of which were found in cold-living bacteria. CryoRs demonstrate common features such as a minutes-long photocycle dominated by a blue-shifted intermediate state and the presence of an arginine residue at the cytoplasmic side near the position of the proton donor in many archaeal, bacterial, and eukaryotic proton pumping rhodopsins.

Our data suggest that CryoRs exhibit inward proton pumping activity. Notably, this requires illumination by white light sources containing a UV component to accelerate Schiff base reprotonation as shown for CryoR1. Because of the requirement of the UV component for substantial proton pumping activity, we consider CryoRs could play a role in sensing potentially harmful UV light. Moreover, we found a putative cytoplasmic transducer of CryoRs that is likely cotranscribed with the rhodopsin, suggesting that together they can trigger signal cascades in response to UV irradiation via protein-protein interactions. Future work will be required to study putative transducers.

The characteristic arginine in the helix B is the key determinant allowing CryoRs to markedly increase the lifetime of the blue-shifted state. Nevertheless, the overall structure of CryoRs and unusual sets of counterions also contribute largely to its stabilization. The molecular mechanism of the stabilization of the blue-shifted state involves the unusual flipping motion of the arginine residue to serve as a barrier for proton translocation back to the RSB.

The intriguing ability of retinal proteins to form a photochromic pair—comprising two forms with distinct absorption maxima and reversible conversion through photonic stimuli—has captivated scientists for a long time. This fascination stems from the potential application of these proteins in recording, processing, and storing optical information, giving rise to numerous proposed uses such as three-dimensional (3D) optical memories, real-time holographic processors, and artificial retinas. While BR has historically been the most used protein for these purposes, its most advanced development has been in the field of information storage, driven by numerous studies [see (*65*) for review], supported by multiple patents (*66*–*69*), and even leading to the development of prototype products (*70*). Most applications relied on the M state photochromic shift of BR, as it generates the most spectrally distinct state from the ground state, allowing the assignment of the ground and M states as 0 and 1 for data storage. However, the principal drawback is the short lifetime of the M state, approximately 10 ms, limiting its use to transient optical data storage. Various strategies have been used to address this limitation, including chemical modification of retinal, site-specific mutagenesis, chemical supplements, or the utilization of branched P/Q states—each accompanied by its unique drawbacks. Retinal chemical modifications hinder protein production, the best mutants have been able to prolong the M state only to the scale of seconds (*71**–**73*), and the P/Q states demonstrate extremely low quantum efficiency—500 times lower than normal activation—making it notably more challenging to photoconvert the protein (*69*). In this context, CryoRs, which can be easily expressed in *E. coli*, naturally exhibit an M state lasting several minutes and demonstrate efficient photoconversion, making them a promising template for future advancements in this field.

The ability of CryoR1 to elicit opposing transient photocurrents in response to ground-state activation with red (620 nm) and green (500 nm) light pulses suggests potential utility of CryoRs for bidirectional, dual-color optogenetic control of cells, which now involves the use of tandem protein constructs (*74*, *75*). However, the partial charge transfers in CryoR1 upon illumination with either red or green light pulses are likely too small for optogenetic activation or silencing of excitable cells. Nevertheless, CryoR variants, which exhibit larger partial charge transfer or are able to transfer proton toward extracellular bulk upon 500-nm light illumination and demonstrate strong plasma membrane–targeted expression, might allow in the future bidirectional optogenetic control with only one heptahelical protein.

Last, the variation of functional motifs within the CryoR clade, in particular, the alteration of amino acid residues close to the retinal cofactor, results in notably different absorption spectra of the studied proteins. Taking into account the extreme and harsh environmental conditions of cold-living organisms like *Cryobacteria* and difficulty of their cultivation, our work provides a solid basis for further investigations of CryoRs and also sheds light onto the unusual light-sensing in psychrotrophic bacteria.

## MATERIALS AND METHODS

### Search for CryoRs, phylogenetic analysis, and genomic context

Initial search for the rhodopsins was performed against the UniProtKB (2023-09-15), UniParc (2023-09-15), GenBank (2023-09-15), and MGnify (2023-09-15) databases using hmmsearch (hmmsearch --cut_ga -A <output_alignment.mul> <MRs_hmm_profile.hmm> <gene_database.tar.gz>) with the HMM profile of the MRs family from Pfam (PF01036). The seven-letter motifs were retrieved using custom Python scripts using jupyter notebook. Among these motifs, unusual RXXXXXK motifs were found using manual inspection. The sequences having the RXXXXXK motifs were shown to cluster together and originate from bacteria isolated from cold environments. To find more of such sequences, the final search was performed using jackhmmer (*N* iterations = 5) using the sequence of CryoR1 rhodopsin (GenBank ID: WP_166787544, found in the course of the initial search and had the RXXXXXK motif) as a template. All sequences having the RXXXXXK motif were selected, filtered to contain more than 250 amino acid residues, and realigned separately using multiple sequence comparison by log-expectation (MUSCLE) (*76*). Duplicated sequences were removed using a custom Python script. The selected sequences were also manually inspected to contain full seven transmembrane α helices. The maximum likelihood phylogenetic tree was built using IQ-TREE (*77*) (--perturb 0.2 --nstop 500 -B 1000 -m TEST --alrt 1000 -T 12) and visualized using iTOL (v6.8) (*78*). For the tree building, we used 2199 sequences of MRs from (*1*). The sequences of CryoRs and three sequences of the DSE rhodopsins reported in (*17*) were added to the 2199 sequences and aligned using multiple alignment using fast Fourier transform (MAFFT) (--auto) (*79*). For the building of the phylogenetic tree, all sequences with identity higher than 90% were removed using the CD-HIT software (*80*). The full multiple sequence alignment (MSA) file, 90% identity-filtered MSA file, and phylogenetic tree file are provided as supplementary files.

To determine the genes near the CryoR, nucleotide sequences from genomes containing the CryoR gene and described in table S1 were downloaded from GenBank. Prodigal 2.6.3 (*81*) was used to predict the CDSs from contigs retrieved from individual isolate genomes. Predicted protein-encoded genes were functionally annotated against the National Center for Biotechnology Information nonredundant (NR) database using DIAMOND 0.9.15 (*82*) and against COG (*83*) and TIGRFAM (*84*) using HMMscan 3.3 (*85*). Domains within proteins were predicted using InterPro (*86*). Transmembrane domains and signal peptides were identified with DeepTMHMM (*87*) and SignalP v6.0 (*88*), respectively, to detect the predicted proteins’ location. Genomic fragments of ~50 kb long containing the CryoR near the fragment’s center were extracted from the annotated genomes to evaluate the genomic context near this gene and the presence or absence of synteny among genomes. Genomes on which the CryoR was found near the contig ends were not considered for the analysis. Sequences were compared using blastp (*85*), with a minimum amino acid identity of 30% and a minimum protein alignment of 50%.

### Cloning

The DNA coding CryoR1-5 was optimized for *E. coli* using GeneArt (Thermo Fisher Scientific). Genes were synthesized commercially (Eurofins). For protein expression and purification, the pET15b plasmid with 6xHis-tag at the C terminus was used. Point mutations and C terminus deletions were introduced using whole-plasmid polymerase chain reaction followed by the blunt ends ligation.

For electrophysiological recordings, a human codon-optimized gene of CryoR1 was cloned into the pcDNA3.1(−) vector between Bam HI and Hind III sites together with an N-terminal part of ChRs (C2C1), membrane trafficking signal (TS), and endoplasmatic reticulum (ER) export signal (ES) from potassium channel Kir2.1 and enhanced yellow fluorescent protein (EYFP). C2C1 and TS-EYFP-ES were amplified from pEYFP-N1-eKR2 (*89*), which was a gift from P. Hegemann (Addgene plasmid #115337).

### Protein expression, solubilization, and purification

*E. coli* cells were transformed with pET15b plasmid containing the gene of interest. Transformed cells were grown at 37°C in shaking baffled flasks in an autoinducing medium ZYP-5052 (*90*), containing ampicillin (10 mg/liter). They were induced at an OD_600_ (optical density at 600 nm) of 0.8 to 0.9 with 1 mM isopropyl-β-d-thiogalactopyranoside. Subsequently, 10 μM all-trans-retinal was added. Incubation continued for 3 hours. The cells were collected by centrifugation at 4000*g* for 25 min. Collected cells were disrupted in an M-110P Lab Homogenizer (Microfluidics) at 172.369 MPa in a buffer containing 20 mM tris-HCl (pH 8.0), 5% glycerol, 0.5% Triton X-100 (Sigma-Aldrich), and deoxyribonuclease I (50 mg/liter; Sigma-Aldrich). The membrane fraction of the cell lysate was isolated by ultracentrifugation at 125,000*g* for 1 hour at 4°C. The pellet was resuspended in a buffer containing 20 mM tris-HCl (pH 8.0), 0.2 M NaCl, and 1% n-Dodecyl-β-D-maltoside (DDM; Anatrace, Affymetrix) and stirred overnight for solubilization. The insoluble fraction was removed by ultracentrifugation at 125,000*g* for 1 hour at 4°C. The supernatant was loaded on a nickel-nitrilotriacetic acid (Ni-NTA) column (QIAGEN), and the protein was eluted in a buffer containing 20 mM tris-HCl (pH 8.0), 0.2 M NaCl, 0.4 M imidazole, and 0.1% DDM. The eluate was subjected to size exclusion chromatography on a Superdex 200i 300/10 (GE Healthcare Life Sciences) in a buffer containing 20 mM tris-HCl (pH 8.0), 100 mM NaCl, and 0.03% DDM. In the end, protein was concentrated to 70 mg/ml for crystallization and −80°C storage.

### Measurements of pump activity in the *E. coli* cell suspension

CryoRs were expressed as described above. The cells were collected by centrifugation at 4000*g* for 15 min and were washed three times with an unbuffered 100 mM NaCl solution, with 30-min intervals between the washes to allow for exchange of the ions inside the cells with the bulk. After that, the cells were resuspended in an unbuffered 100 mM NaCl solution and adjusted to an OD_600_ of 8.5. The measurements were performed in 3-ml aliquots of stirred cell suspension kept at ice-cold temperature (0.3° to 1.0°C). For measurements at higher temperature, the cell suspension was kept at room temperature (20° to 22°C). The cells were illuminated using a halogen lamp, and the light-induced pH changes were monitored with a pH meter (Mettler Toledo). The measurements were repeated under the same conditions after the addition of 30 μM CCCP protonophore. For the measurements of the pH changes at low pH values, the pH of the final cell suspension was adjusted using 10% HCl solution.

### Proteoliposome preparation and pump activity measurements in liposome suspension

Phospholipids (asolectin from soybean, Merck) were dissolved in CHCl_3_ (chloroform ultrapure, PanReac AppliChem) and dried under a stream of N_2_ in a glass vial. Residual solvent was removed using a vacuum pump overnight. The dried lipids were resuspended at a final concentration of 1% (w/v) in 0.15 M NaCl supplemented with 2% (w/v) sodium cholate. The mixture was clarified by sonication at 4°C, and solubilized protein (light-driven sodium pump KR2 as a reference or CryoR1) was added at a protein/lipid ratio of 7:100 (w/w). The detergent was removed by overnight stirring with detergent-absorbing beads (Amberlite XAD-2, Supelco). The mixture was dialyzed against 0.15 M NaCl (adjusted to a desired pH) at 4°C for 1 day (four 200-ml changes) to obtain a certain pH.

The pH change upon white light illumination measurements was performed on 2 ml of stirred proteoliposome suspension at 0°C and at room temperature (20° to 22°C). Proteoliposomes were illuminated for 3 min using a halogen lamp (Intralux 5000-1, Volpi) and then were kept in the dark for another 3 min. Changes in pH were monitored using a pH meter (Mettler Toledo). Measurements were repeated for different starting pH and in the presence of 30 μM CCCP under the same conditions.

### Cell culture and transfection

The patch-clamp recordings of CryoR1 were conducted in the neuroma glioblastoma cell line NG108-15 (American Type Culture Collection, HB-12377TM, Manassas, USA) cultured in Dulbecco’s modified Eagle’s medium (DMEM; Sigma-Aldrich, St. Louis, USA) supplemented with 10% fetal calf serum (Sigma-Aldrich, St. Louis, USA) and 1% penicillin/streptomycin (Sigma-Aldrich, St. Louis, USA) (supplemented DMEM:DMEM+) at 37°C and 5% CO_2_. Cells were seeded on 24-well plates 1 day before transfection by Lipofectamine with pcDNA3.1(−) derivatives carrying the CryoR1 gene at a NG108-15 cell confluency of 50 to 70%. For each well, a transfection mix of 100 μl of DMEM, 2 μl of Lipofectamine LTX (Invitrogen, Carlsbad, USA), and 500 ng of the plasmid DNA was prepared and added to a well with 400 μl of DMEM+. Twenty-four hours after transfection, the medium was exchanged against 500 μl DMEM+ supplemented with 1 μM all-trans retinal.

For automated planar patch-clamp experiments, NG108-15 cells were seeded in six-well plates at a density of 150,000 cells per well. The following day, the cells were transfected with plasmids carrying genes CryoR1 or *Ns*XeR (*24*) using the polyethyleneimine (PEI; Polysciences, Warrington, PA, USA) transfection reagent. For each well, 2.5 μg of plasmid DNA and 7.5 μg of PEI were separately dissolved in 0.2 ml of Opti-MEM (Thermo Fisher Scientific, Waltham, MA, USA). The DNA and PEI solutions were then combined and incubated for 15 min at room temperature. The resulting DNA/PEI mixture was added to the wells and incubated at 37°C with 5% CO_2_ for 16 hours. After transfection, the medium was replaced with fresh DMEM+ supplemented with 1 μM all-trans retinal, and the cells were incubated for an additional 28 hours at 37°C with 5% CO_2_ before the automated patch-clamp experiments.

### Manual patch-clamp recordings and data analysis

The electrophysiological characterization of CryoR1 was conducted by whole-cell patch-clamp recordings of transiently transfected NG108-15 cells. Cells were patched 2 days after transfection under voltage-clamp conditions using the Axopatch 200B amplifier (Axon Instruments, Union City, USA) and the DigiData1440A interface (Axon Instruments, Union City, USA). Patch pipettes with a resistance of 2 to 6 megohm were fabricated from thin-walled borosilicate glass on a horizontal puller (model P-1000, Sutter Instruments, Novato, USA). The series resistance was <15 megohm. The bath solution contained 140 mM NaCl, 2 mM CaCl2, 2 mM MgCl_2_, and 10 mM Hepes (pH 7.4); and the pipette solution contained 110 mM NaCl, 2 mM MgCl_2_, 10 mM EGTA, and 10 mM Hepes (pH 7.4). All recordings were performed at room temperature (20°C).

White light pulses were applied by a fast computer-controlled shutter (Uniblitz LS6ZM2, Vincent Associates, Rochester, USA) using Olympus U-HGLGPS 130W illumination system focused into an optic fiber (*d* = 400 μm). The stationary photocurrents of the CryoR1 were measured in response to white light pulses with an intensity of 14 mW/mm^2^. The nanosecond light pulses of wavelengths ranging from 420 to 620 nm were applied using the Opolette 355 tunable laser system (Opotek Inc., Carlsbad, USA) coupled into an optic fiber (*d* = 400 μm). To activate the ground state, CryoR1 was preconditioned with 420-nm light pulses to ensure it was in the desired state. For the M_2_ state activation, prior stimulation was provided by 565-nm light pulses. This preparatory illumination, whether with 420- or 565-nm pulses, was continued until the protein no longer generated transient currents at a membrane potential of −40 mV.

The custom Python scripts developed in-house were used for data analysis. To calculate the absolute total charges transferred, we integrated the photocurrent over time for all sequential pulses until the photocurrents ceased. To determine the fractions of CryoR1 proteins in the two subpopulations, we compared the charge transfer in the first pulses recorded on the same cell at two different voltages. The ratio of charges transferred when activated with a 500-nm (620 nm) laser pulse at two different voltages is proportional to the fraction of the protein in the nonprotonated (protonated) counterion subpopulation.

Illuminating with 420-nm light pulses returns all proteins to the ground state, implying that the total fractions (denoted as *p*) in the two subpopulations sum to 1 across all voltages

$$p_{\text{non}-\text{prot},0\;\text{mV}}+p_{\text{prot},0\;\text{mV}}=1$$

$$p_{\text{non}-\text{prot},-40\;\text{mV}}+p_{\text{prot},-40\;\text{mV}}=1$$

Assuming the total number of proteins remains constant during the experiment, the charge transferred (denoted as *Q*) after illumination with the first light pulse of a specified color is directly proportional to the fraction of proteins in the respective subpopulation

$$\frac{Q_{500\;\text{nm},0\;\text{mV}}}{Q_{500\;\text{nm},-40\;\text{mV}}}=\alpha =\frac{p_{\text{non}-\text{prot},0\;\text{mV}}}{p_{\text{non}-\text{prot},-40\;\text{mV}}}$$

$$\frac{Q_{620\;\text{nm},0\;\text{mV}}}{Q_{620\;\text{nm},-40\;\text{mV}}}=\beta =\frac{p_{\text{prot},0\;\text{mV}}}{p_{\text{prot},-40\;\text{mV}}}=\frac{1-p_{\text{non}-\text{prot},0\;\text{mV}}}{1-p_{\text{non}-\text{prot},-40\;\text{mV}}}$$

When combined, this allows us to derive the formula for the fraction of proteins in one of the subpopulations, which we used for our estimations

$$p_{\text{non}-\text{prot},0\;\text{mV}}=\frac{\alpha \;\left(1-\beta \right)}{\alpha -\beta }$$

### Automated planar patch-clamp recordings

Automated patch-clamp recordings were performed using a Syncropatch 384 system (Nanion, Munich, Germany) equipped with prototype illumination units. The Syncropatch 384 was operated with Biomek v2.1 software, and data were acquired using PatchControl v2.2 software. Transfected human embryonic kidney 293T cells were washed twice with Dulbecco's Balanced Salt Solution (DPBS; Thermo Fisher Scientific, Waltham, MA, USA) and treated with 0.2 ml of TrypLE (Thermo Fisher Scientific) per well at 37°C for 15 min. Following TrypLE treatment, 2 ml of standard external solution per well was added, and cells were incubated at 4°C for 5 min. The standard external solution contained 140 mM NaCl, 4 mM KCl, 2 mM CaCl_2_, 1 mM MgCl_2_, and 10 mM Hepes, with the pH adjusted to 7.4 using NaOH and osmolarity set to 298 mOsm by adjusting the glucose concentration. The cells were then resuspended by pipetting and added to 384-well S-type chips (Nanion). A gigaseal was established through a sequence of applied pressure steps and was enhanced by increasing the external calcium concentration to 6 mM, combined with a fluoride-based internal solution. Whole-cell patch-clamp configuration was achieved using three suction pulses of −350 mbar for 5 s each.

The internal solution consisted of 10 mM NaCl, 10 mM KCl, 110 mM KF, 10 mM Hepes, and 10 mM EGTA, with the pH adjusted to 7.2 using KOH. The extracellular solution was identical to the standard solution described above. During the experiments, the intracellular solution was sequentially exchanged after each recording. After the first recording, it was replaced with a solution containing 10 mM NaCl, 10 mM KCl, 110 mM KF, 10 mM MES, and 10 mM EGTA, with the pH adjusted to 6.0 using KOH. After the second recording, it was further exchanged with a solution containing 10 mM NaCl, 10 mM KCl, 110 mM KF, 10 mM MES, and 10 mM EGTA, with the pH adjusted to 5.0 using citric acid. Correspondingly, the extracellular pH was adjusted by a series of dilutions (total dilution factor of 60) to replace the solution with 140 mM NaCl, 4 mM KCl, 2 mM CaCl_2_, 1 mM MgCl_2_, and 10 mM MES at pH 6.0 and then 140 mM NaCl, 4 mM KCl, 2 mM CaCl_2_, 1 mM MgCl_2_, and 10 mM MES at pH 5.0.

To illuminate the cells during the planar patch-clamp experiment, a specialized illumination unit was used, consisting of 96 LEDs (Lumileds Luxeon Z, Schiphol, Haarlemmermeer, The Netherlands) coupled to light fibers. During the experiment, the fiber tip was submerged in the solution and positioned 7 mm from the cell being measured. To activate CryoR1 and *Ns*XeR, 500-ms pulses from orange LEDs (λ_max_ = 590 nm, LXZ1-PL02) at an intensity of 5 mW/mm^2^ were applied at a membrane potential of −60 mV. After each orange light pulse, 500-ms pulses from UV LEDs (λ_max_ = 385 nm, LHUV-A040) at an intensity of 1.5 mW/mm^2^ were used to return the proteins to their ground states.

### Nanodisc reconstitution

For the dataset CryoR1 pH 8.0 in nanodisc, protein reconstitution was performed as previously described (*91*). Namely, liposomes (20 mg/ml), containing *E. coli* polar lipids and egg phosphatidylcholine (PC; w/w 3:1, Avanti), were solubilized by adding 30 mM DDM followed by 1-min vortexing and 3-hour incubation at 4°C. Thawed purified rhodopsin, cleaved MSP2N2, and solubilized lipids were mixed in a 3:5:100 molar ratio (considering a single protein chain) and incubated at 4°C for 90 min while nutating. To remove the detergent, BioBeads (Bio-Rad) were added, and the mixture was incubated overnight (10 hours) at 4°C. After removing BioBeads with a syringe, the solution was supplied with 15 mM imidazole (pH 8.0) and Ni-NTA resin, equilibrated with 50 mM tris-HCl (pH 8.0), and 100 mM NaCl to remove empty nanodiscs. After 1-hour incubation at 4°C on a rocking platform, resin was washed with 50 mM tris-HCl (pH 8.0), 300 mM NaCl, and 30 mM imidazole (pH 8.0), and nanodiscs were eluted with 500 mM imidazole in the same buffer. The sample was centrifuged (10 min, 20,000*g*, 4°C) and applied to Superdex 200 10/300 gel-filtration column (GE Healthcare) equilibrated with 20 mM tris-HCl (pH 8.0) and 100 mM NaCl. Fractions with nanodiscs were concentrated using VivaSpin 500 molecular weight cut-off (MWCO) 100-kDa concentrators (Sartorius) to the target concentration (around 2 mg/ml).

### Steady-state absorption spectroscopy, pH titration, and illumination experiments

Absorption spectra of CryoR samples were measured with an absorption spectrometer (Specord600, Analytik Jena). Before and after each experiment, absorption spectra were taken to check the sample quality.

For the pH titration, equal amounts of the protein stock solution (3.3 μl) were added to 200 μl of the respective buffer to account for the same protein concentration in all prepared samples. The protein was suspended in the titration buffer containing 10 mM trisodium citrate, 10 mM MES, 10 mM Hepes, 10 mM tris, 10 mM *N*-cyclohexyl-2-aminoethanesulfonic acid (CHES), 10 mM 3-(cyclohexylamino)propane-1-sulfonic acid (CAPS), and 10 mM Arg-HCl. The pH was adjusted with tiny amounts of HCl (5000 mM) or NaOH (5000 mM), respectively.

For illumination experiments, solutions of each sample were prepared to have an OD of ~0.5 at an optical pathway of 10 mm. First of all, the dark-state spectrum was measured; second, the main absorption band was illuminated using LEDs that match the absorption spectrum; and in the final step, the arised M-like state was illuminated. The following LEDs and LED settings were used: Thorlabs M420L3 (0.19 mW, 100 s), Thorlabs M530L (0.84 mW, 100 s), Thorlabs M565L2 (1.05 mW, 100 s), Thorlabs M590L3 (0.26 mW, 100 s), and Thorlabs M625L2 (6.4 mW, 100 s). LED output powers were measured using an Optometer P9710 (Gigahertz Optik) with a BN-DSR-100F-2 detector at a distance of ~3.5 cm.

Experiments at pH 3.5 were conducted in a 20 mM sodium citrate buffer additionally consisting of 200 mM NaCl and 0.05% DDM. At pH 8.0, a buffer consisting of 20 mM tris, 200 mM NaCl, and 0.05% DDM was prepared. At pH 10.5, a 20 mM CAPS buffer with 200 mM NaCl and 0.05% DDM was used.

To determine the lifetimes of the PSS decay accompanied with the recovery of the parent state, the PSS was formed applying similar illumination procedures as described above (Thorlabs M565L2, 565 nm, 1.05 mW, 100 s). Absorption spectra were recorded every 120 s, while the first spectrum was taken before illumination. The measurement duration was set to 5 hours for both 5° and 20°C. Before further analysis, a background correction was performed for each spectrum, and 100 s was subtracted from the time points, to correct for the 100-s illumination period in between the measurement of the first and the second spectra.

To determine the effects of the sunlight exposure on the CryoR1 dark-state spectrum, a sample at pH 8.0 was exposed to sunlight outside the building in a cuvette for 30 min. Afterward, spectra were measured every 30 s for 1 hour in total. Forty-five seconds elapsed in between removing the sample from sunlight and putting it into the spectrometer to start the measurement. In addition, the effect of the overhead light in the laboratory was investigated with a time series of absorption spectra of 30 min in total. Spectra were measured every 30 s, while the overhead light was turned on in between the first and the second measurements.

### Ultrafast transient absorption spectroscopy

Ultrafast transient absorption measurements were performed with a home-built pump-probe setup as described previously (*92*). A Ti:Sa chirped pulse regenerative amplifier (MXR-CPA-iSeries, Clark-MXR Inc.) served as the fs-laser source and was operated at a central wavelength of 775 nm and with a repetition rate of 1 kHz, resulting in laser pulses with a pulse width of ~150 fs. For excitation, the pulses from a two-stage noncollinear optical parametric amplifier (NOPA) setup were spectrally adjusted according to the absorption spectrum of the sample (600 and 620 nm for measurements at pH 3.5 and 560 nm for measurements at pH 10.5). For the probing of the photoinduced changes of sample absorbance, the laser fundamental was focused into a 5-mm CaF_2_ crystal to generate supercontinuum pulses. The generated white light was then split up and guided through sample and reference pathways, respectively. Two identical spectrographs (Multimode, AMKO), equipped with a grating (500-nm blaze, 1200 grooves per mm), a photodiode array (S8865-64, Hamamatsu Photonics), and a driver circuit (C9918, Hamamatsu Photonics) were used for signal detection. The obtained signals were digitized via a 16-bit data acquisition card (NI-PCI-6110, National Instruments). The pump and probe pulses were set to the magic angle (54.7°) configuration to eliminate anisotropic effects. Furthermore, the sample was constantly moved in a plane perpendicular to the excitation beam to avoid multiple excitation and sample degradation.

The samples were prepared to contain a protein concentration equal to an optical density of ~0.3 for all measurements of CryoR1 at pH 3.5, ~0.2 OD for the measurement of CryoR1 at pH 10.5, and ~0.15 OD for the measurements of CryoR2 at both pH 3.5 and pH 10.5. Because of their slow photocycle kinetics at pH 10.5, respective samples were constantly illuminated using a Thorlabs M405L4 LED (8.71 mW).

### Transient flash photolysis spectroscopy

A Nd:YAG laser (SpitLight 600, Innolas Laser) was used to pump an optical parametric oscillator (OPO; preciScan, GWU-Lasertechnik). The OPO was set to generate excitation pulses according to the absorption maximum of the respective sample at an average pulse energy of ~2.2 mJ/cm^2^. A Xenon or a Mercury-Xenon lamp (LC-8, Hamamatsu) served as probe light sources. Two identical monochromators (1200 liters/mm, 500-nm blaze), one in front and one after the sample, set the chosen probing wavelengths. Absorption changes were detected by a photomultiplier tube (Photosensor H6780-02, Hamamatsu) and then converted into an electrical signal, which was recorded by two oscilloscopes (PicoScope 5244B/D, Pico Technology) with overlapping timescales. For each transient, 30 acquisitions were measured and averaged to increase the signal-to-noise ratio. To obtain data files with a reasonable size for further analysis, raw data files were reduced using forward averaging and a combined linear and logarithmic timescale.

Detergent-solubilized samples were measured in a 2 × 10 mm quartz cuvette and prepared to have a protein concentration equal to an optical density of ~1.0 at an optical path length of 10 mm. For the measurement presented in Fig. 3B, a Thorlabs M405L4 LED (8.71 mW) repopulated the parent state. For the measurement of CryoR1 at pH 3.5, the time point at 468.2 ms was excluded from the dataset after data reduction due to an artifact from the coupling of the two oscilloscopes.

### Analysis of time-resolved spectroscopic data

Analysis of time-resolved spectroscopic data was performed using the OPTIMUS software (*93*). The data of the ultrafast transient absorption and transient flash photolysis measurements were objected to the model-free lifetime distribution analysis yielding the lifetime distributions of the individual photointermediate transitions, which are summarized in an LDM. The mentioned lifetimes of the photointermediate transitions were obtained from the point with the maximum amplitude of the respective lifetime distribution. For the flash photolysis measurement of CryoR1 at pH 10.5, a global lifetime analysis was performed, yielding decay-associated spectra and the lifetimes of the respective photointermediate transitions.

### Cryo-EM grid preparation and data collection

All samples were concentrated to 30 to 50 mg/ml using 100,000 MWCO concentrators (Millipore) at pH 8.0 and later mixed with a buffer with pH 8.0 (both pH 8.0 structures) or pH 4.3 (pH 4.3 structure). For grids at pH 4.3, a sample was additionally concentrated to 30 mg/ml after adding buffer and mixed again with pH 4.3 buffer, to remove excess of pH 8.0 buffer. After that, all samples were diluted (to 7 mg/ml for CryoR2 and CryoR1 datasets at pH 10.5 M and ground states, to 10 mg/ml for CryoR1 at pH 4.3 and 8.0, and to 2.7 mg/ml for CryoR1 in nanodiscs) and volume of applied onto freshly glow-discharged (30 s at 5 mA) Quantifoil grids (Au R1.2/1.3, 300 mesh) at 20°C and 100% humidity and plunged-frozen in liquid ethane. The cryo-EM data were collected using either a 300-keV Krios microscope (Thermo Fisher Scientific), equipped with a Gatan K3 detector (all datasets except for CryoR1 at pH 8.0) or 200-keV Talos Arctica (Thermo Fisher Scientific), equipped with a Gatan K2 Summit detector (CryoR1 at pH 8.0).

### Cryo-EM data processing

All steps of data processing were performed using cryoSPARC v.4.0.2 (fig. S21) (*94*). Motion correction and contrast transfer function (CTF) estimation were performed with default settings for all datasets. For datasets CryoR1 pH 8.0, pH 10.5 ground, and pH 10.5 M states, particle picking was performed using Topaz (*95*) pretrained model, followed by duplicate removal with 50-Å distance. For datasets CryoR1 pH 4.3 and pH 8.0, initial volumes were generated after picking with Topaz and blob picking, respectively, followed by template picking using generated volumes.

For datasets CryoR1 pH 10.5 ground and M states, pH 8.0 in nanodiscs, and CryoR2 pH 8.0, picked particles were extracted with 3 to 5× binning [up to 128 pixels (px)]. An initial set of particles was cleaned using two rounds of 2D classification (first round: 80 classes, 80 iterations, batch size 200 to 400, use clamp-solvent: true; second round: 20 to 40 classes, 40 iterations, batch size 50 to 200, use clamp-solvent: true). After that, particles were cleaned using a “3D classification” (ab initio model generation with five classes, followed by heterogeneous refinement; in the case of CryoR1 pH 8.0 in nanodisc, 2 cycles of 3D classification were performed; see fig. S21). These particles were reextracted without binning, followed by nonuniform (CryoR2 and CryoR1 pH 10.0 ground state) or homogeneous (CryoR1, pH 8.0 in nanodisc, pH 10.5 M state) refinement (C5 symmetry, with per-particle CTF and defocus refinement) and local (all but pH 10.5 M state) refinement (C5 symmetry), yielding final maps.

For datasets CryoR1 pH 4.3 and pH 8.0, particles were extracted using 2× binning (pH 4.3) or no binning (pH 8.0). An initial set of particles was cleaned using one round of 2D classification with default parameters (pH 4.3) or two rounds of 2D classification (first iteration: use clamp-solvent = true, second iteration: batch size 200, 20 classes). These particles were subjected to ab initio model generation with two classes, for pH 4.3 followed also by an unbinned particle reextraction. After that, particles were refined with nonuniform refinement (C5 symmetry, with per-particle CTF and defocus refinement) and local refinement (C5 symmetry, mask = dynamic for pH 8.0), yielding final maps.

### Model building and refinement

The pentameric model of CryoR1 was generated using AlphaFold (*96*) and docked as a rigid body into cryo-EM maps manually in ChimeraX. Further refinement was performed using Phenix (*97*, *98*) and Coot (*99*), producing the final statistics described in table S3. Visualization and structure interpretation were carried out in UCSF Chimera (*100*, *101*) and PyMOL (Schrödinger LLC).

### Crystallization

The crystals of CryoR2 were grown with an in meso approach (*102*), similar to that used in our previous works (*49*, *50*). In particular, the solubilized protein (80 mg/ml) in the crystallization buffer was mixed with octyl-β-d-glucopyranosid (OG, Glycon) and then with the premelted at 42°C monoolein (Nu-Chek Prep) in a 2:1 ratio (two parts of protein + OG mixture to one part of lipid) to form a lipidic mesophase. The mesophase was homogenized in coupled syringes (Hamilton) by transferring the mesophase from one syringe to another until a homogeneous and gel-like material was formed.

Then, 150-nl drops of a protein-mesophase mixture were spotted on a 96-well LCP glass sandwich plate (Marienfeld) and overlaid with 400 nl of precipitant solution by means of the Mosquito crystallization robot (SPT Labtech). Crystals were obtained with a final protein concentration of 40 mg/ml and a final OG concentration of 9% (w/v) in the water part of the mesophase. The best crystals were obtained using 1.2 M Na/K-Pi pH 4.6 as a precipitant. The crystals were grown at 22°C and appeared in 1 month.

For the determination of the CryoR2 crystal structure, once the crystals reached their final size, crystallization wells were opened, and drops containing the protein-mesophase mixture were covered with 100 μl of the precipitant solution supplemented with 15% (w/v) glycerol for cryoprotection. For data collection, harvested crystals were incubated for 5 min in the precipitant solution. Crystals were harvested using micromounts (Mitegen, USA), flash-cooled, and stored in liquid nitrogen.

### Time-resolved microspectrophotometry on the CryoR2 crystals

The spectroscopic characterization of the CryoR2 crystals was performed at the *ic*OS Lab of the European Synchrotron Radiation Facility (ESRF) (*103*) using the TR-*ic*OS instrument (*104*). Measurements were conducted at room temperature with crystals of 100 μm by 20 μm by 15 μm mounted in a dedicated holder. Series of transient UV-Vis absorption spectra were measured on the basis of a pump-probe scheme. A nanosecond laser (Surelite EX, Amplitude Technologies, France), combined with an OPO (Horizon II, Amplitude Technologies, France), was used to produce the pump signal. Laser pulses were collimated in a dedicated laser optical box into a 910-μm-diameter high-energy optical fiber (Thorlabs, USA) connected to a parabolic mirror on the top of the optomechanical setup of the TR-*ic*OS instrument. A 15× reflective objective is used to focus light at the sample position, with a ~130-μm focal spot (1/e^2^ cutoff). The probe signal was provided by a xenon flash lamp module (L11316-11, Hamamatsu Photonics, Japan) connected via a 400-μm-diameter optical fiber (Avantes, The Netherlands) to a second parabolic mirror positioned to match the optical path of the pump signal. Focusing of the probe light is achieved through the same 15× reflective objective, resulting in a ~65-μm focal spot (1/e^2^ cutoff) at the sample position. Two different wavelengths were used for the pump signal, 532 and 630 nm, with fluences of 170 and 151 mJ/cm^2^, respectively. Delays between pump and probe signals were controlled using the ESRF CITY timing module and varied between 10 μs and 10 s. Spectra were measured using a complementary metal-oxide semiconductor spectrophotometer (AvaSpec-ULS2048CL-EVO-RS-UA, Avantes, The Netherlands), with an integration time of 100 μs. In-house Python script was used to plot the data (https://github.com/ncara/TRicOS).

### Accumulation of the intermediate state in CryoR2 crystals

For the accumulation and cryotrapping of the M_2_ state, the crystal was originally kept at 100 K. It was then illuminated using a 532-nm laser with the nitrogen stream simultaneously blocked for 2 s. Because of the short illumination time, the crystal was not rotated during the illumination. For better illumination, the crystal was oriented with the largest plane perpendicular to the laser beam. The laser was then switched off once the crystal was back at 100 K. The laser power density of 10 W/cm^2^ at the position of the sample was used. The laser power was adjusted to maximize the fraction of the accumulated M_2_ state; the absence of destructive effect of the laser on crystals was justified by the remaining diffraction quality after the cryotrapping procedure.

The mean size of the crystals was 100 μm by 20 μm by 20 μm. The rod-shaped crystals were oriented so that the one of the largest planes (100 μm by 20 μm) was perpendicular to the laser beam. The laser beam was focused to the size of 200 μm by 200 μm^2^ (1/e^2^).

### Diffraction data collection and treatment

X-ray diffraction data were collected at the P14 beamline of PETRAIII (Hamburg, Germany) using an EIGER2 X 16M CdTe detector. The data collection was performed using the MxCube2 software. Diffraction images were processed using X-ray Detector Software (XDS) (*105*). The reflection intensities were scaled and merged using the Staraniso server (*106*). There is no possibility of twinning for the crystals. Diffraction data from a single crystal were used. The data collection and treatment statistics are presented in table S4.

### Crystal structure determination and refinement

Initial phases were successfully obtained in the C2221 (type A crystals) and C121 (type B crystals) space groups by molecular replacement using MOLREP (*107*) from the CCP4 program suite (*108*) using the cryo-EM structure of CryoR2 as a search model. The initial model was iteratively refined using REFMAC5 (*109*) and Coot (*110*). The structure refinement statistics are presented in table S2. For building of the structure of the cryotrapped intermediate state of CryoR2, we used the following approach. First, we calculated the difference *F*_olight_ − *F*_odark_ using the PHENIX program suite (fig. S9, H and I), which showed the structural rearrangements toward the M_2_ state structure of CryoR2 obtained using cryo-EM at pH 8.0. The 2*F*_o_ − *F*_c_ electron density maps corresponding to the dataset collected from the illuminated crystal appeared weak, particularly near the retinal and amino acid residues undergoing structural rearrangement upon formation of the intermediate state. However, the maps showed that the dominant state is the intermediate state, and the ground state is only present at a minor occupancy. Therefore, we fitted the final model of the intermediate state directly in the 2*F*_o_ − *F*_c_ maps in Coot, using the cryo-EM model of CryoR2 as a reference and *F*_olight_ − *F*_odark_ as a guide. We did not split the conformations in order not to overfit the maps and maintain reasonable geometry of the molecule. The presence of a small fraction of ground state has been also observed as <5σ peaks in the *F*_o_ − *F*_c_ difference electron density maps after automatic refinement in REFMAC5.

## Supplementary Materials

### The PDF file includes:

Supplementary Text Figs. S1 to S21 Tables S1 to S4 Legend for data S1 References
