# Supplementary material for: CryoRhodopsins: A comprehensive characterization of a group of microbial rhodopsins from cold environments
Source: Sci Adv. 2025 Jul 4;11(27):eadv1015. doi: 10.1126/sciadv.adv1015 (PMC12227055; doi:10.1126/sciadv.adv1015)
Supplement: Supplementary file 1 — Supplementary Text Figs. S1 to S21 Tables S1 to S4 Legend for data S1 References [file sciadv.adv1015_sm.pdf]

Science Advances
AAAS

Supplementary Materials for

CryoRhodopsins: A comprehensive characterization of a group of microbial rhodopsins from cold environments

Gerrit H. U. Lamm *et al.*

Corresponding author: Josef Wachtveitl, wveitl@theochem.uni-frankfurt.de; Albert Guskov, a.guskov@rug.nl; Kirill Kovalev, kirill.kovalev@embl-hamburg.de

Sci. Adv. **11**, eadv1015 (2025)
DOI: 10.1126/sciadv.adv1015

The PDF file includes:

Supplementary Text
Figs. S1 to S21
Tables S1 to S4
Legend for data S1
References

Other Supplementary Material for this manuscript includes the following:

Data S1

Supplementary Text

Structural rearrangements in the M_2 state of CryoR2

While for CryoR1 we were able to obtain the structures of the ground and M_2 states individually under the same conditions, in the case of CryoR2 we obtained several structures of the protein under different conditions using different methods. Nevertheless, they provide essential information on the structural rearrangements associated with the M state formation in CryoR2.

First, we crystallized CryoR2 using *in meso* approach and obtained two types (A and B) of purple crystals at pH 4.6 (Fig. S9A). Using the crystals of type A (C2221) we obtained the structure of CryoR1 at 2.45 Å with X-ray crystallography. The crystals of type B (C2) diffracted more anisotropically to 3.0 Å and revealed a similar structure to that obtained with type A crystals. Therefore, we further discuss the 2.45 Å crystal structure of CryoR2.

The structure of CryoR2 shows similar overall organization to that of CryoR1 in the ground state (Fig. S9B,C). As in CryoR1, the extracellular side of CryoR2 has a large internal cavity, separated from the bulk by a short N-terminal α-helix (Fig. S9B). The side chain of R58 (R57 in CryoR1) is directed to the cytoplasmic bulk but not to the RSB. The residues in the helices F and G and the backbone of helix G are located same to the analog residues of CryoR1 in its ground state, including residues L204, W208, and F245 (L203, W207, and F244 in CryoR1) (Fig. S9C). Microspectrophotometry showed that a major fraction of CryoR2 molecules in crystal contains protonated RSB counterion complex as indicated by the red-shifted maximum absorption wavelength (Fig. S9D,E). A part of molecules retain the deprotonated complex (Fig. S9D,E). Lastly, a small fraction of the protein molecules is in the blue-shifted state characteristic for the deprotonated RSB (Fig. S9D,E). The relatively low resolution didn't allow us to resolve multiple conformations of the RSB counterion complex associated with different protonation states. Nevertheless, the structural and spectroscopy data suggest that the above-described structure reflects that of the ground state of CryoR2.

Next, time-resolved spectroscopy on the CryoR2 crystal showed a pronounced formation of the blue-shifted state upon green (532 nm) or red (630 nm) light illumination (Fig. S9D-G). Noteworthy, the occupancy of the blue-shifted state was larger in the case of green light illumination, indicating that the red-shifted form with protonated counterion complex likely is not photoconvertible, similar to that observed for the red-shifted state of CryoR1 at pH 3.5. We cryotrapped the blue-shifted intermediate state in the crystal of CryoR2 using the protocol similar to that we used in recent works for the trapping of the M states of BR(*47*) and *Bc*XeR(*52*) (see Materials and Methods for details). Due to the limited amount of the type A crystals, we used type B crystals for the cryotrapping of the intermediate state. The F_{olight}-F_{odark} difference electron density maps at 3.0 Å clearly showed the rearrangements at the cytoplasmic side of CryoR2, including the flip of the R58 side chain towards the RSB and synchronous reorientations of the residues in helices F and G similar to those found in the M_2 state of CryoR1 (Fig. S9H,I). Different from CryoR1, the maps suggest the flip of the L111 residue (L110 in CryoR1) in the M_2 state of CryoR2 (Fig. S9H,J). Since the cryotrapping protocol results in the accumulation of the longest-living intermediate, the observed rearrangements were assigned to the M_2 state of the CryoR2 photocycle.

In addition to X-ray crystallography, we obtained a single-particle cryo-EM structure of CryoR2 at pH 8.0 at 2.44 Å resolution (Fig. S10A,B). The sample was kept under the overhead light before the application to the cryo-EM grid. Under such conditions, the blue-shifted M_2 state is accumulated in the sample and is represented in the major fraction of protein molecules (Fig. S10C). The cryo-EM map revealed the crystal structure of the cryotrapped M_2 state. Due to higher resolution, more details were resolved in the cryo-EM maps. Thus, the retinal is in likely the 13-*cis* conformation, similar to that of CryoR1 in the M_2 state (Fig. S10B). The side chain of R58 is oriented towards the RSB (Fig. S10B,D,E). The L111 side chain is flipped compared to the ground state structure and an additional density is found near the RSB in the M_2 state of CryoR2. We assigned it to a water molecule, which likely stabilizes the deprotonated form of the RSB in the intermediate. The RSB is additionally stabilized by S107 of the functional motif.

In summary, X-ray crystallography, cryo-EM, time-resolved absorption microspectrophotometry on crystals, and the cryotrapping of the intermediate state allowed us to show that the structural changes associated with the formation of the M_2 state in CryoR2 are very similar those shown in the case of CryoR1. Since the arginine at the cytoplasmic side plays one of the key roles and is a unique characteristic residue in the entire CryoRs clade, we suggest that the flipping motion of the arginine is conserved within all CryoRs.

At the same time, we found differences between the M state structures of CryoR1 and CryoR2. Namely, both cryo-EM and X-ray crystallography data show the flip of the L111 residue in the intermediate state of CryoR2, while no flip of L110 (analog of L111 in CryoR2) in the M_2 state of CryoR1. Furthermore, while in CryoR1 the RSB is connected to the D237 and oriented towards helix G in both the ground and the M_2 states, in CryoR2 the RSB is always oriented to helix C and is H-bonded to the S107. Thus, we speculate that the differences between the M_2 state structures of two CryoRs originate from the more polar environment of the cytoplasmic side of the RSB region in CryoR2 than that of CryoR1. The role of the additional water molecule remains elusive; however, we speculate that it stabilizes the deprotonated RSB in the M_2 state.

Protonation states of the counterions in CryoRs

In the course of the study of CryoRs, we identified unusual spectral behavior of the members of this clade. Together with the extremely long-living blue-shifted state, the RSB counterion complex of CryoR1 and CryoR2 demonstrate multiple protonation states as indicated by the pH titration of the maximum absorption spectra (Fig. S7). The structural data on CryoR1 at different pH values and in various functional states allowed us to get insights into the mechanisms of the proton storage at the RSB counterion complex of these rhodopsins.

In CryoR1, there are three major spectral states corresponding to the different protonation states of the RSB and its surroundings. According to the spectroscopy data, these states are nicely represented at pH ranges of 2.5-4.5 (620 nm), 7.5-9.0 (550 nm), and 10.5-11.5 (540 nm). To understand the differences of the maximum absorption wavelengths, we obtained the cryo-EM structures of CryoR1 at pH 4.3, 8.0, and 10.5 to have representative models for all three states identified in the spectroscopy experiments (Table S2).

Surprisingly, in the RSB region, the two counterions (E102 and D237) interact directly with each other in the ground state at all studied pH values (Fig. S17). This indicates that there is always at least one proton in the RSB counterions complex of CryoR1. In the 2.43 Å resolution structure of CryoR1 at pH 8.0, D237 is stabilized by Y68 and Y210 and interacts directly with the protonated RSB, which indicates that the residue is likely deprotonated (Fig. S17B). Thus, the proton is stored at E102 in the ground state of CryoR1 at neutral pH. At pH 10.5, the D237 side chain clearly reorients; however, we should note that at 2.7 Å resolution, it is impossible to univocally identify the exact position and orientation of the counterions. Nevertheless, in accordance with the spectroscopy data showing the ~10 nm blue-shift of absorption maximum between pH 8.0 and 10.5 (Fig. S7), the conformation of the RSB region is also changed. The distance between E102 and D237 is shortened, which might be a signature of proton delocalization between the carboxylic residues (Fig. S17C). Thus, we suggest that the blue-shift of the spectrum upon pH increase from neutral to alkaline is associated with the proton redistribution rather than deprotonation of any of the counterions.

At pH 4.3, the conformation of the RSB-counterions complex is different from that at pH 8.0 and 10.5 (Fig. S17A). Namely, the protonated RSB is likely H-bonded to E102 instead of D237. D237 is reoriented at pH 4.3 compared to its position at pH 8.0 (Fig. S17A,B). While D237 remains H-bonded to the same oxygen atom OE1 of E102, as well as to Y68, the distance between Y68 and D237 is enlarged to 3 Å. This indicates that D237 might be protonated at pH 4.3. As the spectrum of D237N mutant at neutral pH is strongly red-shifted and close to that of CryoR1-WT at low pH, one can assign the rise of the 620-nm-absorbing state to the protonation of D237, which tentatively indicates the pK_a of D237 to be ~6.5 (Fig. S7). We speculate that E102 is also protonated at pH 4.3; the proton is stored at the OE2 atom of the glutamic acid. The distance between E102 and D237 at low pH is shorter than that at pH 8.0, which might indicate proton delocalization between the residues, similar to that at pH 10.5. Indeed, at acidic pH values, CryoR1 demonstrates a notable (80 nm) red-shift of the absorption spectra (Fig. 3G; Fig. S7). Spectroscopy studies of the D237N, E102Q, and E102Q/D237N mutants, mimicking various protonation states of the RSB counterion complex, showed that none of these mutants result in such a strong red-shift as that found in the WT protein upon pH decrease (Fig. 3H,I). This supports our hypothesis on the proton delocalization with the unique resulting configuration of the RSB region, which is only possible within carboxylic, but not amide groups. As it was mentioned in the manuscript, the spectral behavior of CryoR1 is similar to that of the bestrhodopsin Tara-RRB(*45*), which has been also shown to likely have carboxylic residues interacting directly to each other in the RSB region. Thus, although CryoRs and bestrhodopsins are structurally very different, the fundamental principles for the different protonation states of the counterion complexes and their spectral signatures might originate from the similar proton delocalization between the residues in the two clades of MRs.

In the M_2 state of CryoR1 at pH 10.5, the RSB is deprotonated; however, it is likely still connected to the D237 residue (Fig. S17D). Thus, we suggest that the RSB proton is transferred to D237 upon light illumination (Fig. 8A,B; Fig. S17C,D). E102 remains protonated in both the ground and the M_2 states, as indicated by a direct H-bond between E102 and D237 similar to that in the ground state of CryoR1 at pH 8.0. Thus, both counterions are likely protonated in the M_2 state. This observation is in line with the spectroscopy study of the E102Q/D237N mutant mimicking the fully protonated counterions complex (Fig. S8C). In the mutant, the RSB

is deprotonated at both neutral and alkaline pH (Fig. S8C). This clearly indicates that the protonated counterions complex stabilizes the deprotonated form of the RSB, characteristic to the M_2 state.

In summary, the counterion complex of CryoR1 is likely single-protonated at neutral and alkaline pH values with the proton localized at E102 but possibly delocalizing between E102 and D237 at alkaline pH. Single protonation allows an efficient proton transfer from the RSB to D237 with the rise of the blue-shifted state. The latter event results in the double protonated state of the E102-D237 pair, stabilizing the deprotonated RSB and thus contributing to the extremely slow decay of the M_2 intermediate. At low pH, the RSB and both counterions are protonated, which hampers the formation of the blue-shifted state and notably accelerates the photocycle (Fig. 3D,E; Fig. S6A). It should be noted, that at acidic pH values, the protonation of both counterions also likely hampers retinal isomerization as indicated by an extremely small fraction of the protein molecules entering the photocycle upon light illumination (Fig. 3E).

In the case of CryoR2, the structural data on various protonation states is limited. Nevertheless, both the cryo-EM and the crystal structures show that, in contrast to CryoR1, the RSB of CryoR2 is H-bonded to S107 (A106 in CryoR1) of the functional motif (Fig. S17E,F). D238 is H-bonded to Y69 and Y211 at pH 4.6 and 8.0, tentatively indicating that the residue is deprotonated in a wide range of pH values. E103 interacts directly with D238 in both structures; thus, E103 is likely protonated. Since the cryo-EM structure of CryoR2 at pH 8.0 likely represents the M_2 state, we conclude that the counterions complex in the M_2 intermediate of CryoR2 is single protonated, unlike that of CryoR1. This is supported by the spectroscopy analysis of the E103Q mutant of CryoR2, which showed similar spectral properties to those of E102Q/D237N of CryoR1 (Fig. S8C,D). Namely, the RSB is deprotonated in the E103Q mutant indicating that single protonation of E103 is sufficient to stabilize the M_2 state of CryoR2. These results also likely mean the deprotonated RSB counterions complex at neutral and alkaline pH in the ground state of CryoR2.

At low pH, we have not observed any structural footprints of the D238 protonation. Similar to the M_2 state at pH 8.0, the E103 residue is considered protonated as it forms an H-bond with deprotonated D238 (Fig. S17E). The deprotonation of the RSB proceeds effectively in CryoR2 at pH 3.5 (Fig. S6B); therefore, the RSB counterions complex is likely single protonated under these conditions in the ground state. Thus, we hypothesize that, unlike in CryoR1, the counterion complex of CryoR2 is deprotonated at neutral and alkaline pH, and is single protonated at low pH values (Fig. S17E,F). This results in the blue-shifted state formation at all studied proton concentrations, but also explains the differences in the spectral behavior of CryoR1 and CryoR2 (Fig. S3).

Thus, structural data demonstrates the possible molecular mechanism of spectral variations within the CryoRs clade by alteration of the amino acid residue of the functional motif (A106/S107 in CryoR1/CryoR2). At the same time, spectroscopy data on the CryoR1 and CryoR2 mutants together with high-resolution structures of the proteins at different pH values demonstrate that the proton is likely transferred from the RSB to the counterion complex and stored in it; the protonated complex stabilizes the deprotonated form of the RSB contributing to the extremely slow decay of the blue-shifted intermediate in CryoRs.

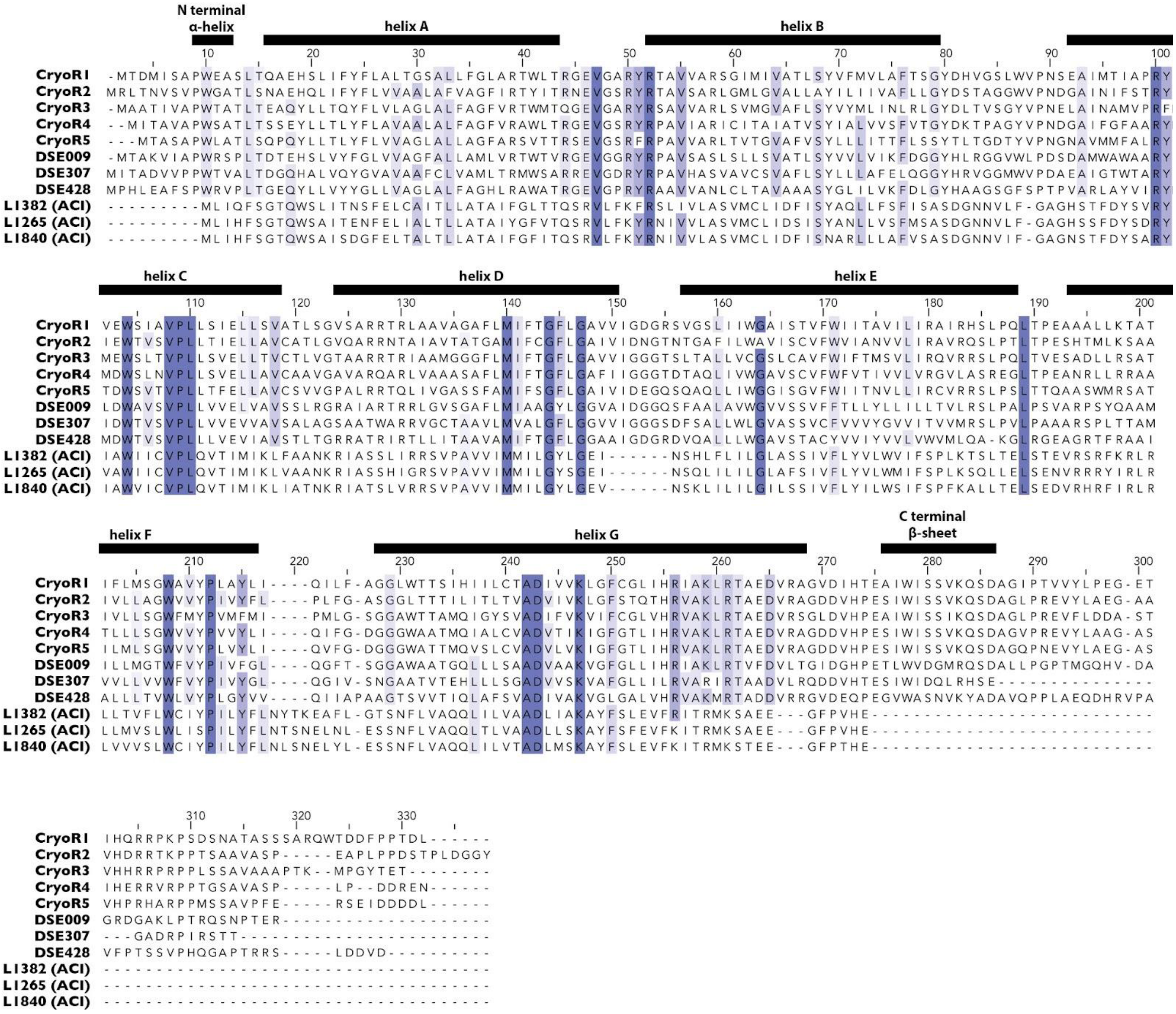

Fig. S1. Multiple sequence alignment of CryoRs, DSE, and ACI rhodopsins.

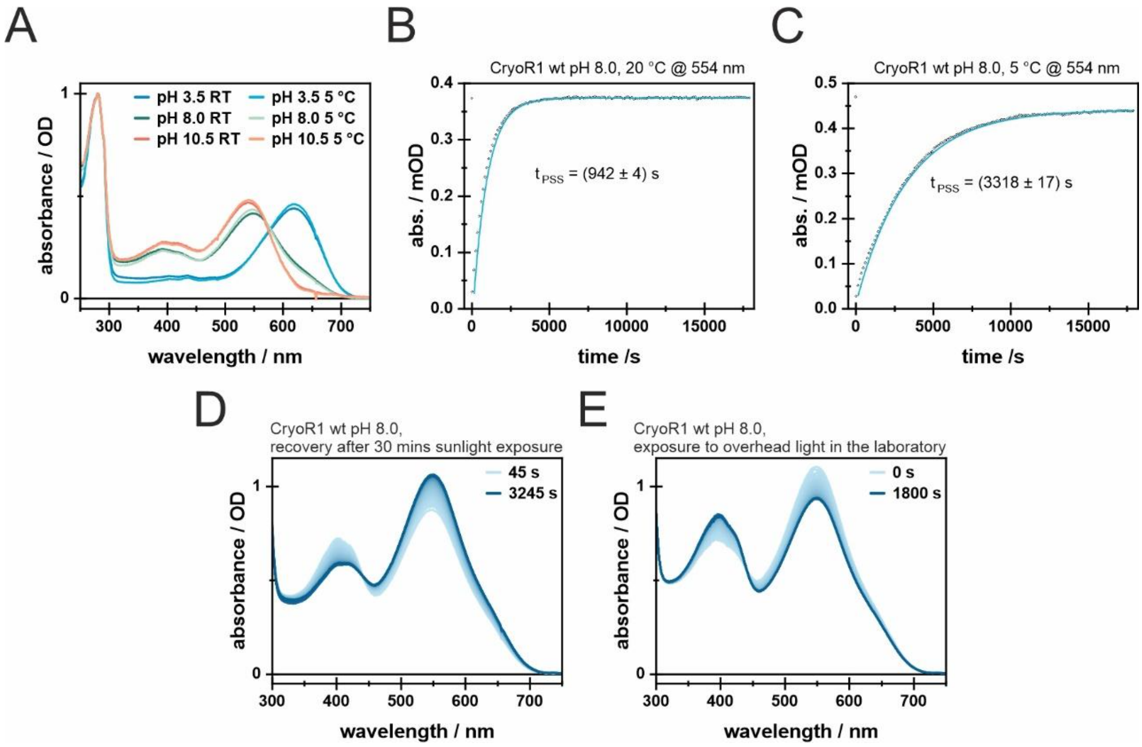

Fig. S2. Temperature and light dependent behavior of CryoR1. A. Absorption spectra of CryoR1 wt at pH levels 3.5, 8.0, and 10.5 at 5 °C and room temperature. Absorption intensity of CryoR1 at pH 8.0 monitored at 554 nm to track the recovery of the parent state after formation of the PSS at **B.** 20 °C and **C.** 5 °C. **D.** Recovery of spectral changes induced by 30 mins exposure to sunlight. The first spectrum was measured ~45 s after removing the sample from sunlight. Spectra were measured every 30 s for a duration of 1 h. **E.** Spectral changes induced on the CryoR1 dark state spectrum by exposure to overhead in the light in the laboratory. Spectra were measured every 30 s for a duration of 30 mins.

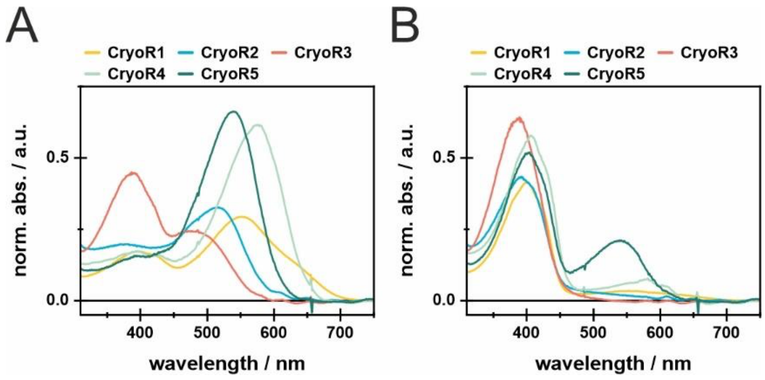

Fig. S3. Dark state and PSS spectra for CryoR1-5 at pH 8.0. A. Dark state spectra of all investigated CryoRs at pH 8.0. **B.** Spectra of the populated PSS for all investigated CryoRs after illumination of the main absorption band in the range of 530 nm to 590 nm. The output powers for the used LEDs are provided in the methods section.

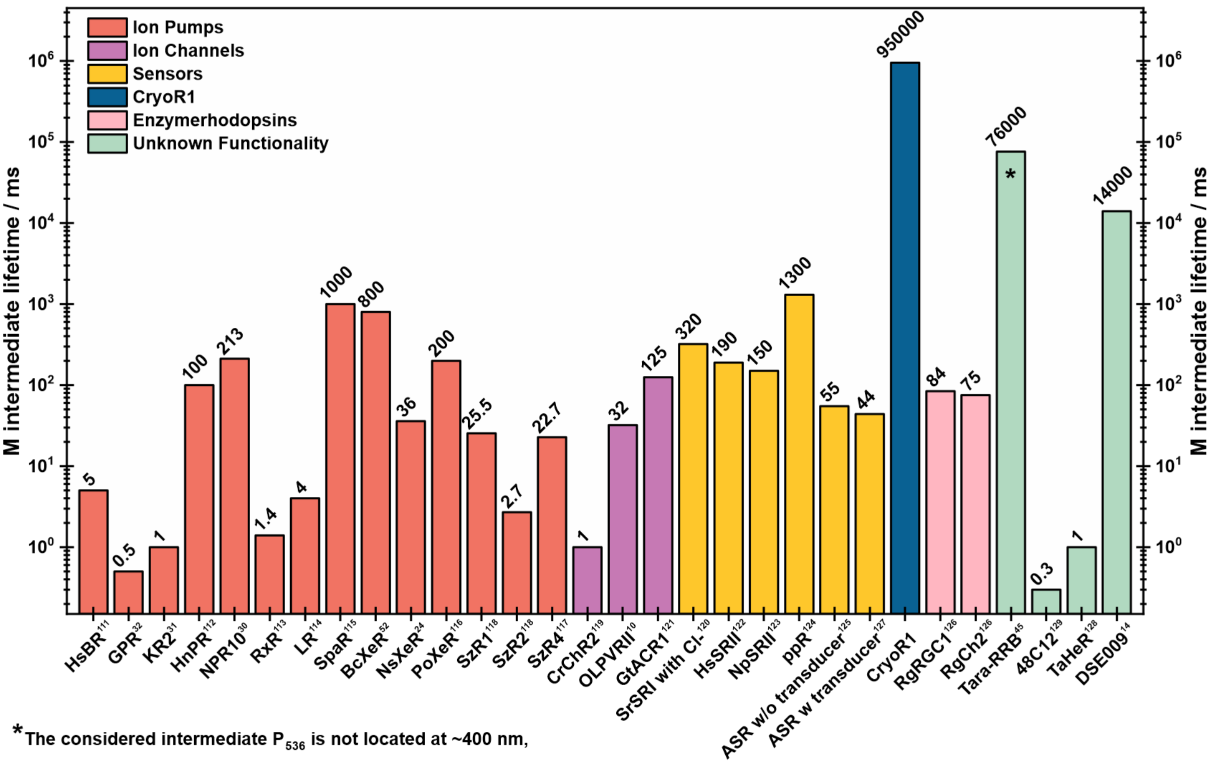

*The considered intermediate P_{536} is not located at ~400 nm, but still significantly blue-shifted compared to the dark state (661 nm).

Fig. S4. Overview of M intermediate decay kinetics in a selection of microbial rhodopsins. The lifetime of the M intermediate decay provided in ms is compared among a selection of 30 microbial rhodopsins(*10*, *14*, *24*, *30–32*, *45*, *52*, *111–129*). The selection consists of well-characterized examples and more recently discovered ones. Furthermore, the representation has been color coded to indicate the functionality of the respective microbial rhodopsin. The currently slowest characterized microbial rhodopsin NeoR has not been considered for the comparison, since no value is provided. Additionally, according to the provided data, NeoR can be considered as bistable. Note: This comparison does not claim to be all-embracing.

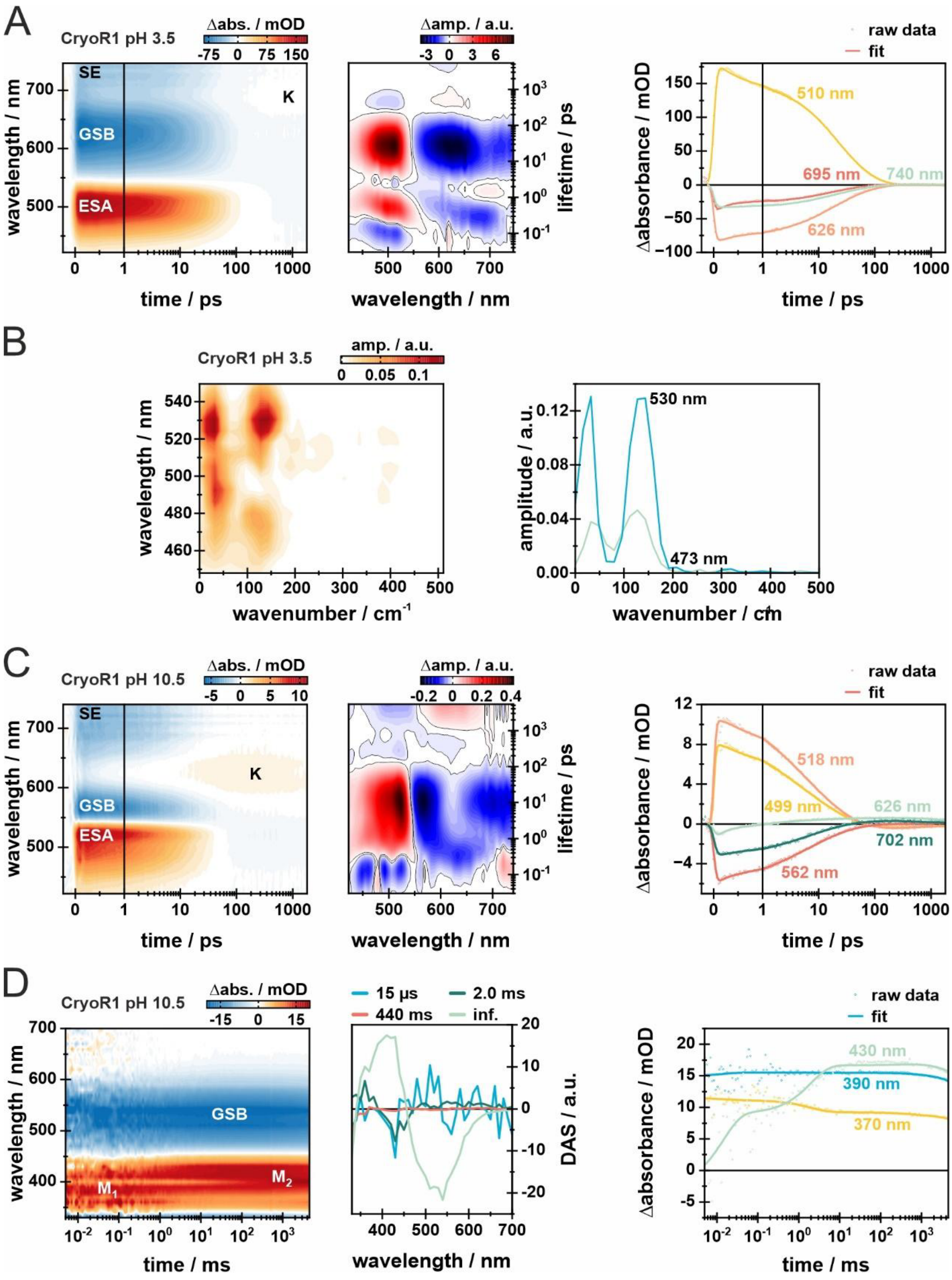

Fig. S5. Overview of the time-resolved spectroscopic characterization of CryoR1. Ultrafast dynamics of CryoR1 at **A** pH 3.5 and **C** shown as a 2D-contour plots. The x-axis is linear until 1 ps and logarithmic afterward. The signal amplitude is color coded as follows: positive (red), no (white) and negative (blue) Δabs. Additionally, the corresponding lifetime density maps (LDM) are shown illustrating the kinetics components of the respective measurement. Furthermore, transients at specific wavelengths are shown. The raw data is shown as dots, while the obtained fit is shown as lines. **B** Fourier-transformed spectra of CryoR1 at pH 3.5 as a 2D-contour plot (left) and as Fourier-transformed spectra at two specific wavelengths. **D** Flash-

photolysis measurement of CryoR1 at pH 10.5 is shown as a 2D-contour plot. The photocycle was measured until 4.5 s and ended afterwards via illumination with a 405 nm LED (8.71 mW) before each acquisition. Decay-associated spectra (DAS) are shown retrieving lifetimes of the observed photointermediate transitions. Furthermore, transients at characteristic wavelengths shown.

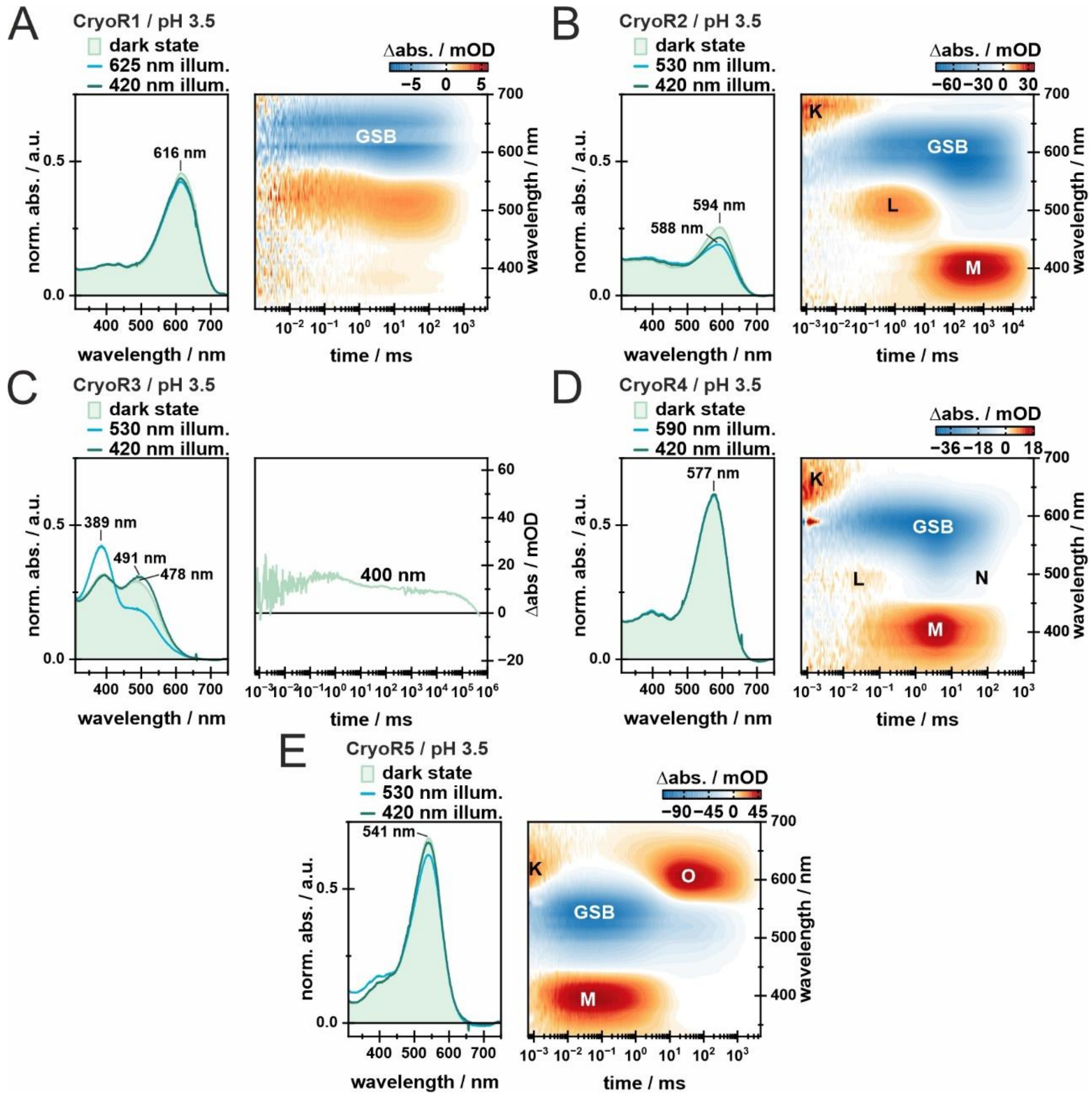

Fig. S6. Illumination experiments and photocycle kinetics of CryoRs at low pH. In each panel, the dark state spectra (light green), as well as the PSS spectra after illumination of the main absorption band (blue) and the spectra after illumination of the obtained PSS with blue light (dark green) to recover the dark state of each investigated cryoR are shown. Each LED was turned on for 100 s. Output powers for LEDs in the 500 nm range are given in the methods section. The 420 nm LED was operated at 0.19 mW. The 625 nm LED was operated at 6.4 mW. Additionally, for all investigated CryoRs except CryoR3 the measured photocycle kinetics were depicted as 2D-contour plots. The signal amplitude is color coded as follows: positive (red), no (white) and negative (blue) Δabs. The observed photocycle intermediates are named in the common way for microbial rhodopsins based on the photocycle of bacteriorhodopsin. Due to the slow photocycle kinetics, only the 400 nm transient was measured and is shown for CryoR3, similar to the measurements at high pH shown in Fig. 2 in the main text.

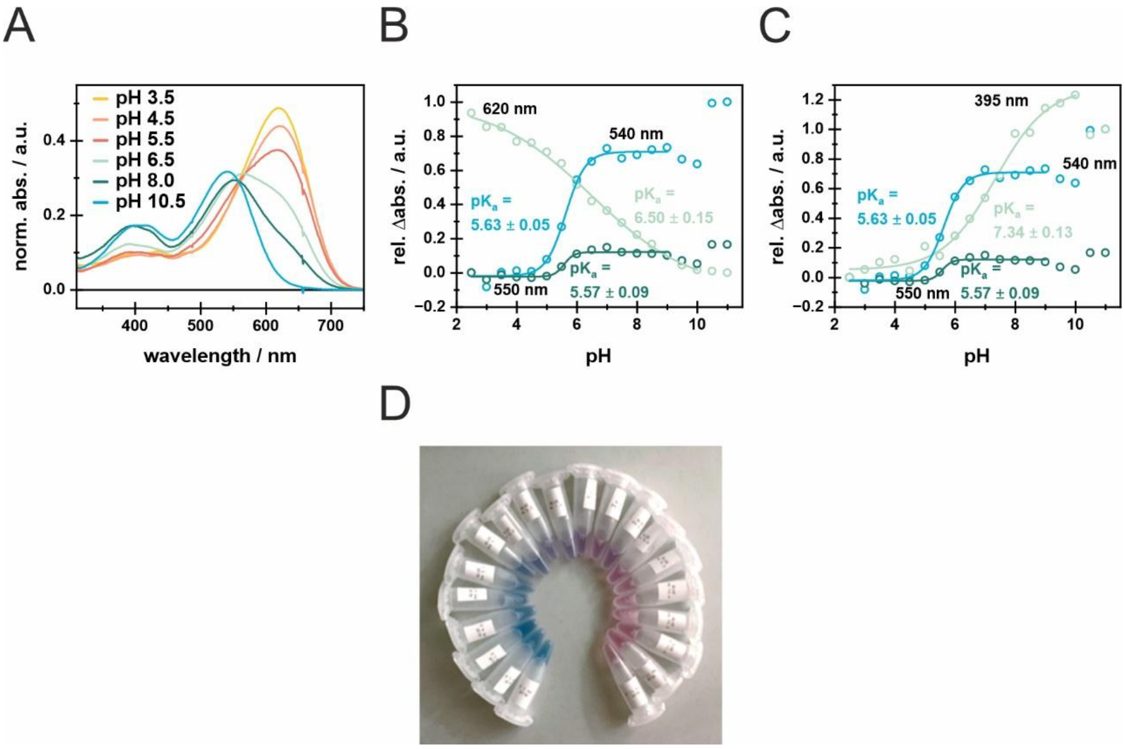

Fig. S7. pH titration of CryoR1 in the range of pH 2.5 to pH 11.0. A absorption spectra at specific pH values indicating the observed pH-dependent shift of the absorption maximum. Furthermore, the change in absorption is shown in a pH-dependent manner for **B** 540 nm, 550 nm and 620 nm and **C** 395 nm, 540 nm and 550 nm. The absorption changes are shown as relative absorption changes. For 620 nm it was assumed that the maximum amplitude is reached at pH 2.5 and therefore the obtained value was set to 1. For all other shown wavelengths, it was assumed that the maximum amplitude is reached at pH 11.0, so the rel. *Δ*abs. was set to 0 at pH 2.5. pH induced spectral changes have been fitted by the Henderson-Hasselbalch equation to determine pK_a values. Spectral changes at high pH did not result in a converging fit. Thus, they were not considered. **D** shows the samples used to measure the absorption spectra for the pH titration in the range of pH 2.5 (left) to pH 12.0 (right).

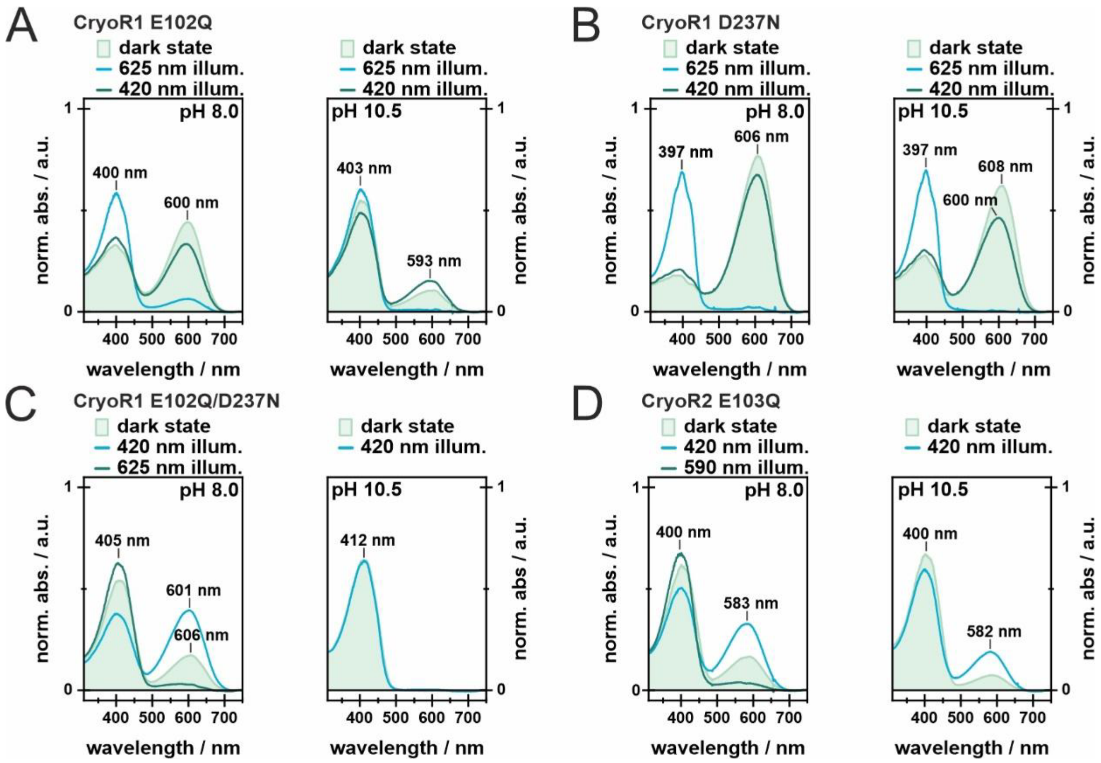

Fig. S8. Illumination experiments of mutants of charged residues of CryoR1 and CryoR2 in their retinal binding pocket. Dark state and PSS spectra of CryoR1 mutants E102Q **A**, D237N **B,** and E102Q/D237N **C**, as well as CryoR2 E103Q **D**. First of all, the dark state spectrum was measured, followed by the PSS after illumination of the main absorption band for 100s and the illumination of the potentially rising band for 100s afterward. The 625 nm LED was operated at 6.4 mW, the 590 nm LED was operated at 0.26 mW and the 420 nm LED was operated at 0.19 mW.

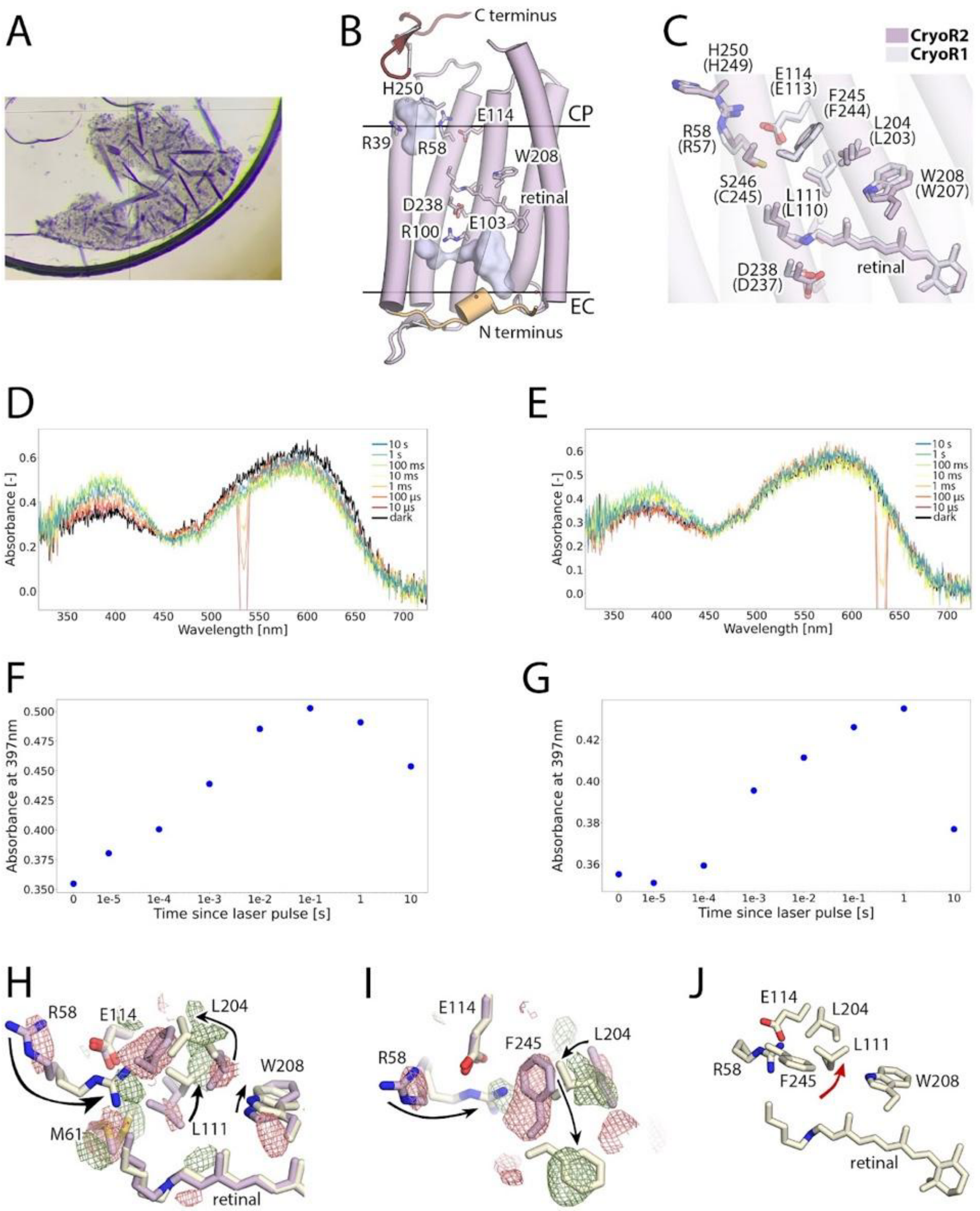

Fig. S9. Crystal structures of CryoR2. A. Crystals of CryoR2 grown using *in meso* approach at pH 4.6. Crystals of two types (A and B) were found in the same crystallization well. **B.** Overall side view of the CryoR2 protomer at pH 4.6. N terminus is colored light orange. C terminus is colored red. **C.** Structural alignment of the CryoR1 (cryo-EM, pH 10.5, ground) and CryoR2 (X-ray, pH 4.6, ground) structures. Residues of CryoR2 are signed (the corresponding residues of CryoR1 are given in parenthesis). **D.** Spectra of CryoR2 crystal. **E.** Spectra of CryoR2 crystal. Black line corresponds to the dark spectra. Then, the evolution of the spectrum is shown in the time range of 10 µs to 10 s following the nanosecond green (532 nm, panel **D)** and red (630 nm, panel **E**) laser illumination of the crystal. **F.** Evolution of the absorption in the range of 392-402 nm corresponding to the blue-shifted state in the CryoR2 crystals following the nanosecond green (532 nm) laser illumination. **G.** Evolution of the absorption in the range of 392-402 nm corresponding to the blue-shifted state in the CryoR2 crystals following the nanosecond red (630 nm) laser illumination. **H.** Side view of the structural rearrangements in CryoR2 upon cryotrapping of the M_2 state. **I.** View from the cytoplasmic side of the structural rearrangements in CryoR2 upon cryotrapping of the M_2 state. Difference F_{olight}-F_{odark} electron density maps are contoured at the level of 3.0σ. Black arrows

indicate the key rearrangements associated with the M_2 state formation. **J.** The cytoplasmic side of the CryoR2 protomer in the M_2 state obtained with X-ray crystallography. Red arrow indicates the flipped side chain of L111, not found in the M_2 state of CryoR1.

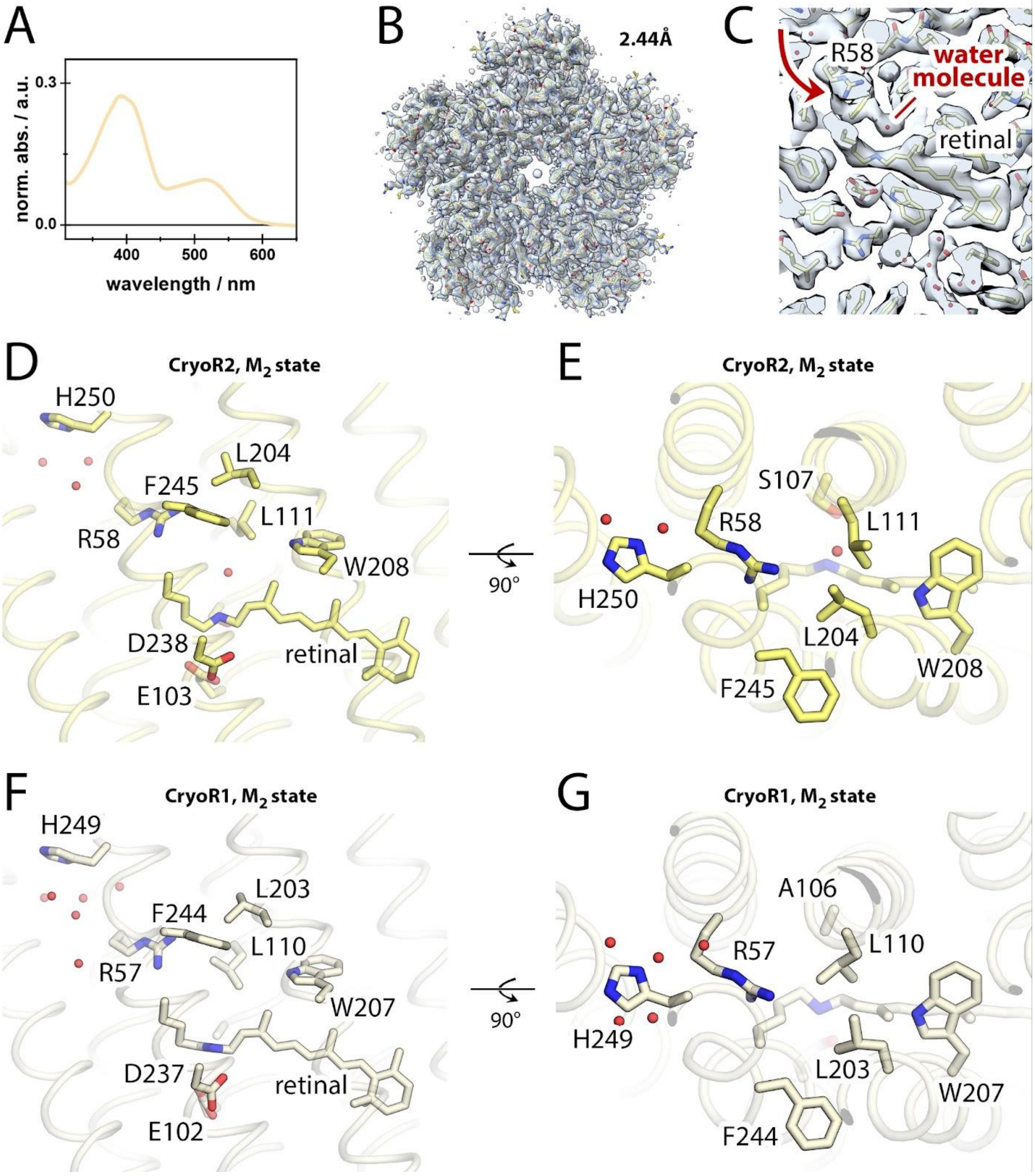

Fig. S10. Cryo-EM structure of CryoR2. A. Spectra of the CryoR2 sample used for grid preparation. The sample was kept under overhead lights for 1h. **B.** Overall cryo-EM map of the pentameric CryoR2 at pH 8.0. **C.** Section view of the cryo-EM map in the region of retinal and R58. The flipped side chain of R58 in the M_2 state is shown with a red arrow. An additional water molecule compared to the M_2 state of CryoR1 is indicated with a red line. **D.** Side view of the cytoplasmic part of CryoR2 in the M_2 state. **E.** View from the cytoplasmic side of the cytoplasmic part of CryoR2 in the M_2 state. **F.** Side view of the cytoplasmic part of CryoR1 in the M_2 state. **G.** View from the cytoplasmic side of the cytoplasmic part of CryoR2 in the M_2 state.

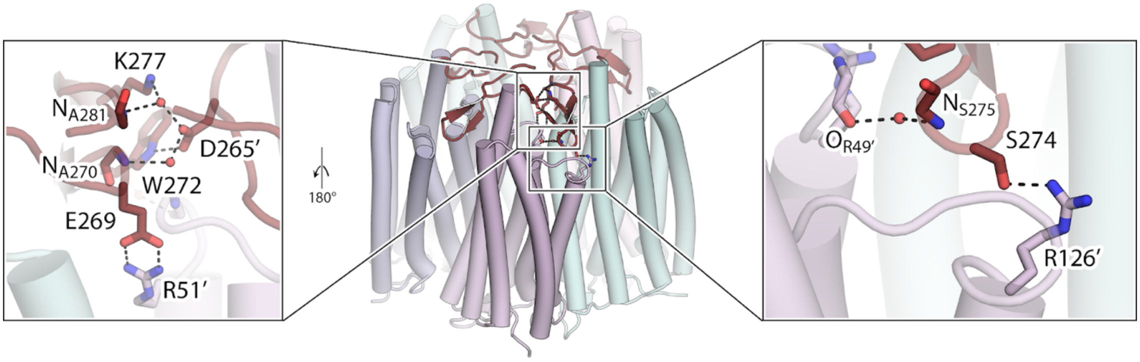

Fig. S11. Involvement of the C-terminus in stabilizing the CryoR1 pentamer. The overall view of the pentamer of CryoR1 (center) with the C-terminus colored red. Detailed view of the interprotomeric contacts formed by the tip of the β-sheet of the C-terminus (colored red) with the nearby protomer (colored light purple) (right). Detailed view of the interprotomeric contacts formed by the C-terminus in the region closer to the central axis of the pentamer (left). For clarity, the view in the left panel is from the center of the pentamer.

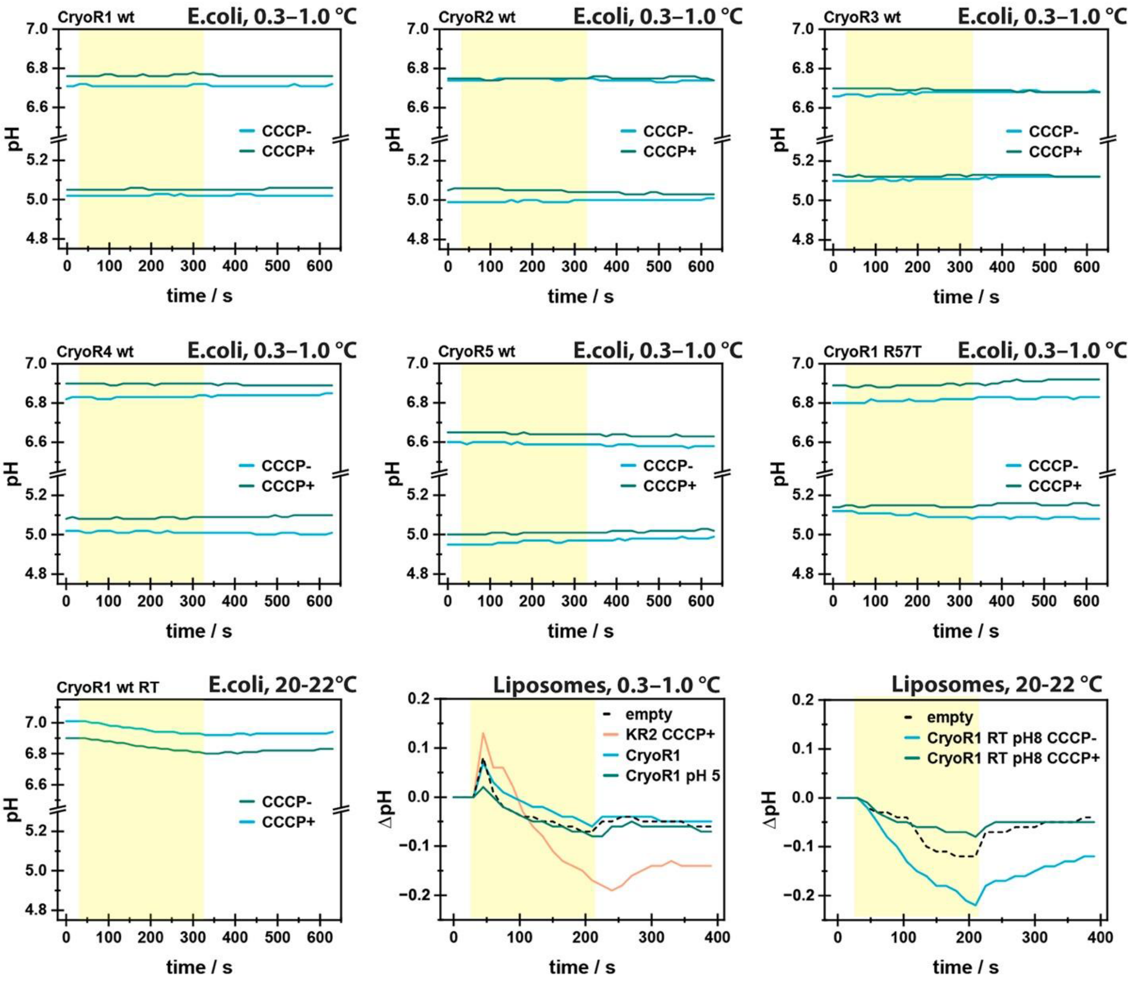

Fig. S12. Functional tests of CryoRs in *E. coli* cell and proteoliposomes suspensions. Light-induced pH changes in a suspension of *E. coli* expressing CryoRs in non-buffered 100 mM NaCl solution. Experiments were performed at two pH values; pH was adjusted using 10% HCl. Cyan lines represent results from experiments without any protonophore; green lines show pH changes in the presence of 30 μM of protonophore carbonyl-cyanide m-chlorophenyl-hydrazone (CCCP). The yellow area indicates the period of time in which the white light was on.

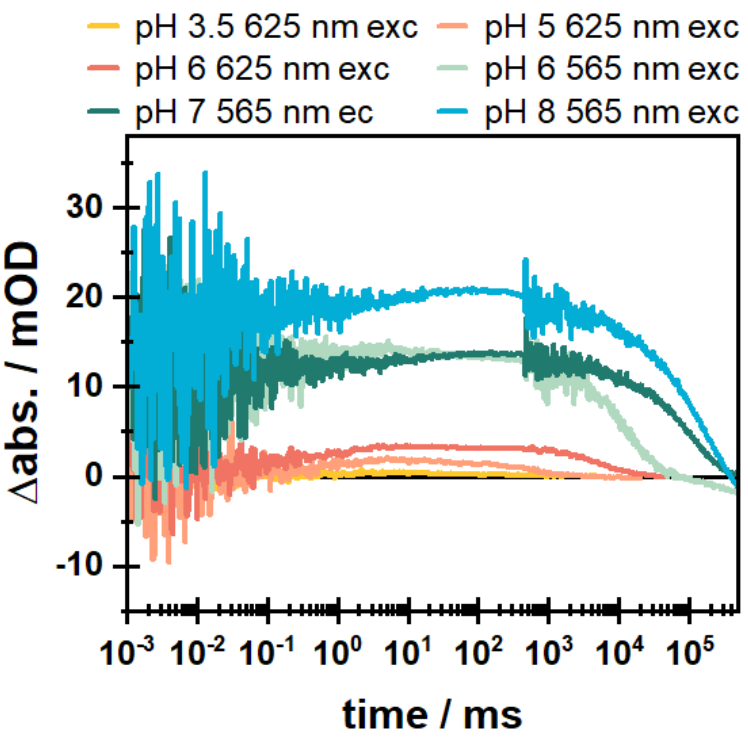

Fig. S13. pH and excitation wavelength dependence of the CryoR1 M-state kinetics.

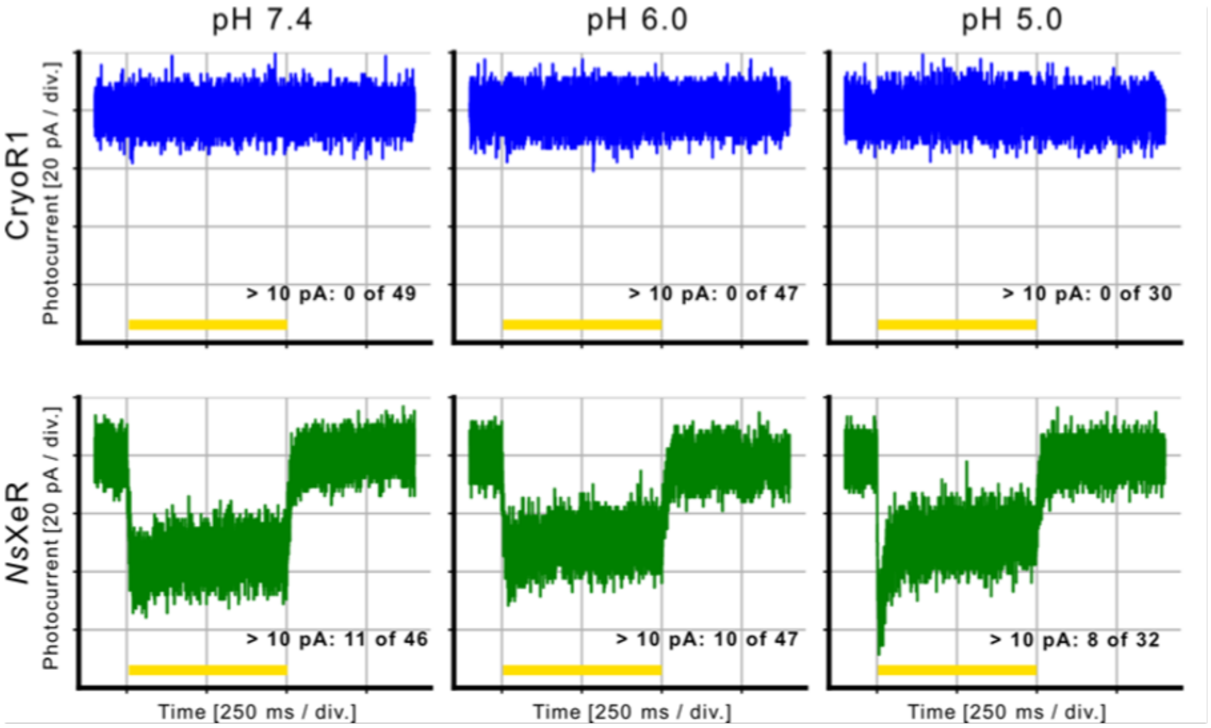

Fig. S14. Photocurrents of CryoR1-expressing NG108-15 cells recorded using planar patch-clamp at different pH levels. The top row shows photocurrents from a representative NG108-15 cell expressing CryoR1, while the bottom row shows a representative cell expressing the inward proton pump *Ns*XeR, used as a positive control. pH was changed both intra- and extracellularly in all cells. Measurements were performed at three pH conditions: (left) pH_o 7.4 / pH_i 7.2, (center) pH_o 6.0 / pH_i 6.0, and (right) pH_o 5.0 / pH_i 5.0. The number of cells exhibiting detectable photocurrents versus the total number of high-quality recordings is indicated in the bottom-right corner of each panel.

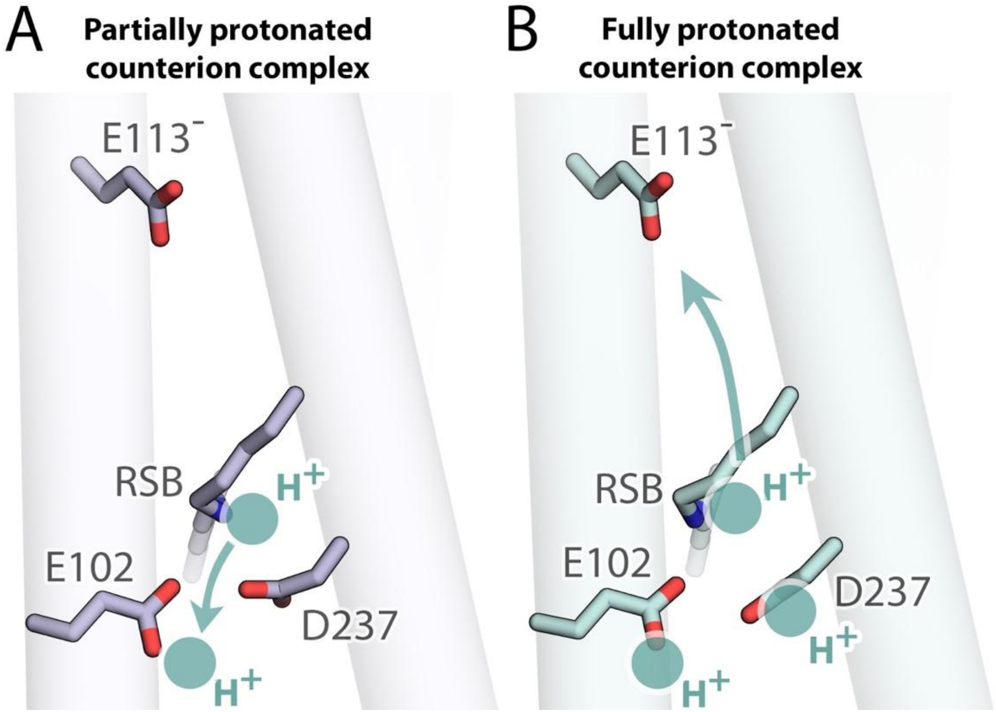

Fig. S15. Proposed scheme of the proton transfer in CryoR1. A. The RSB proton transfer towards the partially protonated counterion complex upon activation with the 500 nm laser pulses. B. The RSB proton transfer towards the cytoplasmic side to the deprotonated E113 residue upon illumination with the 620 nm laser pulses when the counterion complex is fully protonated.

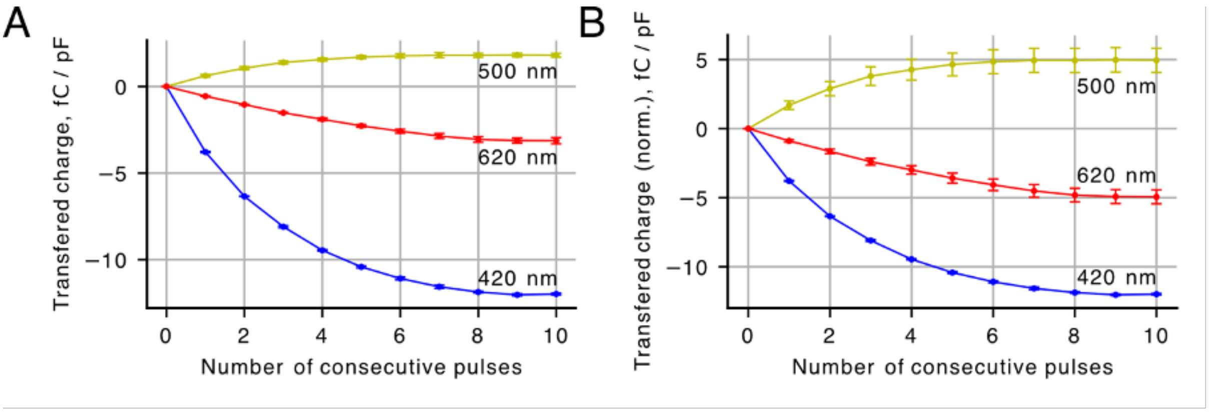

Fig. S16. Quantification of the transferred charge. A. Cumulative charge transferred at a membrane voltage of 0 mV in response to illumination with consecutive light pulses of 420 nm (for the M_2 state activation), 500 nm, and 620 nm, normalized by cell capacitance (number of cells, N = 2). **B.** Cumulative charge transferred at a membrane voltage of 0 mV in response to illumination with consecutive light pulses of 420 nm (for the M_2 state activation), 500 nm, and 620 nm, normalized by cell capacitance. The curves for 500 nm and 620 nm activation were further normalized by the fractions of the protein in the respective subpopulations (number of cells, N = 2).

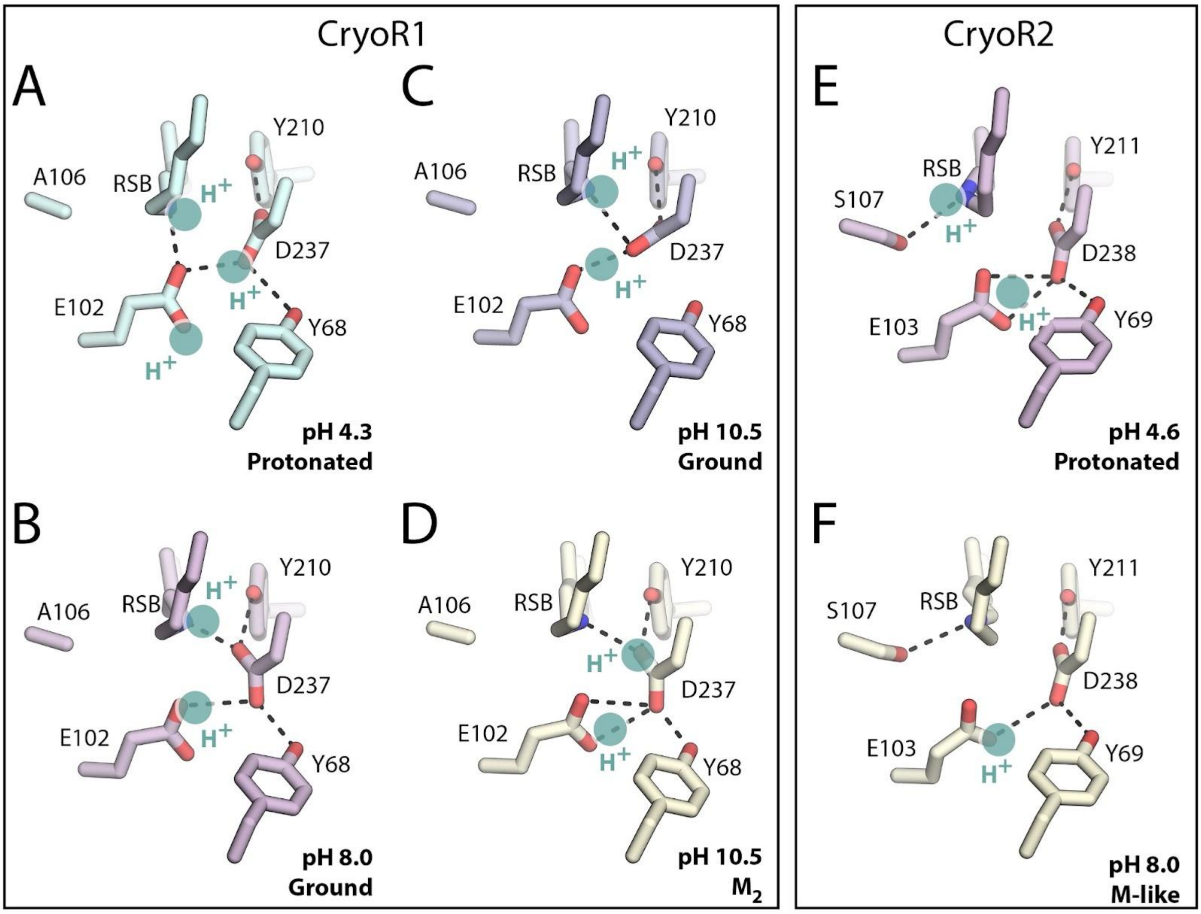

Fig. S17. The RSB region of CryoR1 and CryoR2. A. The RSB region of CryoR1 at pH 4.3. **B.** The RSB region of CryoR1 at pH 8.0. **C.** The RSB region of CryoR1 at pH 10.5 in the ground state. **D.** The RSB region of CryoR1 at pH 10.5 in the M_2 state. **E.** The RSB region of CryoR2 at pH 4.6. **F.** The RSB region of CryoR2 at pH 8.0. Putative protons in the RSB region are shown with cyan circles. H-bonds are shown with black dashed lines.

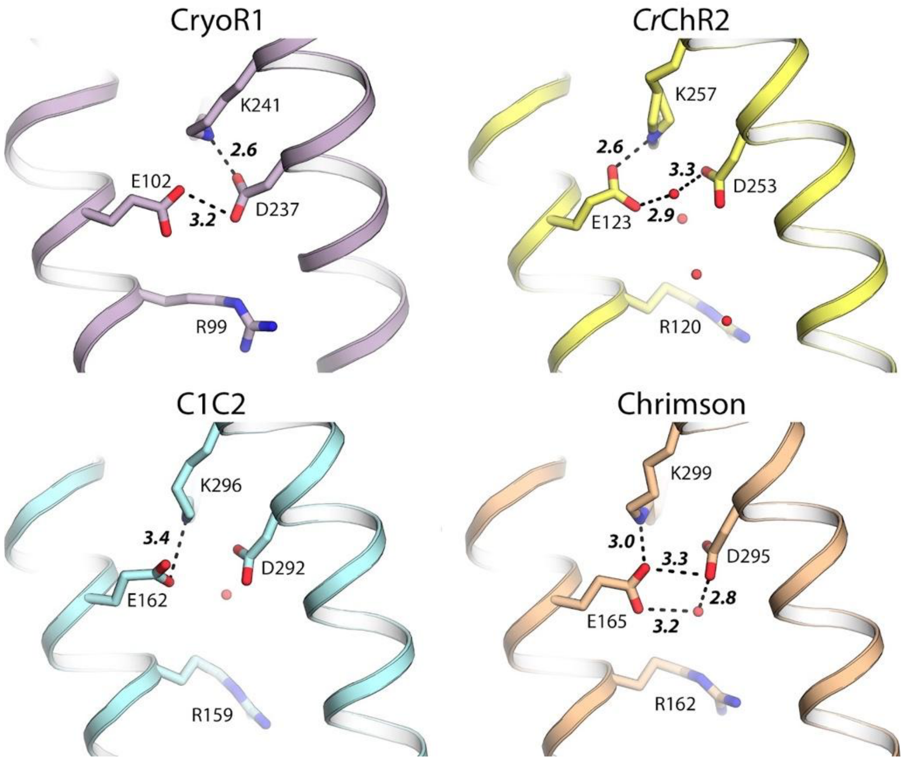

Fig. S18. Comparison of the counterion complexes in CryoRs and channelrhodopsins. CryoR1 (present work) at pH 8.0 is colored purple. *Cr*ChR2 (PDB ID: 6EID) is colored yellow. C1C2 (PDB ID: 3UG9) is colored cyan. Chrimson (PDB ID: 5ZIH) is colored wheat. Hydrogen bonds are shown with black dashed lines. Distances are given in Å.

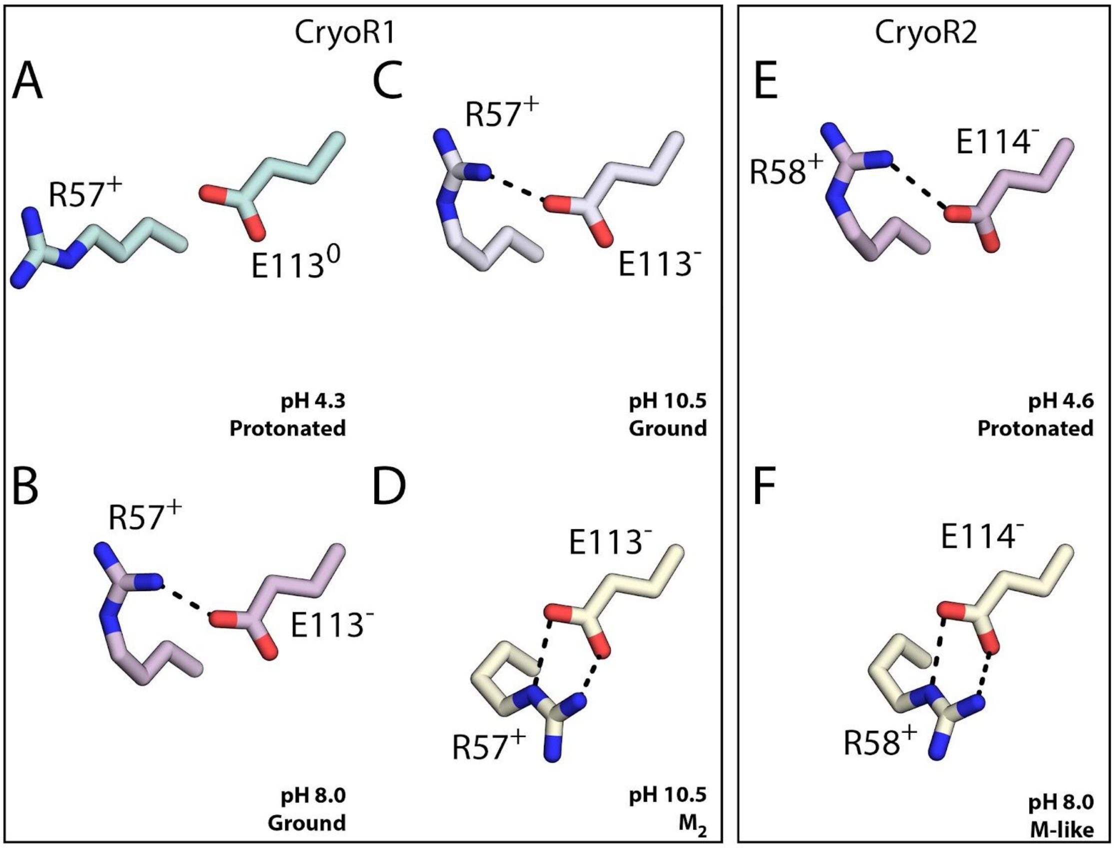

Fig. S19. The arginine-glutamate pair at the cytoplasmic side of CryoRs. A. CryoR1 at pH 4.3. **B.** CryoR1 at pH 8.0. **C.** CryoR1 at pH 10.5 in the ground state. **D.** CryoR1 at pH 10.5 in the M_2 state. **E.** CryoR2 at pH 4.6. **F.** CryoR2 at pH 8.0. H-bonds indicating the salt bridge between the residues are shown with black dashed lines.

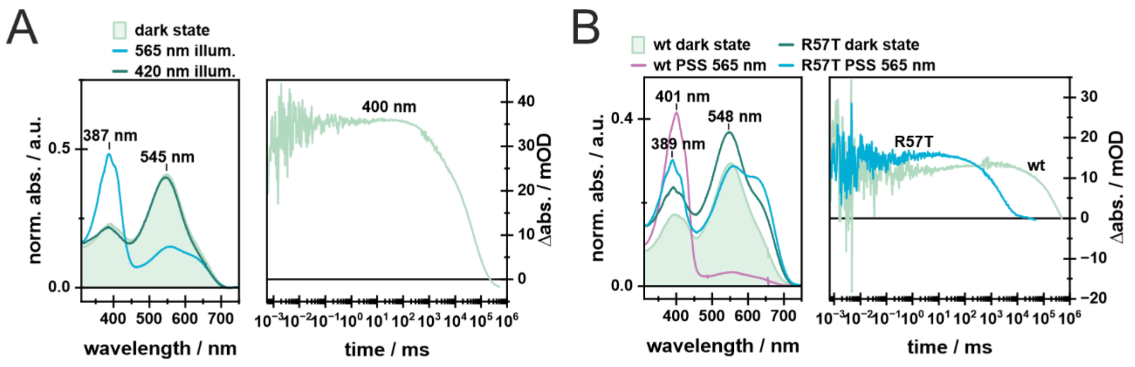

Fig. S20. Illumination experiments of CryoR1 E113Q mutant and comparison of the wt and the R57T mutant. A. Dark state and PSS spectra of CryoR1 E113Q mutant. First, the dark state spectrum was measured, followed by the PSS after illumination of the main absorption band for 100s and the illumination of the potentially rising band for 100s afterward. The 565 nm LED was operated at 1.05 mW and the 420 nm LED was operated at 0.19 mW. Additionally, the flash photolysis transient at 400 nm is shown to elucidate the slow kinetics of the M intermediate and the whole photocycle. **B.** Dark state and PSS spectra of CryoR1 wt and R57T mutant. First of all, the dark state spectrum was measured, followed by the PSS after illumination of the main absorption band for 100s with a 565 nm LED operated at 1.05 mW. Additionally, the respective transient at 400 nm for both proteins is shown to illustrate the accelerated photocycle of the R57T mutant.

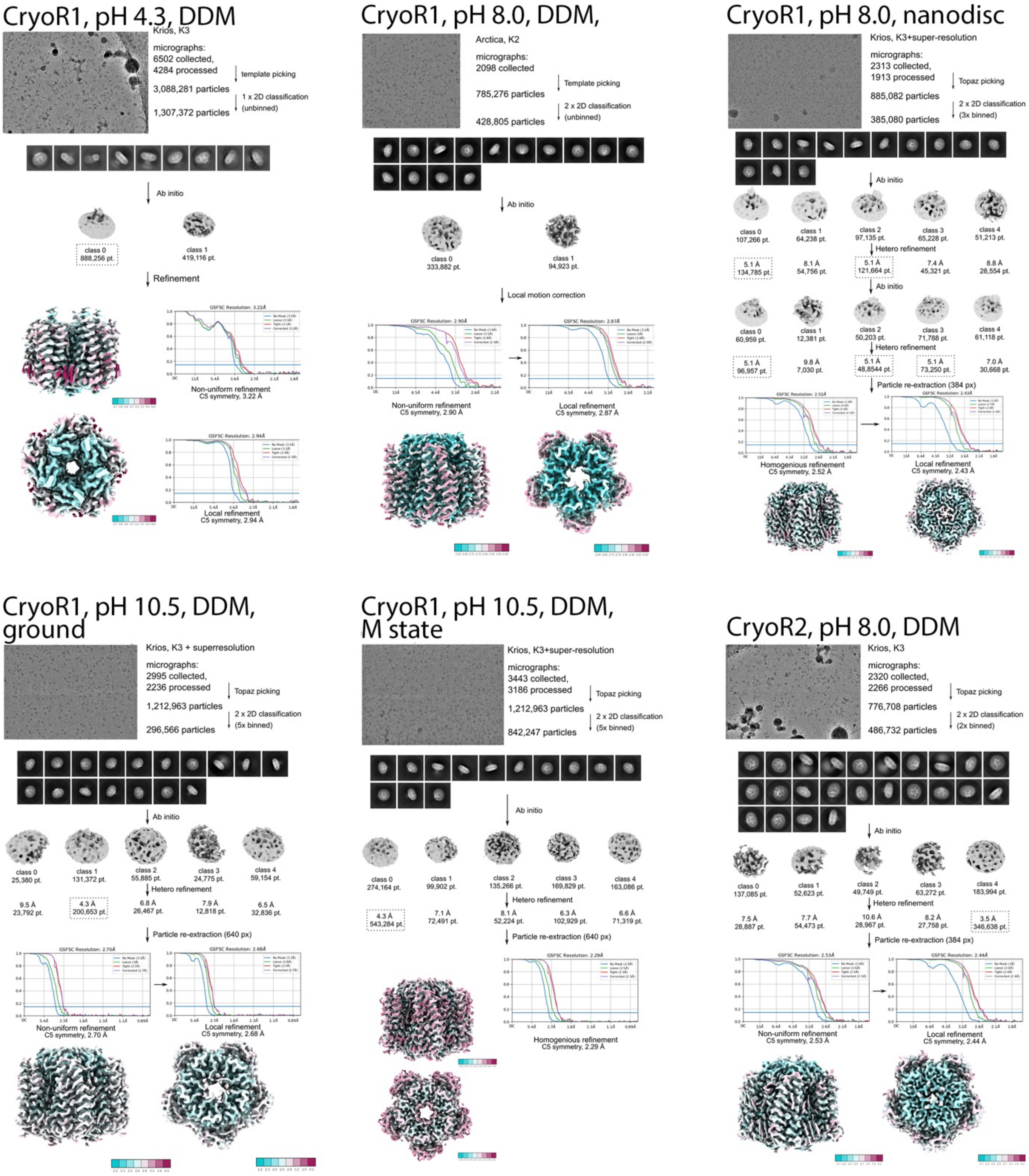

Fig. S21. Workflow for solving the CryoR1 and CryoR2 structures using cryoSPARC. Overall resolution and local resolution are shown. 2D classes and 3D volumes are shown in black squares and gray surfaces, respectively, and appear similar for different samples as they describe the same protein (CryoR1) in 5 of the 6 panels.

Table S1. Source organisms of CryoRhodopsins.

Database, ID	Name	Source organism	Genome Accession (RefSeq)	Environment	#Rhodopsins (including CryoR)
GenBank: WP_166787544	CryoR1	*Cryobacterium levicorallinum* Hh34	GCF_004402405.1	Glacier	3
GenBank: UFS59673.1	CryoR2	*Subtercola endophyticus* AK-R2A1-2	GCF_021044565.1	High mountain, plant	2
GenBank: WP_166791776	CryoR3	*Cryobacterium frigoriphilum* Hh14	GCF_004403305.1	Glacier	1
UniProtKB: A0A4T2CD69	CryoR4	*Subtercola vilae* DB165	GCF_004923255.1	High mountain	3
UniProtKB: A0A3E0VLV3	CryoR5	*Subtercola boreus* DSM 13056	GCF_006716115.1	Boreal groundwater	1
UniProtKB: A0A4R8VLN5		*Cryobacterium algoricola* MDB2-B	GCF_004402485.1	Glacier	2
GenBank: WP_243013333.1		*Cryobacterium zhongshanensis* ZS14-85	GCF_022760445.1	Antarctic soil	2
GenBank: WBM79723.1		*Cryobacterium breve* TMT4-23	GCF_004402375.1	Glacier	2
UniProtKB: A0A4R8WGZ9		*Cryobacterium sp.* MDB2-A-2	GCF_004402425.1	Glacier	2
GenBank: WP_134559116.1		*Cryobacterium sp.* 5I3	GCF_035019585.1	Glacier	2
UniProtKB: A0A924R041		*Microbacteriaceae bacterium* FL-bin-143	NA	Glacier	NA (MAG)
GenBank: WP_259537282		*Herbiconiux daphne* CPCC 203386	GCF_024979295.1	High mountain, plant	1
GenBank: WP_241991064		*Cryobacterium sp.* Hh38	GCF_004403235.1	Glacier	3
UniProtKB: A0A924CVH2		*Microbacteriaceae bacterium* FL-bin-354	NA	Glacier	NA (MAG)
GenBank: WP_255546462		*Glaciihabitans sp.* dw_435	GCF_018449675.1	Glacier	1
GenBank: WP_158594898		*Cryobacterium melibiosiphilum* Hh39	GCF_003602235.1	Glacier	2
UniProtKB: A0A924VRN9		*Microbacteriaceae bacterium* FL-bin-11	NA	Glacier	NA (MAG)
UniProtKB: A0A924VQ96		*Microbacteriaceae bacterium* FL-bin-11	NA	Glacier	NA (MAG)
UniProtKB: A0A7G6Z9A4		*Glaciihabitans sp.* INWT7	GCF_014217685.1	Antarctic soil	2
UniProtKB: A0A923UA93		*Microbacteriaceae bacterium* FL-bin-481	NA	Glacier	NA (MAG)
Genbank: WP_116415267.1		*Subtercola boreus* K300	GCF_003399685.1	Boreal groundwater	1
Genbank: WP_116419744.1		*Subtercola boreus* P28004	GCF_003399545.1	Boreal groundwater	2
Genbank: WP_116415267.1		*Subtercola boreus* DSM 13056	GCF_006716115.1	Boreal groundwater	1
UniProtKB: A0A4T2BXG2		*Subtercola vilae* DB165	GCF_004923255.1	High mountain	3
GenBank: WP_172582322		*Subtercola boreus* P27479	GCF_003399635.1	Boreal groundwater	3

GenBank: WP_172592168		*Subtercola boreus* P27444	GCF_003399625.1	Boreal groundwater	3
GenBank: UFS59725.1		*Subtercola endophyticus AK-R2A1-2*	GCF_021044565.1	High mountain, plant	2
UniProtKB: A0A917EVA4		*Subtercola lobariae* CGMCC 1.12976	GCF_014640255.1	High mountain, plant	1
UniParc: UPI001EF18C93		*Subtercola lobariae* 9583b	GCF_016881145.1	High mountain, plant	1

Table S2. Summary of the structures of CryoRs obtained in the present work.

<table>
<tr><th></th><th>CryoR1</th><th>CryoR2</th></tr>
<tr><td>acidic pH</td><td>pH 4.3, cryo-EM,
2.94 Å, DDM</td><td>pH 5.2, X-ray crystallography,
2.65 Å, crystal</td></tr>
<tr><td rowspan="2">neutral pH</td><td>pH 8.0, cryo-EM, 2.4 3 Å,
nanodisc</td><td rowspan="2">pH 8.0, cryo-EM, 2.44 Å,
DDM</td></tr>
<tr><td>pH 8.0, cryo-EM, 2.9 Å,
DDM</td></tr>
<tr><td rowspan="2">alkaline pH</td><td>pH 10.5, cryo-EM, 2.7 Å,
DDM, dark (ground state)</td><td rowspan="2">-</td></tr>
<tr><td>pH 10.5, cryo-EM, 2.3 Å,
DDM, illuminated (M_2 state)</td></tr>
</table>

Table S3. Cryo-EM data collection, refinement, and validation.

**Protein**			**CryoR1**			**CryoR2**
**pH**	4.3	8.0 (DDM)	8.0 (nanodisc)	10.5	10.5	8.0
**State**	Ground	Ground	Ground	Ground	M_2	M_2
**PDB ID**	8R0K	8R0M	8R0L	8R0N	8R0O	8R0P
**Data collection and processing**						
Voltage (kV)	300	200	300	300	300	300
Electron exposure (e^-/Å^2)	50.0	53.1	50	50	50	50
Defocus range (μm)	−0.9 to −2.7	−0.5 to −2.0	−0.8 to −2.2	−1.2 to −2.2	−1.2 to −2.2	−0.6 to -2.2
Pixel size (Å)	0.836	1.022	0.836	0.418	0.418	0.836
Micrographs collected	6502	2098	2313	2995	3443	2320
Micrographs processed	4284	2098	1913	2236	3186	2266
Symmetry imposed	C5	C5	C5	C5	C5	C5
Initial dataset (# of particles)	3,088,281	785,276	885,082	426,125	1,212,963	776,708
Final dataset (# of particles)	888,256	333,882	218,132	200,277	541,978	329,937
Map resolution (Å) $FSC_{01.43}$	2.94	2.87	2.43	2.70	2.30	2.44
**Refinement**						
Initial model used	Alphafold	Alphafold	Alphafold	Alphafold	Alphafold	Alphafold
Map-sharpening *B* factor (Å^2)	177.2	117.4	79.9	98.1	87.7	84.8
No. atoms						
Protein	9,615	10,553	10,599	10,562	11,069	10,550
Retinal	100	100	100	100	200	100
Lipid	153	387	261	181	165	354
Water	17	55	240	78	343	155
B-factors (Å^2)						
Protein	56.0	35.1	42.1	47.0	34.2	42.5
Retinal	76.8	50.0	51.0	46.2	40.3	30.0
Lipid	75.5	79.3	79.7	79.4	56.3	82.6
Water	45.3	21.4	42.7	43.0	39.8	35.8
R.m.s. deviations						
Bond lengths (Å)	0.0078	0.0095	0.0084	0.0092	0.0099	0.0095
Bond angles (°)	1.3454	1.5921	1.3929	1.3013	1.4568	1.6527
**Validation**						
MolProbity score	1.83	1.85	1.96	1.71	1.41	2.33
Clash score	11.97	6.48	6.82	6.38	7.50	7.64

Poor rotamers (%)	1.36	1.79	4.54	1.88	0.98	5.84
Ramachandran plot (%)						
Favored	97.25	95.67	97.62	97.19	98.63	95.31
Allowed	2.75	4.33	2.38	2.81	1.29	4.26
Outliers	0.00	0.00	0.00	0.00	0.07	0.43
Model to map fit CC	0.86	0.81	0.85	0.86	0.86	0.83

Table S4. X-ray crystallography data collection and structure refinement statistics on CryoR2.

	Type A, dark	Type B, dark	Type B, illuminated
**PDB ID**	8R96	8R97	8R98
**Data collection**			
Space group	C 2 2 2 1	C 1 2 1	C 1 2 1
Cell dimensions			
a, *b*, *c* (Å)	85.6, 295.7, 185.5	149.8, 85.0, 296.4	150.1, 85.0, 296.9
a, b, g (°)	90, 90, 90	90, 96.83, 90	90, 96.86, 90
Resolution (Å)	92.750-2.457 (2.719-2.457)*	74.351-2.718 (3.046-2.718)*	74.387-2.774 (3.100-2.774)*
R_{pim}, %	6.4 (96.2)	8.5 (104.9)	8.5 (106.1)
I / s*I*	8.5 (0.8)	6.1 (0.7)	6.5 (0.7)
Completeness (%)	91.3 (81.5)	84.3 (42.0)	81.5 (46.3)
Redundancy	13.4 (13.6)	6.6 (6.3)	6.9 (6.7)
**Refinement**			
Resolution (Å)	20-2.46	20-3.0	20-3.0
No. reflections	53,453	61,380	54,950
R_{work} / R_{free}	23.4/25.4	26.8/30.4	28.7/30.9
No. atoms			
Protein	10,738	21,408	21,322
Retinal	100	200	200
Lipid	150	295	272
Solvent	71.5	50	50
B-factors (Å^2)			
Protein	63.2	90.5	93.8
Retinal	66.6	94.4	104.9
Lipid	82.7	87.4	58.3
Solvent	56	124.8	135.0
R.m.s. deviations			
Bond lengths (Å)	0.002	0.002	0.002
Bond angles (°)	1.062	1.095	1.069

*Values in parentheses are for the highest-resolution shell.

Data S1. Sequences of CryoRhodopsins found in the course of the present work.

REFERENCES AND NOTES

1. P.-A. Bulzu, V. S. Kavagutti, A.-S. Andrei, R. Ghai, The evolutionary kaleidoscope of rhodopsins. *mSystems* **7**, e0040522 (2022).

2. E. G. Govorunova, O. A. Sineshchekov, H. Li, J. L. Spudich, Microbial rhodopsins: Diversity, mechanisms, and optogenetic Applications. *Annu. Rev. Biochem.* **86**, 845–872 (2017).

3. O. Béjà, L. Aravind, E. V. Koonin, M. T. Suzuki, A. Hadd, L. P. Nguyen, S. B. Jovanovich, C. M. Gates, R. A. Feldman, J. L. Spudich, E. N. Spudich, E. F. DeLong, Bacterial rhodopsin: Evidence for a new type of phototrophy in the sea. *Science* **289**, 1902–1906 (2000).

4. A. Pushkarev, K. Inoue, S. Larom, J. Flores-Uribe, M. Singh, M. Konno, S. Tomida, S. Ito, R. Nakamura, S. P. Tsunoda, A. Philosof, I. Sharon, N. Yutin, E. V. Koonin, H. Kandori, O. Béjà, A distinct abundant group of microbial rhodopsins discovered using functional metagenomics. *Nature* **558**, 595–599 (2018).

5. E. S. Boyden, F. Zhang, E. Bamberg, G. Nagel, K. Deisseroth, Millisecond-timescale, genetically targeted optical control of neural activity. *Nat. Neurosci.* **8**, 1263–1268 (2005).

6. E. G. Govorunova, Y. Gou, O. A. Sineshchekov, H. Li, X. Lu, Y. Wang, L. S. Brown, F. St-Pierre, M. Xue, J. L. Spudich, Kalium channelrhodopsins are natural light-gated potassium channels that mediate optogenetic inhibition. *Nat. Neurosci.* **25**, 967–974 (2022).

7. J. Vierock, E. Peter, C. Grimm, A. Rozenberg, I.-W. Chen, L. Tillert, A. G. Castro Scalise, M. Casini, S. Augustin, D. Tanese, B. C. Forget, R. Peyronnet, F. Schneider-Warme, V. Emiliani, O. Béjà, P. Hegemann, WiChR, a highly potassium-selective channelrhodopsin for low-light one- and two-photon inhibition of excitable cells. *Sci. Adv.* **8**, eadd7729 (2022).

8. E. G. Govorunova, O. A. Sineshchekov, R. Janz, X. Liu, J. L. Spudich, Natural light-gated anion channels: A family of microbial rhodopsins for advanced optogenetics. *Science* **349**, 647–650 (2015).

9. D. Oesterhelt, W. Stoeckenius, Rhodopsin-like protein from the purple membrane of Halobacterium halobium. *Nat. New Biol.* **233**, 149–152 (1971).

10. D. Bratanov, K. Kovalev, J.-P. Machtens, R. Astashkin, I. Chizhov, D. Soloviov, D. Volkov, V. Polovinkin, D. Zabelskii, T. Mager, I. Gushchin, T. Rokitskaya, Y. Antonenko, A. Alekseev, V.

Shevchenko, N. Yutin, R. Rosselli, C. Baeken, V. Borshchevskiy, G. Bourenkov, A. Popov, T. Balandin, G. Büldt, D. J. Manstein, F. Rodriguez-Valera, C. Fahlke, E. Bamberg, E. Koonin, V. Gordeliy, Unique structure and function of viral rhodopsins. *Nat. Commun.* **10**, 4939 (2019).

11. D. Zabelskii, A. Alekseev, K. Kovalev, V. Rankovic, T. Balandin, D. Soloviov, D. Bratanov, E. Savelyeva, E. Podolyak, D. Volkov, S. Vaganova, R. Astashkin, I. Chizhov, N. Yutin, M. Rulev, A. Popov, A.-S. Eria-Oliveira, T. Rokitskaya, T. Mager, Y. Antonenko, R. Rosselli, G. Armeev, K. Shaitan, M. Vivaudou, G. Büldt, A. Rogachev, F. Rodriguez-Valera, M. Kirpichnikov, T. Moser, A. Offenhäusser, D. Willbold, E. Koonin, E. Bamberg, V. Gordeliy, Viral rhodopsins 1 are an unique family of light-gated cation channels. *Nat. Commun.* **11**, 5707 (2020).

12. A. Harris, M. Ljumovic, A.-N. Bondar, Y. Shibata, S. Ito, K. Inoue, H. Kandori, L. S. Brown, A new group of eubacterial light-driven retinal-binding proton pumps with an unusual cytoplasmic proton donor. *Biochim. Biophys. Acta* **1847**, 1518–1529 (2015).

13. I. S. Okhrimenko, K. Kovalev, L. E. Petrovskaya, N. S. Ilyinsky, A. A. Alekseev, E. Marin, T. I. Rokitskaya, Y. N. Antonenko, S. A. Siletsky, P. A. Popov, Y. A. Zagryadskaya, D. V. Soloviov, I. V. Chizhov, D. V. Zabelskii, Y. L. Ryzhykau, A. V. Vlasov, A. I. Kuklin, A. O. Bogorodskiy, A. E. Mikhailov, D. V. Sidorov, S. Bukhalovich, F. Tsybrov, S. Bukhdruker, A. D. Vlasova, V. I. Borshchevskiy, D. A. Dolgikh, M. P. Kirpichnikov, E. Bamberg, V. I. Gordeliy, Mirror proteorhodopsins. *Commun. Chem.* **6**, 88 (2023).

14. M. D. C. Marín, A. L. Jaffe, P. T. West, M. Konno, J. F. Banfield, K. Inoue, Biophysical characterization of microbial rhodopsins with DSE motif. *Biophys. Physicobiol.* **20**, e201023 (2023).

15. L. Jiang, Y. Peng, J. Seo, D. Jeon, M. G. Jo, J. H. Lee, J. C. Jeong, C. Y. Kim, H. C. Park, J. Lee, Subtercola endophyticus sp. nov., a cold-adapted bacterium isolated from Abies koreana. *Sci. Rep.* **12**, 12114 (2022).

16. Q. Liu, L.-L. Yang, Y.-H. Xin, Diversity of the genus *Cryobacterium* and proposal of 19 novel species isolated from glaciers. *Front. Microbiol.* **14**, 1115168 (2023).

17. R. Bar-Shalom, A. Rozenberg, M. Lahyani, B. Hassanzadeh, G. Sahoo, M. Haber, I. Burgsdorf, X. Tang, V. Squatrito, L. Gomez-Consarnau, O. Béjà, L. Steindler, Rhodopsin-mediated nutrient uptake by cultivated photoheterotrophic Verrucomicrobiota. *ISME J.* **17**, 1063–1073 (2023).

18. K. Inoue, M. Karasuyama, R. Nakamura, M. Konno, D. Yamada, K. Mannen, T. Nagata, Y. Inatsu, H. Yawo, K. Yura, O. Béjà, H. Kandori, I. Takeuchi, Exploration of natural red-shifted rhodopsins using a machine learning-based Bayesian experimental design. *Commun. Biol.* **4**, 362 (2021).

19. S. V. Tarlachkov, T. V. Shevchuk, M. D. C. Montero-Calasanz, I. P. Starodumova, Diversity of rhodopsins in cultivated bacteria of the family Geodermatophilaceae associated with non-aquatic environments. *Bioinformatics* **36**, 1668–1672 (2020).

20. M. Shibata, K. Inoue, K. Ikeda, M. Konno, M. Singh, C. Kataoka, R. Abe-Yoshizumi, H. Kandori, T. Uchihashi, Oligomeric states of microbial rhodopsins determined by high-speed atomic force microscopy and circular dichroic spectroscopy. *Sci. Rep.* **8**, 8262 (2018).

21. H. Otto, T. Marti, M. Holz, T. Mogi, M. Lindau, H. G. Khorana, M. P. Heyn, Aspartic acid-96 is the internal proton donor in the reprotonation of the Schiff base of bacteriorhodopsin. *Proc. Natl. Acad. Sci. U.S.A.* **86**, 9228–9232 (1989).

22. S. Subramaniam, D. A. Greenhalgh, H. G. Khorana, Aspartic acid 85 in bacteriorhodopsin functions both as proton acceptor and negative counterion to the Schiff base. *J. Biol. Chem.* **267**, 25730–25733 (1992).

23. V. Shevchenko, T. Mager, K. Kovalev, V. Polovinkin, A. Alekseev, J. Juettner, I. Chizhov, C. Bamann, C. Vavourakis, R. Ghai, I. Gushchin, V. Borshchevskiy, A. Rogachev, I. Melnikov, A. Popov, T. Balandin, F. Rodriguez-Valera, D. J. Manstein, G. Bueldt, E. Bamberg, V. Gordeliy, Inward H+ pump xenorhodopsin: Mechanism and alternative optogenetic approach. *Sci. Adv.* **3**, e1603187 (2017).

24. J. Weissbecker, C. Boumrifak, M. Breyer, T. Wießalla, V. Shevchenko, T. Mager, C. Slavov, A. Alekseev, K. Kovalev, V. Gordeliy, E. Bamberg, J. Wachtveitl, The voltage dependent sidedness of the reprotonation of the retinal Schiff base determines the unique inward pumping of xenorhodopsin. *Angew. Chem. Int. Ed. Engl.* **60**, 23010–23017 (2021).

25. T. Schreckenbach, B. Walckhoff, D. Oesterhelt, Studies on the retinal-protein interaction in bacteriorhodopsin. *Eur. J. Biochem.* **76**, 499–511 (1977).

26. C. E. Eckert, J. Kaur, C. Glaubitz, J. Wachtveitl, Ultrafast photoinduced deactivation dynamics of proteorhodopsin. *J. Phys. Chem. Lett.* **8**, 512–517 (2017).

27. C. Bamann, R. Gueta, S. Kleinlogel, G. Nagel, E. Bamberg, Structural guidance of the photocycle of channelrhodopsin-2 by an interhelical hydrogen bond. *Biochemistry* **49**, 267–278 (2010).

28. P. Hegemann, D. Oesterhelt, E. Bamberg, The transport activity of the light-driven chloride pump halorhodopsin is regulated by green and blue light. *Biochim. Biophys. Acta Biomembr.* **819**, 195–205 (1985).

29. B. Karvaly, Z. Dancsházy, Bacteriorhodopsin: A molecular photoelectric regulator Quenching of photovoltaic effect of bimolecular lipid membranes containing bacteriorhodopsin by blue light. *FEBS Lett.* **76**, 36–40 (1977).

30. J. Y. Jung, A. R. Choi, Y. K. Lee, H. K. Lee, K.-H. Jung, Spectroscopic and photochemical analysis of proteorhodopsin variants from the surface of the Arctic Ocean. *FEBS Lett.* **582**, 1679–1684 (2008).

31. K. Inoue, H. Ono, R. Abe-Yoshizumi, S. Yoshizawa, H. Ito, K. Kogure, H. Kandori, A light-driven sodium ion pump in marine bacteria. *Nat. Commun.* **4**, 1678 (2013).

32. G. Váró, L. S. Brown, M. Lakatos, J. K. Lanyi, Characterization of the photochemical reaction cycle of proteorhodopsin. *Biophys. J.* **84**, 1202–1207 (2003).

33. R. Huber, T. Köhler, M. O. Lenz, E. Bamberg, R. Kalmbach, M. Engelhard, J. Wachtveitl, pH-dependent photoisomerization of retinal in proteorhodopsin. *Biochemistry* **44**, 1800–1806 (2005).

34. M. O. Lenz, A. C. Woerner, C. Glaubitz, J. Wachtveitl, Photoisomerization in proteorhodopsin mutant D97N. *Photochem. Photobiol.* **83**, 226–231 (2007).

35. F. Scholz, E. Bamberg, C. Bamann, J. Wachtveitl, Tuning the primary reaction of channelrhodopsin-2 by imidazole, pH, and site-specific mutations. *Biophys. J.* **102**, 2649–2657 (2012).

36. M. Asido, R. K. Kar, C. N. Kriebel, M. Braun, C. Glaubitz, I. Schapiro, J. Wachtveitl, Transient near-UV absorption of the light-driven sodium pump *Krokinobacter eikastus* rhodopsin 2: A spectroscopic marker for retinal configuration. *J. Phys. Chem. Lett.* **12**, 6284–6291 (2021).

37. S. Tahara, S. Takeuchi, R. Abe-Yoshizumi, K. Inoue, H. Ohtani, H. Kandori, T. Tahara, Origin of the reactive and nonreactive excited states in the primary reaction of rhodopsins: pH dependence of femtosecond absorption of light-driven sodium ion pump rhodopsin KR2. *J. Phys. Chem. B* **122**, 4784–4792 (2018).

38. C.-F. Chang, H. Kuramochi, M. Singh, R. Abe-Yoshizumi, T. Tsukuda, H. Kandori, T. Tahara, A unified view on varied ultrafast dynamics of the primary process in microbial rhodopsins. *Angew. Chem. Int. Ed. Engl.* **61**, e202111930 (2022).

39. Q. Wang, R. W. Schoenlein, L. A. Peteanu, R. A. Mathies, C. V. Shank, Vibrationally coherent photochemistry in the femtosecond primary event of vision. *Science* **266**, 422–424 (1994).

40. J. Briand, O. Bräm, J. Réhault, J. Léonard, A. Cannizzo, M. Chergui, V. Zanirato, M. Olivucci, J. Helbing, S. Haacke, Coherent ultrafast torsional motion and isomerization of a biomimetic dipolar photoswitch. *Phys. Chem. Chem. Phys.* **12**, 3178–3187 (2010).

41. E. S. Imasheva, S. P. Balashov, J. M. Wang, A. K. Dioumaev, J. K. Lanyi, Selectivity of retinal photoisomerization in proteorhodopsin is controlled by aspartic acid 227. *Biochemistry* **43**, 1648–1655 (2004).

42. E. S. Imasheva, K. Shimono, S. P. Balashov, J. M. Wang, U. Zadok, M. Sheves, N. Kamo, J. K. Lanyi, Formation of a long-lived photoproduct with a deprotonated Schiff base in proteorhodopsin, and its enhancement by mutation of Asp227. *Biochemistry* **44**, 10828–10838 (2005).

43. J. Herz, M.-K. Verhoefen, I. Weber, C. Bamann, C. Glaubitz, J. Wachtveitl, Critical role of Asp227 in the photocycle of proteorhodopsin. *Biochemistry* **51**, 5589–5600 (2012).

44. K. Yamada, A. Kawanabe, S. Yoshizawa, K. Inoue, K. Kogure, H. Kandori, Anomalous pH Effect of blue proteorhodopsin. *J. Phys. Chem. Lett.* **3**, 800–804 (2012).

45. A. Rozenberg, I. Kaczmarczyk, D. Matzov, J. Vierock, T. Nagata, M. Sugiura, K. Katayama, Y. Kawasaki, M. Konno, Y. Nagasaka, M. Aoyama, I. Das, E. Pahima, J. Church, S. Adam, V. A. Borin, A. Chazan, S. Augustin, J. Wietek, J. Dine, Y. Peleg, A. Kawanabe, Y. Fujiwara, O. Yizhar, M. Sheves, I. Schapiro, Y. Furutani, H. Kandori, K. Inoue, P. Hegemann, O. Béjà, M. Shalev-Benami, Rhodopsin-bestrophin fusion proteins from unicellular algae form gigantic pentameric ion channels. *Nat. Struct. Mol. Biol.* **29**, 592–603 (2022).

46. H. Luecke, B. Schobert, H. T. Richter, J. P. Cartailler, J. K. Lanyi, Structure of bacteriorhodopsin at 1.55 Å resolution. *J. Mol. Biol.* **291**, 899–911 (1999).

47. V. Borshchevskiy, K. Kovalev, E. Round, R. Efremov, R. Astashkin, G. Bourenkov, D. Bratanov, T. Balandin, I. Chizhov, C. Baeken, I. Gushchin, A. Kuzmin, A. Alekseev, A. Rogachev, D. Willbold, M. Engelhard, E. Bamberg, G. Büldt, V. Gordeliy, True-atomic-resolution insights into the structure and functional role of linear chains and low-barrier hydrogen bonds in proteins. *Nat. Struct. Mol. Biol.* **29**, 440–450 (2022).

48. T. Ikuta, W. Shihoya, M. Sugiura, K. Yoshida, M. Watari, T. Tokano, K. Yamashita, K. Katayama, S. P. Tsunoda, T. Uchihashi, H. Kandori, O. Nureki, Structural insights into the mechanism of rhodopsin phosphodiesterase. *Nat. Commun.* **11**, 5605 (2020).

49. K. Kovalev, V. Polovinkin, I. Gushchin, A. Alekseev, V. Shevchenko, V. Borshchevskiy, R. Astashkin, T. Balandin, D. Bratanov, S. Vaganova, A. Popov, V. Chupin, G. Büldt, E. Bamberg, V. Gordeliy, Structure and mechanisms of sodium-pumping KR2 rhodopsin. *Sci. Adv.* **5**, eaav2671 (2019).

50. K. Kovalev, R. Astashkin, I. Gushchin, P. Orekhov, D. Volkov, E. Zinovev, E. Marin, M. Rulev, A. Alekseev, A. Royant, P. Carpentier, S. Vaganova, D. Zabelskii, C. Baeken, I. Sergeev, T. Balandin, G. Bourenkov, X. Carpena, R. Boer, N. Maliar, V. Borshchevskiy, G. Büldt, E. Bamberg, V. Gordeliy, Molecular mechanism of light-driven sodium pumping. *Nat. Commun.* **11**, 2137 (2020).

51. E. Podoliak, G. H. U. Lamm, A. Alekseev, E. Marin, A. V. Schellbach, D. A. Fedotov, A. Stetsenko, N. Maliar, G. Bourenkov, T. Balandin, C. Baeken, R. Astashkin, T. R. Schneider, A. Bateman, J. Wachtveitl, I. Schapiro, V. Busskamp, A. Guskov, V. Gordeliy, K. Kovalev, A novel subgroup of light-driven sodium pumps with an additional Schiff base counterion. bioRxiv 561842 [Preprint] (2023). https://doi.org/10.1101/2023.10.11.561842.

52. K. Kovalev, F. Tsybrov, A. Alekseev, V. Shevchenko, D. Soloviov, S. Siletsky, G. Bourenkov, M. Agthe, M. Nikolova, D. von Stetten, R. Astashkin, S. Bukhdruker, I. Chizhov, A. Royant, A. Kuzmin, I. Gushchin, R. Rosselli, F. Rodriguez-Valera, N. Ilyinskiy, A. Rogachev, V. Borshchevskiy, T. R. Schneider, E. Bamberg, V. Gordeliy, Mechanisms of inward transmembrane proton translocation. *Nat. Struct. Mol. Biol.* **30**, 970–979 (2023).

53. T. Weinert, P. Skopintsev, D. James, F. Dworkowski, E. Panepucci, D. Kekilli, A. Furrer, S. Brünle, S. Mous, D. Ozerov, P. Nogly, M. Wang, J. Standfuss, Proton uptake mechanism in bacteriorhodopsin captured by serial synchrotron crystallography. *Science* **365**, 61–65 (2019).

54. I. Gushchin, A. Reshetnyak, V. Borshchevskiy, A. Ishchenko, E. Round, S. Grudinin, M. Engelhard, G. Büldt, V. Gordeliy, Active state of sensory rhodopsin II: Structural determinants for signal transfer and proton pumping. *J. Mol. Biol.* **412**, 591–600 (2011).

55. R. Efremov, V. I. Gordeliy, J. Heberle, G. Büldt, Time-resolved microspectroscopy on a single crystal of bacteriorhodopsin reveals lattice-induced differences in the photocycle kinetics. *Biophys. J.* **91**, 1441–1451 (2006).

56. T. Friedrich, S. Geibel, R. Kalmbach, I. Chizhov, M. Engelhard, E. Bamberg, Proteorhodopsin, a bR homolog from marine bacteria, acts as a light-driven, bidirectional proton pump. *Biophys. J.* 228a (2002).

57. O. Volkov, K. Kovalev, V. Polovinkin, V. Borshchevskiy, C. Bamann, R. Astashkin, E. Marin, A. Popov, T. Balandin, D. Willbold, G. Büldt, E. Bamberg, V. Gordeliy, Structural insights into ion conduction by channelrhodopsin 2. *Science* **358**, eaan8862 (2017).

58. H. E. Kato, F. Zhang, O. Yizhar, C. Ramakrishnan, T. Nishizawa, K. Hirata, J. Ito, Y. Aita, T. Tsukazaki, S. Hayashi, P. Hegemann, A. D. Maturana, R. Ishitani, K. Deisseroth, O. Nureki, Crystal structure of the channelrhodopsin light-gated cation channel. *Nature* **482**, 369–374 (2012).

59. K. Oda, J. Vierock, S. Oishi, S. Rodriguez-Rozada, R. Taniguchi, K. Yamashita, J. S. Wiegert, T. Nishizawa, P. Hegemann, O. Nureki, Crystal structure of the red light-activated channelrhodopsin Chrimson. *Nat. Commun.* **9**, 3949 (2018).

60. C. Bamann, E. Bamberg, J. Wachtveitl, C. Glaubitz, Proteorhodopsin. *Biochim. Biophys. Acta Bioenerg.* **1837**, 614–625 (2014).

61. T. Friedrich, S. Geibel, R. Kalmbach, I. Chizhov, K. Ataka, J. Heberle, M. Engelhard, E. Bamberg, Proteorhodopsin is a light-driven proton pump with variable vectoriality. *J. Mol. Biol.* **321**, 821–838 (2002).

62. J. Mao, N.-N. Do, F. Scholz, L. Reggie, M. Mehler, A. Lakatos, Y.-S. Ong, S. J. Ullrich, L. J. Brown, R. C. D. Brown, J. Becker-Baldus, J. Wachtveitl, C. Glaubitz, Structural basis of the green–blue color switching in proteorhodopsin as determined by NMR spectroscopy. *J. Am. Chem. Soc.* **136**, 17578–17590 (2014).

63. J. Abramson, J. Adler, J. Dunger, R. Evans, T. Green, A. Pritzel, O. Ronneberger, L. Willmore, A. J. Ballard, J. Bambrick, S. W. Bodenstein, D. A. Evans, C.-C. Hung, M. O'Neill, D. Reiman, K. Tunyasuvunakool, Z. Wu, A. Žemgulytė, E. Arvaniti, C. Beattie, O. Bertolli, A. Bridgland, A. Cherepanov, M. Congreve, A. I. Cowen-Rivers, A. Cowie, M. Figurnov, F. B. Fuchs, H. Gladman, R. Jain, Y. A. Khan, C. M. R. Low, K. Perlin, A. Potapenko, P. Savy, S. Singh, A. Stecula, A. Thillaisundaram, C. Tong, S. Yakneen, E. D. Zhong, M. Zielinski, A. Žídek, V. Bapst, P. Kohli, M. Jaderberg, D. Hassabis, J. M. Jumper, Accurate structure prediction of biomolecular interactions with AlphaFold 3. *Nature* **630**, 493–500 (2024).

64. Q. Xu, A. Bateman, R. D. Finn, P. Abdubek, T. Astakhova, H. L. Axelrod, C. Bakolitsa, D. Carlton, C. Chen, H.-J. Chiu, M. Chiu, T. Clayton, D. Das, M. C. Deller, L. Duan, K. Ellrott, D. Ernst, C. L. Farr, J. Feuerhelm, J. C. Grant, A. Grzechnik, G. W. Han, L. Jaroszewski, K. K. Jin, H. E. Klock, M. W. Knuth, P. Kozbial, S. S. Krishna, A. Kumar, D. Marciano, D. McMullan, M. D. Miller, A. T. Morse, E. Nigoghossian, A. Nopakun, L. Okach, C. Puckett, R. Reyes, C. L. Rife, N. Sefcovic, H. J. Tien, C. B. Trame, H. van den Bedem, D. Weekes, T. Wooten, K. O. Hodgson, J. Wooley, M.-A. Elsliger, A. M. Deacon, A. Godzik, S. A. Lesley, I. A. Wilson, Bacterial pleckstrin homology domains: A prokaryotic origin for the PH domain. *J. Mol. Biol.* **396**, 31–46 (2010).

65. A. B. Druzhko, Some aspects of using the fundamental properties of bacteriorhodopsin for recording, processing, and storage of optical information. *J. Photochem. Photobiol. C Photochem. Rev.* **56**, 100620 (2023).

66. A. Lewis, Z. Chen, H. Takei, Optical information storage on a bacteriorhodopsin—Containing film (1995). https://patents.google.com/patent/US5470690A/en.

67. 陈烽, 杨青, Bacteriorhodopsin optical information storage method (2003). https://patents.google.com/patent/CN1112680C/en.

68. S. Redfield, T. Harvey, Branch photocycle technique for holographic recording in bacteriorhodopsin (2006). https://patents.google.com/patent/US20060187795A1/en?oq=US20060187795A1.

69. R. R. Birge, R. Rangarajan, K. N. McCleary, Bacteriorhodopsin protein variants and methods of use for long term data storage (2014). https://patents.google.com/patent/US8883719B2/en.

70. T. Juchem, N. Hampp, Interferometric system for non-destructive testing based on large diameter bacteriorhodopsin films. *Opt. Lasers Eng.* **34**, 87–100 (2000).

71. N. L. Wagner, J. A. Greco, M. J. Ranaghan, R. R. Birge, Directed evolution of bacteriorhodopsin for applications in bioelectronics. *J. R. Soc. Interface* **10**, 20130197–20130197 (2013).

72. G. Varo, J. K. Lanyi, Kinetic and spectroscopic evidence for an irreversible step between deprotonation and reprotonation of the Schiff base in the bacteriorhodopsin photocycle. *Biochemistry* **30**, 5008–5015 (1991).

73. S. Y. Liu, T. G. Ebrey, The quantum efficiency for the interphotoconversion of the blue and pink forms of purple membrane. *Photochem. Photobiol.* **46**, 263–267 (1987).

74. S. Kleinlogel, U. Terpitz, B. Legrum, D. Gökbuget, E. S. Boyden, C. Bamann, P. G. Wood, E. Bamberg, A gene-fusion strategy for stoichiometric and co-localized expression of light-gated membrane proteins. *Nat. Methods* **8**, 1083–1088 (2011).

75. J. Vierock, S. Rodriguez-Rozada, A. Dieter, F. Pieper, R. Sims, F. Tenedini, A. C. F. Bergs, I. Bendifallah, F. Zhou, N. Zeitzschel, J. Ahlbeck, S. Augustin, K. Sauter, E. Papagiakoumou, A. Gottschalk, P. Soba, V. Emiliani, A. K. Engel, P. Hegemann, J. S. Wiegert, BiPOLES is an optogenetic tool developed for bidirectional dual-color control of neurons. *Nat. Commun.* **12**, 4527 (2021).

76. R. C. Edgar, MUSCLE: Multiple sequence alignment with high accuracy and high throughput. *Nucleic Acids Res.* **32**, 1792–1797 (2004).

77. J. Trifinopoulos, L.-T. Nguyen, A. von Haeseler, B. Q. Minh, W-IQ-TREE: A fast online phylogenetic tool for maximum likelihood analysis. *Nucleic Acids Res.* **44**, W232–235 (2016).

78. I. Letunic, P. Bork, Interactive Tree Of Life (iTOL) v5: An online tool for phylogenetic tree display and annotation. *Nucleic Acids Res.* **49**, W293–W296 (2021).

79. K. Katoh, D. M. Standley, MAFFT multiple sequence alignment software version 7: Improvements in performance and usability. *Mol. Biol. Evol.* **30**, 772–780 (2013).

80. L. Fu, B. Niu, Z. Zhu, S. Wu, W. Li, CD-HIT: Accelerated for clustering the next-generation sequencing data. *Bioinformatics* **28**, 3150–3152 (2012).

81. D. Hyatt, G.-L. Chen, P. F. LoCascio, M. L. Land, F. W. Larimer, L. J. Hauser, Prodigal: Prokaryotic gene recognition and translation initiation site identification. *BMC Bioinformatics* **11**, 119 (2010).

82. B. Buchfink, C. Xie, D. H. Huson, Fast and sensitive protein alignment using DIAMOND. *Nat. Methods* **12**, 59–60 (2015).

83. R. L. Tatusov, D. A. Natale, I. V. Garkavtsev, T. A. Tatusova, U. T. Shankavaram, B. S. Rao, B. Kiryutin, M. Y. Galperin, N. D. Fedorova, E. V. Koonin, The COG database: New developments in phylogenetic classification of proteins from complete genomes. *Nucleic Acids Res.* **29**, 22–28 (2001).

84. D. H. Haft, B. J. Loftus, D. L. Richardson, F. Yang, J. A. Eisen, I. T. Paulsen, O. White, TIGRFAMs: A protein family resource for the functional identification of proteins. *Nucleic Acids Res.* **29**, 41–43 (2001).

85. R. D. Finn, J. Clements, S. R. Eddy, HMMER web server: Interactive sequence similarity searching. *Nucleic Acids Res.* **39**, W29–W37 (2011).

86. S. Hunter, R. Apweiler, T. K. Attwood, A. Bairoch, A. Bateman, D. Binns, P. Bork, U. Das, L. Daugherty, L. Duquenne, R. D. Finn, J. Gough, D. Haft, N. Hulo, D. Kahn, E. Kelly, A. Laugraud, I. Letunic, D. Lonsdale, R. Lopez, M. Madera, J. Maslen, C. McAnulla, J. McDowall, J. Mistry, A. Mitchell, N. Mulder, D. Natale, C. Orengo, A. F. Quinn, J. D. Selengut, C. J. A. Sigrist, M. Thimma, P. D. Thomas, F. Valentin, D. Wilson, C. H. Wu, C. Yeats, InterPro: The integrative protein signature database. *Nucleic Acids Res.* **37**, D211–D215 (2009).

87. J. Hallgren, K. Tsirigos, M. D. Pedersen, J. J. Almagro Armenteros, P. Marcatili, H. Nielsen, A. Krogh, O. Winther, DeepTMHMM predicts alpha and beta transmembrane proteins using deep neural network. bioRxiv 487609 [Preprint] (2022). https://doi.org/10.1101/2022.04.08.487609.

88. F. Teufel, J. J. Almagro Armenteros, A. R. Johansen, M. H. Gíslason, S. I. Pihl, K. D. Tsirigos, O. Winther, S. Brunak, G. von Heijne, H. Nielsen, SignalP 6.0 predicts all five types of signal peptides using protein language models. *Nat. Biotechnol.* **40**, 1023–1025 (2022).

89. C. Grimm, A. Silapetere, A. Vogt, Y. A. Bernal Sierra, P. Hegemann, Electrical properties, substrate specificity and optogenetic potential of the engineered light-driven sodium pump eKR2. *Sci. Rep.* **8**, 9316 (2018).

90. F. W. Studier, Protein production by auto-induction in high-density shaking cultures. *Protein Expr. Purif.* **41**, 207–234 (2005).

91. V. Arkhipova, A. Guskov, D. J. Slotboom, Structural ensemble of a glutamate transporter homologue in lipid nanodisc environment. *Nat. Commun.* **11**, 998 (2020).

92. F. Schweighöfer, J. Moreno, S. Bobone, S. Chiantia, A. Herrmann, S. Hecht, J. Wachtveitl, Connectivity pattern modifies excited state relaxation dynamics of fluorophore–photoswitch molecular dyads. *Phys. Chem. Chem. Phys.* **19**, 4010–4018 (2017).

93. C. Slavov, H. Hartmann, J. Wachtveitl, Implementation and evaluation of data analysis strategies for time-resolved optical spectroscopy. *Anal. Chem.* **87**, 2328–2336 (2015).

94. A. Punjani, J. L. Rubinstein, D. J. Fleet, M. A. Brubaker, cryoSPARC: Algorithms for rapid unsupervised cryo-EM structure determination. *Nat. Methods* **14**, 290–296 (2017).

95. T. Bepler, A. Morin, M. Rapp, J. Brasch, L. Shapiro, A. J. Noble, B. Berger, Positive-unlabeled convolutional neural networks for particle picking in cryo-electron micrographs. *Nat. Methods* **16**, 1153–1160 (2019).

96. J. Jumper, R. Evans, A. Pritzel, T. Green, M. Figurnov, O. Ronneberger, K. Tunyasuvunakool, R. Bates, A. Žídek, A. Potapenko, A. Bridgland, C. Meyer, S. A. A. Kohl, A. J. Ballard, A. Cowie, B. Romera-Paredes, S. Nikolov, R. Jain, J. Adler, T. Back, S. Petersen, D. Reiman, E. Clancy, M. Zielinski, M. Steinegger, M. Pacholska, T. Berghammer, S. Bodenstein, D. Silver, O. Vinyals, A. W. Senior, K. Kavukcuoglu, P. Kohli, D. Hassabis, Highly accurate protein structure prediction with AlphaFold. *Nature* **596**, 583–589 (2021).

97. P. V. Afonine, B. K. Poon, R. J. Read, O. V. Sobolev, T. C. Terwilliger, A. Urzhumtsev, P. D. Adams, Real-space refinement in PHENIX for cryo-EM and crystallography. *Acta Crystallogr. D Struct. Biol.* **74**, 531–544 (2018).

98. P. V. Afonine, B. P. Klaholz, N. W. Moriarty, B. K. Poon, O. V. Sobolev, T. C. Terwilliger, P. D. Adams, A. Urzhumtsev, New tools for the analysis and validation of cryo-EM maps and atomic models. *Acta Crystallogr. D Struct. Biol.* **74**, 814–840 (2018).

99. P. Emsley, B. Lohkamp, W. G. Scott, K. Cowtan, Features and development of Coot. *Acta Crystallogr. D Biol. Crystallogr.* **66**, 486–501 (2010).

100. T. D. Goddard, C. C. Huang, E. C. Meng, E. F. Pettersen, G. S. Couch, J. H. Morris, T. E. Ferrin, UCSF ChimeraX: Meeting modern challenges in visualization and analysis. *Protein Sci.* **27**, 14–25 (2018).

101. E. F. Pettersen, T. D. Goddard, C. C. Huang, G. S. Couch, D. M. Greenblatt, E. C. Meng, T. E. Ferrin, UCSF Chimera—A visualization system for exploratory research and analysis. *J. Comput. Chem.* **25**, 1605–1612 (2004).

102. E. M. Landau, J. P. Rosenbusch, Lipidic cubic phases: A novel concept for the crystallization of membrane proteins. *Proc. Natl. Acad. Sci. U.S.A.* **93**, 14532–14535 (1996).

103. D. von Stetten, T. Giraud, P. Carpentier, F. Sever, M. Terrien, F. Dobias, D. H. Juers, D. Flot, C. Mueller-Dieckmann, G. A. Leonard, D. de Sanctis, A. Royant, In crystallo optical spectroscopy (icOS) as a complementary tool on the macromolecular crystallography beamlines of the ESRF. *Acta Crystallogr. D Biol. Crystallogr.* **71**, 15–26 (2015).

104. S. Engilberge, N. Caramello, S. Bukhdruker, M. Byrdin, T. Giraud, P. Jacquet, D. Scortani, R. Biv, H. Gonzalez, A. Broquet, P. van der Linden, S. L. Rose, D. Flot, T. Balandin, V. Gordeliy, J. M. Lahey-Rudolph, M. Roessle, D. de Sanctis, G. A. Leonard, C. Mueller-Dieckmann, A. Royant, The TR-icOS setup at the ESRF: Time-resolved microsecond UV–Vis absorption spectroscopy on protein crystals. *Acta Crystallogr. Sect. Struct. Biol.* **80**, 16–25 (2024).

105. W. Kabsch, XDS. *Acta Crystallogr. D Biol. Crystallogr.* **66**, 125–132 (2010).

106. I. J. Tickle, C. Flensburg, P. Keller, W. Paciorek, A. Sharff, C. Vonrhein, G. Bricogne, *STARANISO* (2018).

107. A. Vagin, A. Teplyakov, Molecular replacement with MOLREP. *Acta Crystallogr. D Biol. Crystallogr.* **66**, 22–25 (2010).

108. M. D. Winn, C. C. Ballard, K. D. Cowtan, E. J. Dodson, P. Emsley, P. R. Evans, R. M. Keegan, E. B. Krissinel, A. G. W. Leslie, A. McCoy, S. J. McNicholas, G. N. Murshudov, N. S. Pannu, E. A.

Potterton, H. R. Powell, R. J. Read, A. Vagin, K. S. Wilson, Overview of the CCP4 suite and current developments. *Acta Crystallogr. D Biol. Crystallogr.* **67**, 235–242 (2011).

109. G. N. Murshudov, P. Skubák, A. A. Lebedev, N. S. Pannu, R. A. Steiner, R. A. Nicholls, M. D. Winn, F. Long, A. A. Vagin, REFMAC5 for the refinement of macromolecular crystal structures. *Acta Crystallogr. D Biol. Crystallogr.* **67**, 355–367 (2011).

110. P. Emsley, K. Cowtan, Coot: Model-building tools for molecular graphics. *Acta Crystallogr. D Biol. Crystallogr.* **60**, 2126–2132 (2004).

111. K. Edman, P. Nollert, A. Royant, H. Belrhali, E. Pebay-Peyroula, J. Hajdu, R. Neutze, E. M. Landau, High-resolution x-ray structure of an early intermediate in the bacteriorhodopsin photocycle. *Nature* **401**, 822–826 (1999).

112. K. Kondo, R. Ohtake, S. Nakano, M. Terashima, H. Kojima, M. Fukui, M. Demura, T. Kikukawa, T. Tsukamoto, Contribution of proteorhodopsin to light-dependent biological responses in hymenobacter nivis P3T isolated from red snow in Antarctica. *Biochemistry* **63**, 2257–2265 (2024).

113. K. Kojima, T. Ueta, T. Noji, K. Saito, K. Kanehara, S. Yoshizawa, H. Ishikita, Y. Sudo, Vectorial proton transport mechanism of RxR, a phylogenetically distinct and thermally stable microbial rhodopsin. *Sci. Rep.* **10**, 282 (2020).

114. D. Zabelskii, N. Dmitrieva, O. Volkov, V. Shevchenko, K. Kovalev, T. Balandin, D. Soloviov, R. Astashkin, E. Zinovev, A. Alekseev, E. Round, V. Polovinkin, I. Chizhov, A. Rogachev, I. Okhrimenko, V. Borshchevskiy, V. Chupin, G. Büldt, N. Yutin, E. Bamberg, E. Koonin, V. Gordeliy, Structure-based insights into evolution of rhodopsins. *Commun. Biol.* **4**, 821 (2021).

115. N. Maliar, I. S. Okhrimenko, L. E. Petrovskaya, A. A. Alekseev, K. V. Kovalev, D. V. Soloviov, P. A. Popov, T. I. Rokitskaya, Y. N. Antonenko, D. V. Zabelskii, D. A. Dolgikh, M. P. Kirpichnikov, V. I. Gordeliy, Novel pH-sensitive microbial rhodopsin from Sphingomonas paucimobilis. *Dokl. Biochem. Biophys.* **495**, 342–346 (2020).

116. K. Inoue, S. Tahara, Y. Kato, S. Takeuchi, T. Tahara, H. Kandori, Spectroscopic study of proton-transfer mechanism of inward proton-pump rhodopsin, Parvularcula oceani xenorhodopsin. *J. Phys. Chem. B* **122**, 6453–6461 (2018).

117. K. Hayashi, M. Mizuno, H. Kandori, Y. Mizutani, Cis–trans reisomerization precedes reprotonation of the retinal chromophore in the photocycle of schizorhodopsin 4. *Angew. Chem. Int. Ed. Engl.* **134**, e202203149 (2022).

118. K. Inoue, S. P. Tsunoda, M. Singh, S. Tomida, S. Hososhima, M. Konno, R. Nakamura, H. Watanabe, P.-A. Bulzu, H. L. Banciu, A.-Ş. Andrei, T. Uchihashi, R. Ghai, O. Béjà, H. Kandori, Schizorhodopsins: A family of rhodopsins from Asgard archaea that function as light-driven inward H^+ pumps. *Sci. Adv.* **6**, eaaz2441 (2020).

119. J. Kuhne, J. Vierock, S. A. Tennigkeit, M. A. Dreier, J. Wietek, D. Petersen, K. Gavriljuk, S. F. El-Mashtoly, P. Hegemann, K. Gerwert, Unifying photocycle model for light adaptation and temporal evolution of cation conductance in channelrhodopsin-2. *Proc. Natl. Acad. Sci. U.S.A.* **116**, 9380–9389 (2019).

120. Y. Sudo, M. Mizuno, Z. Wei, S. Takeuchi, T. Tahara, Y. Mizutani, The early steps in the photocycle of a photosensor protein sensory rhodopsin I from Salinibacter ruber. *J. Phys. Chem. B* **118**, 1510–1518 (2014).

121. O. A. Sineshchekov, H. Li, E. G. Govorunova, J. L. Spudich, Photochemical reaction cycle transitions during anion channelrhodopsin gating. *Proc. Natl. Acad. Sci. U.S.A.* **113**, E1993–E2000 (2016).

122. G. Dai, X. Geng, Chaoluomeng, J. Tamogami, T. Kikukawa, M. Demura, N. Kamo, T. Iwasa, Photocycle of sensory rhodopsin II from halobacterium salinarum (HsSRII): Mutation of D103 accelerates M decay and changes the decay pathway of a 13-cis O-like species. *Photochem. Photobiol.* **94**, 705–714 (2018).

123. J. P. Klare, G. Schmies, I. Chizhov, K. Shimono, N. Kamo, M. Engelhard, Probing the proton channel and the retinal binding site of Natronobacterium pharaonis sensory rhodopsin II. *Biophys. J.* **82**, 2156–2164 (2002).

124. M. Iwamoto, K. Shimono, M. Sumi, N. Kamo, Positioning proton-donating residues to the Schiff-base accelerates the M-decay of *pharaonis* phoborhodopsin expressed in *Escherichia coli*. *Biophys. Chem.* **79**, 187–192 (1999).

125. O. A. Sineshchekov, V. D. Trivedi, J. Sasaki, J. L. Spudich, Photochromicity of Anabaena sensory rhodopsin, an atypical microbial receptor with a cis-retinal light-adapted form. *J. Biol. Chem.* **280**, 14663–14668 (2005).

126. M. Broser, W. Busse, A. Spreen, M. Reh, Y. A. Bernal Sierra, S. Hwang, T. Utesch, H. Sun, P. Hegemann, Diversity of rhodopsin cyclases in zoospore-forming fungi. *Proc. Natl. Acad. Sci. U.S.A.* **120**, e2310600120 (2023).

127. K.-H. Jung, V. D. Trivedi, J. L. Spudich, Demonstration of a sensory rhodopsin in eubacteria. *Mol. Microbiol.* **47**, 1513–1522 (2003).

128. T. Nakamura, M. Singh, M. Sugiura, S. Kato, R. Yamamoto, H. Kandori, Y. Furutani, SNap Bond, a crucial hydrogen bond between Ser in helix 3 and Asn in helix 4, regulates the structural dynamics of heliorhodopsin. *J. Mol. Biol.* **436**, 168666 (2024).

129. K. Kovalev, D. Volkov, R. Astashkin, A. Alekseev, I. Gushchin, J. M. Haro-Moreno, I. Chizhov, S. Siletsky, M. Mamedov, A. Rogachev, T. Balandin, V. Borshchevskiy, A. Popov, G. Bourenkov, E. Bamberg, F. Rodriguez-Valera, G. Büldt, V. Gordeliy, High-resolution structural insights into the heliorhodopsin family. *Proc. Natl. Acad. Sci. U.S.A.* **117**, 4131–4141 (2020).
